# A Hearing Intervention and Health-Related Quality of Life in Older Adults: A Secondary Analysis of the ACHIEVE Randomized Clinical Trial

**DOI:** 10.1001/jamanetworkopen.2024.46591

**Published:** 2024-11-21

**Authors:** Alison R. Huang, Emmanuel Garcia Morales, Michelle L. Arnold, Sheila Burgard, David Couper, Jennifer A. Deal, Nancy W. Glynn, Theresa Gmelin, Adele M. Goman, Lisa Gravens-Mueller, Kathleen M. Hayden, Christine M. Mitchell, James S. Pankow, James R. Pike, Nicholas S. Reed, Victoria A. Sanchez, Jennifer A. Schrack, Kevin J. Sullivan, Josef Coresh, Frank R. Lin, Theresa H. Chisolm

**Affiliations:** 1 Department of Epidemiology, Johns Hopkins Bloomberg School of Public Health, Baltimore, Maryland; 2 Cochlear Center for Hearing and Public Health, Johns Hopkins Bloomberg School of Public Health, Baltimore, Maryland; 3 College of Science and Mathematics, University of South Florida Sarasota–Manatee; 4 Department of Biostatistics, Gillings School of Global Public Health, University of North Carolina at Chapel Hill; 5 Department of Epidemiology, University of Pittsburgh School of Public Health, Pittsburgh, Pennsylvania; 6 School of Health and Social Care, Edinburgh Napier University, Edinburgh, United Kingdom; 7 Department of Social Sciences and Health Policy, Wake Forest University School of Medicine, Winston-Salem, North Carolina; 8 Division of Epidemiology and Community Health, University of Minnesota School of Public Health, Minneapolis; 9 Optimal Aging Institute, Department of Population Health and Medicine, New York University Grossman School of Medicine, New York University Langone Health, New York; 10 Department of Medicine: The MIND Center, The University of Mississippi Medical Center, Jackson

## Abstract

**Importance:**

Health-related quality of life is a critical health outcome and a clinically important patient-reported outcome in clinical trials. Hearing loss is associated with poorer health-related quality-of-life in older adults.

**Objective:**

To investigate the 3-year outcomes of hearing intervention vs health education control on health-related quality of life.

**Design, Setting, and Participants:**

This secondary analysis of a randomized clinical trial included participants treated for hearing loss at multiple US centers between 2018 and 2019 with 3-year follow-up completed in 2022. Eligible participants were aged 70 to 84 years, had untreated hearing loss, and were without substantial cognitive impairment. Participants were randomized (1:1) to hearing intervention or health education control and followed every 6 months.

**Intervention:**

Hearing intervention (provision of hearing aids and related technologies, counseling, education) or health education control (individual sessions covering topics relevant to chronic disease, disability prevention).

**Main Outcomes and Measures:**

Three-year change in the RAND-36 physical and mental health component scores over 3 years. The 8 individual domains of health-related quality-of-life were additionally assessed. Outcomes measured at baseline and at 6-month, 1-year, 2-year, and 3-year follow-ups. Intervention effect sizes estimated using a 2-level linear mixed effects model under the intention-to-treat principle.

**Results:**

A total of 977 participants were analyzed (mean [SD] age, 76.8 [4.0] years; 523 female [53.5%]; 112 Black [11.5%], 858 White [87.8%]; 521 had a Bachelor’s degree or higher [53.4%]), with 490 in the hearing intervention and 487 in the control group. Over 3 years, hearing intervention (vs health education control) had no significant association with physical (intervention, −0.49 [95% CI, −3.05 to 2.08]; control, −0.92 [95% CI, −3.39 to 1.55]; difference, 0.43 [95% CI, −0.64 to 1.51]) or mental (intervention, 0.38 [95% CI, −1.58 to 2.34]; control, −0.09 [95% CI, −1.99 to 1.81]; difference, 0.47 [95% CI, −0.41 to 1.35]) health-related quality of life.

**Conclusions and Relevance:**

In this secondary analysis of a randomized clinical trial, hearing intervention had no association with physical and mental health-related quality-of-life over 3 years among older adults with hearing loss. Additional intervention strategies may be needed to modify health-related quality among older adults with hearing loss.

**Trial Registration:**

ClinicalTrials.gov Identifier: NCT03243422

## Key Points

**Question:**

Is a hearing intervention associated with health-related quality-of-life over 3 years in older adults with hearing loss?

**Findings:**

In this secondary analysis of the ACHIEVE randomized clinical trial with 977 participants, a hearing intervention (vs health education control) was not associated with RAND-36 Health Survey physical and mental health-related quality of life changes over 3 years.

**Meaning:**

These results suggest that additional intervention strategies may be needed to modify health-related quality of life among older adults with hearing loss.

## Teaser

This secondary analysis of a randomized clinical trial examines the association of providing hearing aids and therapy following hearing loss vs health education with physical and mental quality of life.

## Introduction

The ACHIEVE study (Aging and Cognitive Health Evaluation in Elders) was a randomized clinical trial designed to test the effect of hearing intervention (provision of hearing aids and related technologies, counseling, and education) vs health education control (individual sessions with a health educator covering topics relevant to chronic disease and disability prevention) on 3-year cognitive decline in older adults with untreated hearing loss.^1^ In addition to the primary outcome of cognitive decline, health-related quality of life was also assessed as an exploratory outcome to evaluate other potential effects of hearing intervention. Health-related quality of life is a clinically important, patient-reported outcome in clinical trials.^2,3^ Health-related quality of life captures self-perceived benefits of an intervention across multiple domains (physical, emotional, and social well-being) that may not be captured by clinical outcomes.^3^ Greater health-related quality of life in older adults is also associated with lower morbidity (eg, lower risk of cardiovascular disease,^4^ cognitive decline, and dementia^5,6^) and mortality.^7,8,9^

Observational studies suggest associations between hearing loss and poorer mental and physical health-related quality of life.^10,11,12,13^ Hearing loss may lead to communication difficulties,^11,14^ cognitive decline and dementia,^15,16,17,18,19^ depression,^20,21^ reduced physical activity^22,23^ and function,^24^ and loneliness and social isolation,^25,26,27,28,29^ potentially resulting in poorer emotional, social, and physical health. Treatment of hearing loss could potentially improve health-related quality of life among older adults with hearing loss. The effect of hearing intervention on health-related quality of life has only been investigated in 2 prior randomized clinical trials, to our knowledge.^30,31^ Evidence is mixed and limited by short duration of follow-up (1 year), restriction to specific study populations (both studies conducted in male, veteran populations), and lack of an active control group (both studies used no-intervention, waitlisted control groups).^30,31^

We report results from a secondary analysis of the ACHIEVE study to examine the association of hearing intervention vs health education control with health-related quality of life over 3 years among community-dwelling older adults with untreated hearing loss. Health-related quality of life was gathered as a prespecified, exploratory outcome in the ACHIEVE study. The ACHIEVE study is the largest and longest randomized clinical trial of which we are aware to assess hearing intervention and health-related quality of life.

## Methods

### Study Design and Participants

The ACHIEVE study is a 3-year, multicenter, randomized clinical trial designed to test the effect of hearing intervention^32,33^ vs health education control on 3-year cognitive decline among older adults with untreated hearing loss (ClinicalTrials.gov: NCT03243422). ACHIEVE is partially nested within the scientific and physical infrastructure of the Atherosclerosis Risk in Communities (ARIC) study,^34^ an ongoing observational study conducted at 4 field sites in the US (Forsyth County, North Carolina; Jackson, Mississippi; Minneapolis suburbs, Minnesota; Washington County, Maryland).^34,35^

ACHIEVE participants were recruited from 2 sources at each field site: (1) existing ARIC study participants and (2) de novo healthy volunteers from the community. Inclusion criteria were aged 70 to 84 years, having adult-onset bilateral hearing loss (better-ear 4-frequency [0.5-4 kHz] pure tone average [PTA]^3^ 30 dB HL [decibel hearing level] and below 70 dB HL), without substantial cognitive impairment (Mini-Mental State Examination [MMSE] score 23 or above for participants with a high school degree or less, 25 or above for participants with some college education or more), word recognition score in quiet of 60% correct or higher in the better-hearing ear, community-dwelling, and fluent English speaker. Exclusion criteria were self-reported disability in 2 or more activities of daily living, presenting visual acuity (with correction) worse than 20/63 on the MNREAD acuity chart (Precision Vision; corresponding to inability to comfortably read 14-point font), self-reported hearing aid use in the past year, permanent bilateral conductive hearing loss, medical contraindication to hearing aid use, or unwillingness to wear hearing aids on a regular basis.^34^

The ACHIEVE trial was approved by the institutional review boards of all participating study sites and academic centers. Participants provided written informed consent. Reporting followed the Consolidated Standards of Reporting Trials (CONSORT) reporting guideline. The trial protocol appears as Supplement 1.

### Procedures

Participants were randomly assigned (1:1) to either hearing intervention or a health education control intervention at baseline (2018-2019). Randomization was stratified by severity of hearing loss (PTA below 40 dB, 40 dB or higher), recruitment source (ARIC or de novo), and field site. Participant spouses or partners were randomly assigned as a unit. Intervention assignment was, by nature, unmasked to participants and study staff. However, participants were masked to the study hypothesis and informed that both interventions could promote healthy aging. Participants were randomly assigned to hearing intervention or health education control at baseline and would receive the other intervention after the 3-year follow-up visit. The trial’s study design and methods have been previously published.^34^

The hearing intervention consisted of four 1-hour sessions with a study audiologist every 1 to 3 weeks postrandomization. Participants received bilateral hearing aids that were fit to prescriptive targets using real-ear measures. The intervention included education on device use and counseling on self-management and communication strategies. Participants also received other accompanying hearing assistive technologies (eg, devices to stream cell phones and television, remote microphones) based on individual preference and listening needs. Booster sessions every 6 months provided reinstruction for device use and hearing rehabilitative strategies. Details regarding the hearing intervention have been previously published.^32,33^

The health education control also consisted of four 1-hour sessions with a certified health educator every 1 to 3 weeks postrandomization and was designed to match the intensity and general levels of participant time and attention as the hearing intervention. The health education control followed the 10 Keys to Healthy Aging program,^36^ an evidence-based interactive health education program for adults aged 65 years and older on 10 topics relevant to chronic disease and disability prevention. Sessions were customized to each participant and included didactic education and activities, goal setting, and optional extracurricular enrichment activities and a 5- to 10-minute upper body extremity stretching program. Participants attended booster sessions every 6 months. The 10 Keys to Healthy Aging program has been implemented as the control intervention in prior trials.^37,38^

Participants were followed every 6 months. From March 2020 to June 2021, study sites were closed for in-person study visits due to the COVID-19 pandemic and modified phone-based intervention and assessment of study outcomes was conducted.^1,34^

### Health-Related Quality-of-Life Outcomes

Health-related quality of life was a prespecified exploratory outcome of the ACHIEVE study and measured by the RAND-36 Health Survey.^39^ The RAND-36 Health Survey is psychometrically validated and consists of 36 questions about 8 domains of health: physical functioning, role limitations due to physical problems, role limitations due to emotional problems, energy or fatigue, emotional well-being, social functioning, pain, and general health (Cronbach α > .73 for all domains).^39^ Participants were asked to consider their health over the past 4 weeks and to also consider hearing as part of their health. Each of the 8 domain scores have a range of zero to 100 with higher scores indicating better health-related quality of life. The RAND-36 Health Survey was administered at baseline and at the 6-month, 1-year, 2-year, and 3-year follow-up visits.

Two summary scores (physical health component summary score, mental health component summary score) were calculated using an established summary component scoring algorithm.^40^ Each of the 8 domain scores were also assessed. Scores range from zero to 100 (higher scores indicating better physical and mental health-related quality of life).^40^

### Covariates

Covariates measured at baseline were age, sex (male, female), education (elementary or some high school, completed high school or some college, Bachelor’s degree or greater), marital status (married vs single, divorced, or widowed), hearing loss severity (4-frequency [0.5, 1, 2, and 4 kHz] PTA for the better-hearing ear), global cognition, recruitment source (ARIC, de novo cohorts), field site (Forsyth County, North Carolina; Jackson, Mississippi; Minneapolis, Minnesota; or Washington County, Maryland), and whether the participant was part of a recruited spousal pair. Race was measured by self-report, and was not included as a covariate because there is no observed or hypothesized association between race and the study outcome. Given the potential impact of the COVID-19 global pandemic and related lockdowns on health-related quality of life, we included a covariate to adjust for the pandemic start (binary covariate taking the value of zero if the outcome was measured before March 13, 2020 [date COVID-19 pandemic national emergency declared in the US] and taking the value of 1 if the outcome was measured on or after March 13, 2020) and a linear spline at June 30, 2021 (ACHIEVE study field sites reopened) to account for the gradual emergence from pandemic related lockdowns.

### Statistical Analysis

Participant characteristics by randomization group were described. The association of hearing intervention with 3-year change in physical and mental health-related quality-of-life component score was estimated using a 2-level linear mixed effects model with an independent covariance matrix under the intention-to-treat principle. Time was modeled continuously. Restricted maximum likelihood with a Kenward-Roger correction was used to generate parameter estimates, 95% CI, and *P* values. The fully adjusted model included a binary variable for intervention assignment, time from baseline, the interaction between intervention assignment and time, and covariates measured at baseline (age, sex, education, marital status, hearing loss severity, global cognition, recruitment source, field site, and whether the participant was part of a recruited spousal pair), and the interaction between time and all covariates. In secondary analysis, analyses were repeated to assess the association of hearing intervention with 3-year change in each domain of health-related quality of life (physical functioning, physical role limitation, emotional role limitation, energy or fatigue, emotional well-being, social functioning, pain, general health).

Missing covariate and health-related quality-of-life domain scores due to incomplete items or loss to follow-up were imputed using multiple imputation by chained equations. Postdeath assessments were excluded from imputation. Health-related quality-of-life domain scores at baseline and 6-month, 1-year, 2-year, and 3-year follow-up visits (5 assessments over 3 years) were imputed (20 sets of imputations and 100 burn-in period interactions) separately and included all covariates from the fully adjusted model, as well as: age (squared); interaction terms between age; self-reported race, and sex, time from baseline; and a 3-way interaction between time, intervention group, and recruitment source. Future health-related quality-of-life measures were excluded from the imputation model. Both physical and mental health component summary scores were computed postimputation of individual domain scores.

In sensitivity analyses, we assessed the per-protocol and complier average causal effect using a 2-stage least squares approach, conducted a complete case analysis, and stratified analyses by recruitment source (ARIC, de novo). Per-protocol analyses were limited to the subset of participants who completed the intervention, had no hearing aid intervention drop-in or drop-out, and had no major protocol deviations (824 participants total, 391 in the control group and 433 in the intervention group). Health-related quality of life was a prespecified, exploratory outcome of the ACHIEVE study and analyses were considered hypothesis-generating rather than hypothesis-testing. Thus, we focus on the patterns of effect across outcomes instead of evaluating statistical significance. All analyses were conducted using Stata 18.0 (StataCorp).

## Results

At baseline, 977 participants were included (mean [SD] age, 76.8 [4.0] years; 523 female [53.5%]; 112 Black [11.5%], 858 White [87.8%]); 521 (53.4%) had a Bachelor’s degree or higher and 602 (61.6%) were married (Table 1). Participants had baseline mean (SD) MMSE score of 28.22 (1.62) and mean (SD) better ear PTA of 39.42 dB HL (6.91). Participants were recruited from the ARIC study (238 [24.4%]) and de novo (739 [75.6%]) and were randomly assigned to hearing intervention (490 [50.2%]) or health education control (487 [49.9%]). Participant characteristics were similar across intervention assignment at baseline. Mean (SD) physical health component score was 44.78 (9.81) and mental health component score was 56.28 (6.62) at baseline (Table 2).

**Table 1.** Baseline Participant Characteristics by Intervention Assignment in the ACHIEVE Study

Characteristics	Participants, No. (%)^a^		
	Total (N = 977)	Hearing intervention (n = 490)	Health education control (n = 487)
Age, mean (SD), y	76.8 (4.0)	76.5 (3.9)	77.0 (4.0)
Sex			
Male	454 (46.5)	226 (46.1)	228 (46.8)
Female	523 (53.5)	264 (53.9)	259 (53.2)
Self-reported race			
Black	112 (11.5)	53 (10.8)	59 (12.1)
White	858 (87.8)	434 (88.6)	424 (87.1)
Other^b^	7 (0.7)	3 (0.61%)	4 (0.8)
Education			
Less than high school	37 (3.8)	19 (3.9)	18 (3.7)
High school, GED, or vocational school	418 (42.8)	206 (42.1)	212 (43.5)
College, graduate, or professional school	521 (53.4)	264 (54.0)	257 (52.8)
Marital status			
Married	602 (61.6)	294 (60.0)	308 (63.2)
Not married	375 (38.4)	196 (40.0)	179 (36.8)
Income			
<$25 000	147 (15.5)	73 (15.3)	74 (15.7)
$25 000-$49 999	283 (29.8)	156 (32.6)	127 (26.9)
$50 000-$74 999	210 (22.1)	91 (19.0)	119 (25.2)
$75 000-$100 000	140 (14.7)	68 (14.2)	72 (15.3)
>$100 000	170 (17.9)	90 (18.8)	80 (17.0)
Better ear PTA, mean (SD), dB HL	39.42 (6.91)	39.54 (7.07)	39.30 (6.75)
Mini-Mental State Examination score, mean (SD)	28.22 (1.62)	28.22 (1.63)	28.21 (1.61)
Global Cognition Factor Score, mean (SD)	0.00 (0.93)	0.01 (0.95)	−0.01 (0.90)
Field site			
Forsyth County, NC	236 (24.2)	117 (23.9)	119 (24.4)
Jackson, MS	243 (24.9)	120 (24.5)	123 (25.3)
Minnesota Suburbs, MN	236 (24.2)	120 (24.5)	116 (23.8)
Washington County, MD	262 (26.8)	133 (27.1)	129 (26.5)
Recruitment source			
ARIC	238 (24.4)	120 (24.5)	118 (24.2)
De novo	739 (75.6)	370 (75.5)	369 (75.8)
Participant part of a recruited spousal pair	90 (9.2)	46 (9.4)	44 (9.0)

Abbreviations: ACHIEVE, Aging and Cognitive Health Evaluation in Elders; ARIC, Atherosclerosis Risk in Communities; dB HL, decibels hearing level; GED, general educational development credential; PTA, pure tone average.

^a^ A total of 1 participant (in the hearing intervention group) was missing information about educational attainment. A total of 27 participants (12 hearing intervention, 15 control) were missing information about household income.

^b^ Racial and ethnic subcategories included in other race were American Indian, Asian, Native American, Native Hawaiian, and Pacific Islander.

**Table 2.** Baseline and Year-3 RAND-36 Health Survey Domain Scores by Intervention Assignment in the ACHIEVE Study

RAND-36 Health Survey domains	Survey scores, mean (SD)		
	Total (N = 977)	Hearing intervention (n = 490)	Health education control (n = 487)
**Component scores**			
Physical health component score			
Baseline (n = 976)	44.75 (9.81)	44.85 (9.90)	44.65 (9.74)
Year 3 (n = 871)	42.43 (10.94)	42.96 (10.76)	41.90 (11.10)
Mental health component score			
Baseline (n = 976)	56.28 (6.62)	56.55 (6.32)	56.02 (6.89)
Year 3 (n = 871)	55.94 (7.61)	56.47 (7.62)	55.42 (7.57)
**Domain scores**			
Physical functioning			
Baseline (n = 977)	75.19 (22.11)	75.95 (21.93)	74.43 (22.28)
Year 3 (n = 871)	68.70 (25.33)	69.51 (25.21)	67.89 (25.45)
Role limitations from physical problems			
Baseline (n = 977)	70.68 (37.29)	71.48 (37.35)	69.87 (37.26)
Year 3 (n = 871)	64.75 (40.07)	67.16 (39.31)	62.33 (40.72)
Role limitations from emotional problems			
Baseline (n = 977)	90.58 (23.38)	91.50 (22.21)	89.67 (24.48)
Year 3 (n = 871)	89.11 (26.22)	89.55 (25.65)	88.67 (26.81)
Energy or fatigue			
Baseline (n = 976)	63.69 (18.83)	64.39 (19.13)	62.97 (18.53)
Year 3 (n = 871)	60.21 (19.43)	62.29 (19.45)	58.11 (19.20)
Emotional well-being			
Baseline (n = 976)	85.44 (12.04)	86.09 (12.00)	84.78 (12.06)
Year 3 (n = 871)	84.14 (13.11)	84.90 (13.42)	83.38 (12.75)
Social functioning			
Baseline (n = 977)	87.77 (18.45)	87.86 (18.57)	87.68 (18.35)
Year 3 (n = 871)	84.30 (20.65)	86.41 (19.90)	82.17 (21.19)
Pain			
Baseline (n = 977)	73.41 (22.69)	74.23 (22.91)	72.58 (22.46)
Year 3 (n = 871)	70.59 (24.40)	71.89 (24.41)	69.29 (24.35)
General health			
Baseline (n = 977)	69.41 (17.48)	68.62 (18.06)	70.20 (16.86)
Year 3 (n = 871)	66.91 (18.26)	67.29 (19.06)	66.52 (17.43)

Abbreviation: ACHIEVE, Aging and Cognitive Health Evaluation in Elders.

**Figure 1.**
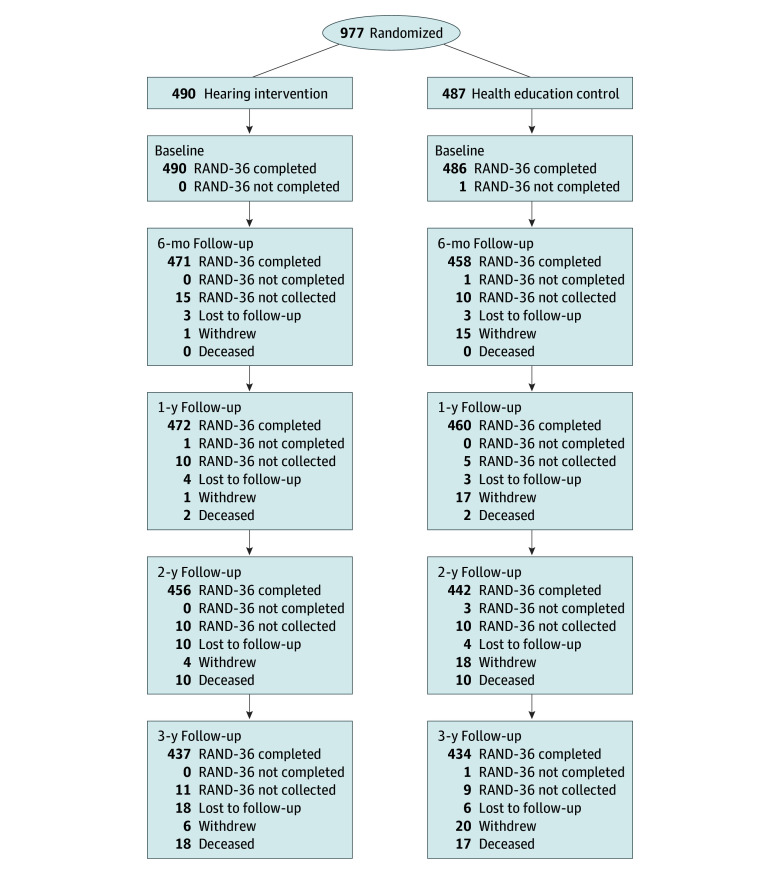
Trial Profile RAND-36 indicates the RAND-36 Health Survey.

At the end of the study period (3-year follow-up), 871 participants (89.2%) had complete data for RAND-36 Health Survey measures (Figure 1). Of the 106 participants who did not have complete RAND-36 Health Survey data at the 3-year visit, 1 (0.9%) had incomplete RAND-36 Health Survey data, 20 (18.9%) did not complete the RAND-36 Health Survey, 24 (22.6%) were lost to follow-up, 26 (24.5%) withdrew from the study, and 35 (33.0%) died. Over the study period, 10 (2.0%) participants in the hearing intervention dropped out (eg, discontinued hearing aid use) and 76 participants (15.6%) in the health education control dropped in (eg, obtained hearing aids outside of the study).

**Figure 2.**
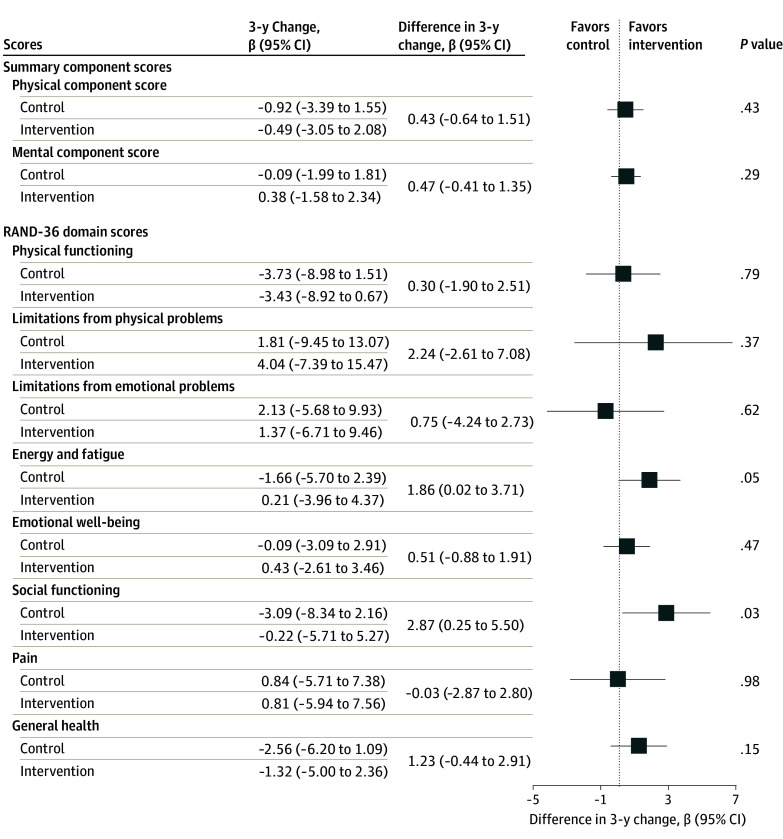
Covariate-Adjusted Analysis of 3-Year Change in RAND-36 Health-Related Quality of Life Physical and Mental Health Component Summary Scores and Domain Scores by Intervention Assignment in the ACHIEVE Study Higher RAND-36 Health Survey health-related quality of life domain scores and physical and mental health component scores represent better health-related quality of life. A positive value for the difference in 3-year domain scores between hearing intervention and control indicates a positive effect of hearing intervention; a negative value for the difference in 3-year domain scores between hearing intervention and control indicates a positive effect of the health education control. Models adjusted for covariates measured at baseline (age, sex, education, marital status, hearing loss severity, global cognition, recruitment source, field site, and whether the participant was part of a recruited spousal pair), and the interaction between time and all covariates. ACHIEVE indicates Aging and Cognitive Health Evaluation in Elders.

Differences between intervention and control in 3-year change in the physical and mental health-related quality of life component scores and each domain of health-related quality of life are presented (Figure 2). With higher domain scores representing better health-related quality of life, a positive difference score between hearing intervention and control indicates a beneficial effect of hearing intervention while a negative difference score indicates a beneficial effect of control. The hearing intervention was not associated with component scores of physical (intervention, −0.49 [95% CI, −3.05 to 2.08]; control, −0.92 [95% CI, −3.39 to 1.55]; difference, 0.43 [95% CI, −0.64 to 1.51]) and mental (intervention, 0.38 [95% CI, −1.58 to 2.34]; control, −0.09 [95% CI, −1.99 to 1.81]; difference, 0.47 [95% CI, −0.41 to 1.35]) health-related quality of life.

Among the 8 individual domains of health-related quality of life, a decline in social functioning over 3 years was observed in both hearing intervention (3-year change, −0.22 [95% CI, −5.71 to 5.27]) and health education control (3-year change, −3.09 [95% CI, −8.34 to 2.16]); however, the 3-year rate of decline was slower among participants in the hearing intervention (difference, 2.87 [95% CI, 0.25 to 5.50]). This finding suggests a positive association of hearing intervention with reducing declines in social functioning. Additionally, energy increased (less fatigue) in the hearing intervention (3-year change, 0.21 [95% CI, −3.96 to 4.37]) yet declined (more fatigue) in the health education control (3-year change, −1.66 [95% CI, −5.70 to 2.39]) over 3 years, suggesting hearing intervention also had a beneficial association with increased energy (less fatigue) over 3 years (difference, 1.86 [95% CI, 0.02 to 3.71]). The differences between the hearing intervention vs health education controls groups for limitations from physical problems (difference, 2.24 [95% CI, −2.61 to 7.08]), emotional well-being (difference, 0.51 [95% CI, −0.88 to 1.91]), and general health (difference, 1.23 [95% CI, −0.44 to 2.91]) health-related quality of life suggested a potential positive benefit of hearing intervention but with confidence intervals that include the null value (Figure 2).

In sensitivity analyses stratified by recruitment source, the hearing intervention was positively associated with general health in the ARIC cohort and with social functioning and increased energy (less fatigue) in the de novo cohort but there was no evidence of an interaction effect by recruitment source (eFigure 1 in Supplement 2). The direction of association of hearing intervention outcomes were also largely similar in per-protocol analyses (eFigure 2 in Supplement 2), complier average causal effect (eFigure 3 in Supplement 2), and complete case analyses (eFigure 4 in Supplement 2).

## Discussion

In a secondary analysis of the ACHIEVE study, hearing intervention was not associated with physical and mental health-related quality of life over 3 years. A suggested benefit of hearing intervention on reducing declines in social functioning and fatigue was observed; however, magnitude of effect is likely small. Additional intervention strategies may be needed to modify health-related quality of life among older adults with hearing loss. To our knowledge, the ACHIEVE study represents the largest and longest randomized clinical trial to study the association of hearing intervention with health-related quality of life.

Evidence from previous observational studies has suggested that hearing aid use may positively affect health-related quality of life.^41,42,43,44,45^ However, existing evidence on hearing intervention and health-related quality of life from randomized clinical trials is limited and mixed.^30,31,46,47^ To our knowledge, only 2 randomized clinical trials, both conducted in veteran populations, have tested the effect of hearing treatment with hearing aids on health-related quality of life.^30,31^ McArdle et al^30^ evaluated the effect of hearing aid intervention on health-related quality of life in a cohort of 380 veterans in a multisite study in the US. In the hearing intervention group, the trial observed lower mean scores (indicating better health-related quality of life) in both the World Health Organization Disability Assessment Schedule (WHO-DAS II) total score and in the communication and participation domain scores at 10-weeks postintervention. In the delayed treatment group, mean WHO-DAS II total and communication and participation domain scores increased (indicating poorer health-related quality of life) over 10 weeks.^30^ In another randomized clinical trial of 194 veterans followed for 4 months, no difference between hearing aid intervention and waitlist control groups was observed in the Self-Evaluation of Life Function (SELF) scale, a broad health-related quality-of-life measure that assesses physical disability, social satisfaction, symptoms of aging, depression, self-esteem, and personal control.^31^ However, benefit of hearing aid use was observed on self-perceived social and emotional effects of hearing loss (using the Hearing Handicap Inventory for the Elderly) and on communication function (Quantified Denver Scale).^31^

In the health-related quality of life domain-specific findings, the ACHIEVE study observed a potential benefit of hearing intervention on reducing fatigue and reducing declines in social functioning. The primary hypothesized mechanisms through which hearing loss may worsen fatigue is increased cognitive load. Cognitive load (ie, information degradation hypothesis^48^) posits that, with hearing loss, greater cognitive resources are diverted to processing speech and sound, leaving fewer cognitive resources available for other cognitive functions (eg, memory, executive function); fatigue may be a symptom of high cognitive load.^49,50^ Furthermore, hearing loss may worsen social functioning through communication difficulties, withdrawal from participation in social activities, lower physical activity and physical function, and depression.^25,26,28,29,51,52^ Hearing loss may also impact confidence and perceived capacity to engage with others.

### Limitations

Limitations of the ACHIEVE study include the inability to feasibly mask intervention assignment, which may bias how participants self-respond to questions about their well-being. However, participants were masked to the study hypothesis and were informed that both interventions were designed to promote healthy aging. Participants were also informed at randomization that they would receive the other intervention after the 3-year study period. Health-related quality of life was also a prespecified exploratory outcome of the ACHIEVE study and was not a specified primary or secondary outcome. Thus, the ACHIEVE study was not designed or powered to test for effects of hearing intervention on health-related quality of life. As such, findings from the current study focus on patterns of results across outcomes and thus we did not adjust for multiple comparisons. Results should be considered as hypothesis-generating rather than hypothesis-testing. Furthermore, while magnitude of effect size is interpreted as a per-unit difference on domains of the RAND-36 Health Survey, magnitude of clinical significance is unknown.

While there was no association of hearing intervention with health-related quality of life, hearing intervention has been shown to improve communicative function^53^ and slow cognitive decline^1^ in certain populations. Additional intervention strategies, potentially in combination with hearing intervention, may be needed to modify health-related quality of life among older adults with hearing loss. Hearing intervention is scalable, confers little to no medical risk, and, given the high prevalence of hearing loss (65% of adults aged 71 years and older in the US have hearing loss),^54^ has the potential to impact a large proportion of older adults. Further research is needed to understand how hearing intervention may be incorporated into additional strategies for supporting health and well-being in older adults with hearing loss. Additionally, the ACHIEVE study hearing intervention was a comprehensive program of audiological care and may not be represent the standard level of care delivered in the community. Future research aims to evaluate the effectiveness of hearing intervention on slowing cognitive decline in clinical settings.

## Conclusions

In this secondary analysis of a randomized clinical trial, hearing intervention was not associated with physical and mental health-related quality of life over 3 years compared with health education control. Future efforts are needed to determine strategies for modifying health-related quality of life in older adults with hearing loss.

## Notes

Supplement 1.
Trial Protocol and Statistical Analysis Plan

Supplement 2. **eFigure 1.** Covariate-Adjusted Analyses Stratified by Recruitment Source (ARIC Cohort [n = 238], De Novo Cohort [n = 739]) of 3-Year Change in RAND-36 Health-Related Quality of Life Physical and Mental Health Component Summary Scores and Domain Scores by Intervention Assignment, ACHIEVE Study **eFigure 2.** Per-Protocol Analysis of 3-Year Change in RAND-36 Health-Related Quality of Life Physical and Mental Health Component Summary Scores and Domain Scores by Intervention Assignment, ACHIEVE study (n = 824) **eFigure 3.** Complier Average Causal Effect Analysis of 3-Year Change in RAND-36 Health-Related Quality of Life Physical and Mental Health Component Summary Scores and Domain Scores by Intervention Assignment, ACHIEVE study (n = 977) **eFigure 4.** Complete Case Analysis of 3-Year Change in RAND-36 Health-Related Quality of Life Physical and Mental Health Component Summary Scores and Domain Scores by Intervention Assignment, ACHIEVE study (n = 977)

Supplement 3. **Nonauthor Collaborators.** ACHIEVE Collaborative Research Group

Supplement 4.
Data Sharing Statement

## References

[1] Lin FR, Pike JR, Albert MS, ; ACHIEVE Collaborative Research Group. Hearing intervention versus health education control to reduce cognitive decline in older adults with hearing loss in the USA (ACHIEVE): a multicentre, randomised controlled trial. Lancet. 2023;402(10404):786-797. doi:10.1016/S0140-6736(23)01406-X 37478886 PMC10529382

[2] Karimi M, Brazier J. Health, health-related quality of life, and quality of life: what is the difference? Pharmacoeconomics. 2016;34(7):645-649. doi:10.1007/s40273-016-0389-9 26892973

[3] Haywood KL, Garratt AM, Fitzpatrick R. Quality of life in older people: a structured review of generic self-assessed health instruments. Qual Life Res. 2005;14(7):1651-1668. doi:10.1007/s11136-005-1743-0 16119178

[4] Phyo AZZ, Ryan J, Gonzalez-Chica DA, ; ASPREE Investigator Group. Health-related quality of life and incident cardiovascular disease events in community-dwelling older people: a prospective cohort study. Int J Cardiol. 2021;339:170-178. doi:10.1016/j.ijcard.2021.07.004 34245793 PMC9993351

[5] Phyo AZZ, Gonzalez-Chica DA, Stocks NP, ; ASPREE Investigator Group. The utility of assessing health-related quality of life to predict cognitive decline and dementia. J Alzheimers Dis. 2021;80(2):895-904. doi:10.3233/JAD-201349 33579847 PMC8093030

[6] Ding X, Abner EL, Schmitt FA, Crowley J, Goodman P, Kryscio RJ. Mental Component Score (MCS) from health-related quality of life predicts incidence of dementia in US males. J Prev Alzheimers Dis. 2021;8(2):169-174. 33569563 10.14283/jpad.2020.50 PMC8162937

[7] Brown DS, Thompson WW, Zack MM, Arnold SE, Barile JP. Associations between health-related quality of life and mortality in older adults. Prev Sci. 2015;16(1):21-30. doi:10.1007/s11121-013-0437-z 24189743 PMC4593240

[8] Dominick KL, Ahern FM, Gold CH, Heller DA. Relationship of health-related quality of life to health care utilization and mortality among older adults. Aging Clin Exp Res. 2002;14(6):499-508. doi:10.1007/BF03327351 12674491

[9] Tsai SY, Chi LY, Lee CH, Chou P. Health-related quality of life as a predictor of mortality among community-dwelling older persons. Eur J Epidemiol. 2007;22(1):19-26. doi:10.1007/s10654-006-9092-z 17216549

[10] Tseng YC, Liu SHY, Lou MF, Huang GS. Quality of life in older adults with sensory impairments: a systematic review. Qual Life Res. 2018;27(8):1957-1971. doi:10.1007/s11136-018-1799-2 29404924

[11] Dalton DS, Cruickshanks KJ, Klein BE, Klein R, Wiley TL, Nondahl DM. The impact of hearing loss on quality of life in older adults. Gerontologist. 2003;43(5):661-668. doi:10.1093/geront/43.5.661 14570962

[12] Chia EM, Wang JJ, Rochtchina E, Cumming RR, Newall P, Mitchell P. Hearing impairment and health-related quality of life: the Blue Mountains Hearing Study. Ear Hear. 2007;28(2):187-195. doi:10.1097/AUD.0b013e31803126b6 17496670

[13] Wong LL, Cheng LK. Quality of life in older Chinese-speaking adults with hearing impairment. Disabil Rehabil. 2012;34(8):655-664. doi:10.3109/09638288.2011.619614 22080747

[14] Garstecki DC, Erler SF. Older adult performance on the Communication Profile for the Hearing Impaired: gender difference. J Speech Lang Hear Res. 1999;42(4):785-796. doi:10.1044/jslhr.4204.785 10450900

[15] Livingston G, Huntley J, Sommerlad A, . Dementia prevention, intervention, and care: 2020 report of the Lancet Commission. Lancet. 2020;396(10248):413-446. doi:10.1016/S0140-6736(20)30367-6 32738937 PMC7392084

[16] Huang AR, Jiang K, Lin FR, Deal JA, Reed NS. Hearing loss and dementia prevalence in older adults in the US. JAMA. 2023;329(2):171-173. doi:10.1001/jama.2022.20954 36625819 PMC9856835

[17] Lin FR, Metter EJ, O’Brien RJ, Resnick SM, Zonderman AB, Ferrucci L. Hearing loss and incident dementia. Arch Neurol. 2011;68(2):214-220. doi:10.1001/archneurol.2010.362 21320988 PMC3277836

[18] Deal JA, Betz J, Yaffe K, . Hearing impairment and incident dementia and cognitive decline in older adults: the Health ABC Study. Gerontol Series A. 2017;72(5):703-709. 10.1093/gerona/glw069 PMC5964742 27071780

[19] Loughrey DG, Kelly ME, Kelley GA, Brennan S, Lawlor BA. Association of age-related hearing loss with cognitive function, cognitive impairment, and dementia: a systematic review and meta-analysis. JAMA Otolaryngol Head Neck Surg. 2018;144(2):115-126. doi:10.1001/jamaoto.2017.2513 29222544 PMC5824986

[20] Rutherford BR, Brewster K, Golub JS, Kim AH, Roose SP. Sensation and psychiatry: linking age-related hearing loss to late-life depression and cognitive decline. Am J Psychiatry. 2018;175(3):215-224. doi:10.1176/appi.ajp.2017.17040423 29202654 PMC5849471

[21] Huang AR, Reed NS, Deal JA, . Depression and health-related quality of life among older adults with hearing loss in the ACHIEVE study. J Applied Gerontol. 2024;43(5):550-561. doi:10.1177/07334648231212291 38016096 PMC10981564

[22] Martinez-Amezcua P, Suen JJ, Lin F, Schrack JA, Deal JA. Hearing impairment and objectively measured physical activity: a systematic review. J Am Geriatr Soc. 2022;70(1):301-304. doi:10.1111/jgs.17529 34713440 PMC8742764

[23] Martinez-Amezcua P, Dooley EE, Reed NS, . Association of hearing impairment and 24-hour total movement activity in a representative sample of US adults. JAMA Netw Open. 2022;5(3):e222983-e222983. doi:10.1001/jamanetworkopen.2022.2983 35302630 PMC8933734

[24] Martinez-Amezcua P, Kuo PL, Reed NS, Association of hearing impairment with higher level physical functioning and walking endurance: results from the Baltimore Longitudinal Study of Aging (BLSA). J Gerontol A Biol Sci Med Sci. 2021;76(10):e290-e298. doi:10.1093/gerona/glab144 34003883 PMC8436975

[25] Huang AR, Deal JA, Rebok GW, Pinto JM, Waite L, Lin FR. Hearing impairment and loneliness in older adults in the United States. J Appl Gerontol. 2021;40(10):1366-1371. doi:10.1177/0733464820944082 32749194

[26] Huang AR, Reed NS, Deal JA, . Loneliness and social network characteristics among older adults with hearing loss in the ACHIEVE Study. J Gerontol Series A. 2024;79(2):glad196. doi:10.1093/gerona/glad196 PMC10809043 37578190

[27] Mick P, Kawachi I, Lin FR. The association between hearing loss and social isolation in older adults. Otolaryngol Head Neck Surg. 2014;150(3):378-384. doi:10.1177/0194599813518021 24384545

[28] Shukla A, Cudjoe TK, Lin FR, Reed NS. Functional hearing loss and social engagement among Medicare beneficiaries. J Gerontol B Psychol Sci Soc Sci. 2021;76(1):195-200. doi:10.1093/geronb/gbz094 31359056 PMC7756723

[29] Sung YK, Li L, Blake C, Betz J, Lin FR. Association of hearing loss and loneliness in older adults. J Aging Health. 2016;28(6):979-994. doi:10.1177/0898264315614570 26597841

[30] McArdle R, Chisolm TH, Abrams HB, Wilson RH, Doyle PJ. The WHO-DAS II: measuring outcomes of hearing aid intervention for adults. Trends Amplif. 2005;9(3):127-143. doi:10.1177/108471380500900304 16244759 PMC4111523

[31] Mulrow CD, Aguilar C, Endicott JE, . Quality-of-life changes and hearing impairment:a randomized trial. Ann Intern Med. 1990;113(3):188-194. doi:10.7326/0003-4819-113-3-188 2197909

[32] Arnold ML, Haley W, Lin FR, . Development, assessment, and monitoring of audiologic treatment fidelity in the aging and cognitive health evaluation in elders (ACHIEVE) randomised controlled trial. Int J Audiol. 2022;61(9):720-730. doi:10.1080/14992027.2021.1973126 34533430 PMC11992692

[33] Sanchez VA, Arnold ML, Reed NS, . The Hearing Intervention for the Aging and Cognitive Health Evaluation in Elders randomized control trial: manualization and feasibility study. Ear Hear. 2020;41(5):1333-1348. doi:10.1097/AUD.0000000000000858 32251012 PMC10436703

[34] Deal JA, Goman AM, Albert MS, . Hearing treatment for reducing cognitive decline: design and methods of the Aging and Cognitive Health Evaluation in Elders randomized controlled trial. Alzheimers Dement (N Y). 2018;4:499-507. doi:10.1016/j.trci.2018.08.007 30364572 PMC6197326

[35] Wright JD, Folsom AR, Coresh J, . The ARIC (atherosclerosis risk in communities) study: JACC focus seminar 3/8. J Am Coll Cardiol. 2021;77(23):2939-2959. doi:10.1016/j.jacc.2021.04.035 34112321 PMC8667593

[36] Newman AB, Bayles CM, Milas CN, . The 10 keys to healthy aging: findings from an innovative prevention program in the community. J Aging Health. 2010;22(5):547-566. doi:10.1177/0898264310363772 20495156 PMC4896138

[37] Morone NE, Greco CM, Rollman BL, . The design and methods of the aging successfully with pain study. Contemp Clin Trials. 2012;33(2):417-425. doi:10.1016/j.cct.2011.11.012 22115971 PMC3268822

[38] Venditti EM, Zgibor JC, Vander Bilt J, . Mobility and Vitality Lifestyle Program (MOVE UP): a community health worker intervention for older adults with obesity to improve weight, health, and physical function. Innov Aging. 2018;2(2):igy012. doi:10.1093/geroni/igy012 30480135 PMC6176958

[39] Hays RD, Morales LS. The RAND-36 Measure of Health-Related Quality of Life. RAND research and commentary archive. Published online 2001. Accessed June 22, 2022. https://www.rand.org/pubs/reprints/RP971.html 10.3109/07853890109002089 11491194

[40] Taft C, Karlsson J, Sullivan M. Do SF-36 summary component scores accurately summarize subscale scores? Qual Life Res. 2001;10(5):395-404. doi:10.1023/A:1012552211996 11763202

[41] Chisolm TH, Johnson CE, Danhauer JL, . A systematic review of health-related quality of life and hearing aids: final report of the American Academy of Audiology Task Force On the Health-Related Quality of Life Benefits of Amplification in Adults. J Am Acad Audiol. 2007;18(2):151-183. doi:10.3766/jaaa.18.2.7 17402301

[42] Contrera KJ, Betz J, Li L, . Quality of life after intervention with a cochlear implant or hearing aid. Laryngoscope. 2016;126(9):2110-2115. doi:10.1002/lary.25848 26775283 PMC4947575

[43] Mondelli MFCG, Souza PJ. Quality of life in elderly adults before and after hearing aid fitting. Braz J Otorhinolaryngol. 2012;78(3):49-56. doi:10.1590/S1808-86942012000300010 22714847 PMC9446233

[44] Borre ED, Kaalund K, Frisco N, . The impact of hearing loss and its treatment on health-related quality of life utility: a systematic review with meta-analysis. J Gen Intern Med. 2023;38(2):456-479. doi:10.1007/s11606-022-07795-9 36385406 PMC9905346

[45] Johnson CE, Danhauer JL, Ellis BB, Jilla AM. Hearing aid benefit in patients with mild sensorineural hearing loss: a systematic review. J Am Acad Audiol. 2016;27(4):293-310. doi:10.3766/jaaa.14076 27115240

[46] Ferguson MA, Kitterick PT, Chong LY, Edmondson-Jones M, Barker F, Hoare DJ. Hearing aids for mild to moderate hearing loss in adults. Cochrane Database Syst Rev. 2017;9(9):CD012023. doi:10.1002/14651858.CD012023.pub2 28944461 PMC6483809

[47] Kitterick PT, Ferguson MA. Hearing aids and health-related quality of life in adults with hearing loss. JAMA. 2018;319(21):2225-2226. doi:10.1001/jama.2018.5567 29872841

[48] Peelle JE. Listening effort: how the cognitive consequences of acoustic challenge are reflected in brain and behavior. Ear Hear. 2018;39(2):204-214. doi:10.1097/AUD.0000000000000494 28938250 PMC5821557

[49] Hornsby BW, Naylor G, Bess FH. A taxonomy of fatigue concepts and their relation to hearing loss. Ear Hear. 2016;37(Suppl 1)(suppl 1):136S-144S. doi:10.1097/AUD.0000000000000289 27355763 PMC4930001

[50] Lin FR, Albert M. Hearing Loss and Dementia–Who Is Listening? Taylor & Francis; 2014. doi:10.1080/13607863.2014.915924 PMC4075051 24875093

[51] Pronk M, Deeg DJH, Smits C, . Prospective effects of hearing status on loneliness and depression in older persons: identification of subgroups. Int J Audiol. 2011;50(12):887-896. doi:10.3109/14992027.2011.599871 21929374

[52] Pichora-Fuller MK, Kramer SE, Eckert MA, . Hearing impairment and cognitive energy: the framework for understanding effortful listening (FUEL). Ear Hear. 2016;37(suppl 1):5S-27S. doi:10.1097/AUD.0000000000000312 27355771

[53] Sanchez VA, Arnold ML, Garcia Morales EE, . Effect of hearing intervention on communicative function: a secondary analysis of the ACHIEVE randomized controlled trial. J Am Geriatr Soc. Published online September 12, 2024. doi:10.1111/jgs.19185 PMC11637286 39266468

[54] Reed NS, Garcia-Morales EE, Myers C, . Prevalence of hearing loss and hearing aid use among US Medicare beneficiaries aged 71 years and older. JAMA Netw Open. 2023;6(7):e2326320. doi:10.1001/jamanetworkopen.2023.26320 37505496 PMC10383002

