# Supplementary material for: A Hearing Intervention and Health-Related Quality of Life in Older Adults: A Secondary Analysis of the ACHIEVE Randomized Clinical Trial
Source: JAMA Netw Open. 2024 Nov 21;7(11):e2446591. doi: 10.1001/jamanetworkopen.2024.46591 (PMC11582982; doi:10.1001/jamanetworkopen.2024.46591)
Supplement: Supplement 1. — Trial Protocol and Statistical Analysis Plan [file jamanetwopen-e2446591-s001.pdf]

Aging and Cognitive Health Evaluation in Elders (ACHIEVE) Randomized Trial

Protocol

ClinicalTrials.gov Number: NCT03243422

Co-Principal Investigators:

Frank R. Lin, MD PhD
Josef Coresh, MD PhD
Johns Hopkins University

Supported by:

The National Institute on Aging
Grant # R01AG055426

V.1.11 March 23, 2023

Changes from protocol version 1.10 to 1.11

This final protocol update reflects clarifications and modifications necessitated by COVID, as well as updates to match with the final Statistical Analysis Plan. These protocol updates were made prior to the final database lock or unblinding.

Minor changes (e.g., typographical and grammatical corrections, page numbering, section numbering and cross-references, formatting, and minor wording changes to improve clarity and readability) were made throughout the document. Substantive modifications are described here (full history of prior protocol amendments can be found in section 20):

**Affected section(s)**	**Brief description of change**	**Brief rationale for change**
Study Summary<br><br>2.1 Hearing loss and cognition<br><br>3 Overview of the ACHIEVE trial design<br><br>9.2 Successful Aging intervention	Expanded the name of the successful aging intervention to the successful aging health education control intervention.	The full name is the preferred name and is more informative about the type of intervention.
Study Summary	Updated Outcomes and Statistical Methodology sections	Updates reflect the May 9, 2022 ACHIEVE Statistical Analysis Plan.
1 Study objectives<br><br>7.5 Other pre-specified outcomes<br><br>7.7 – Table 3. Schedule of Evaluations<br><br>19.1 – Table 4. Schedule of Evaluations for Remote Data Collection	Updated to reflect that physical and social functioning, health-related quality of life, and physical activity are other pre-specified outcomes, not secondary outcomes.	Initially at study outset, we used the term “secondary outcomes” to refer to *all* outcomes other than the primary outcome of global cognitive change, but in finalizing the SAP, we distinguished between cognitive secondary outcomes and the other non-cognitive pre-specified outcomes. The protocol is now updated accordingly to ensure consistency with the final SAP (and the ClinicalTrials.gov record).
7 Data collection and measurements	Expanded the descriptions of several of instruments/measures.	These changes were made to provide more details on the instruments and how the outcomes are scored.
7.4.1 Demographics questionnaire	Clarified that the ACHIEVE demographics questionnaire is only completed by de novo participants.	The schedule of evaluations had previously footnoted that ARIC participants did not need to complete the demographics questionnaire.
7.5.4 RAND 36-Item Health Survey<br><br>7.6 Communication partners assessments	Corrected the protocol to reflect that the RAND 36-Item Health Survey was administered instead of the SF-36.	The freely available RAND version of the 36-item health survey was administered in ACHIEVE, rather than the proprietary SF-36. The items are the same between the two measures, but the scoring differs slightly.

7.7 – Table 3 Schedule of Evaluations		
7.7 – Table 3. Schedule of Evaluations	Added footnote that the blood draw for ApoE could be done during a follow-up visit.	In some instances, the blood draw was not able to be obtained at the baseline visit. As ApoE genotype is not time varying, the sample could be obtained at any study visit.
7.7 – Table 3. Schedule of Evaluations	Updated the footnotes previously noted as "Procedures that are intermittent based on protocol."	The old footnote has been split into several different footnotes that provide details about when or why the procedures are or are not completed, consistent with what was in the manuals of procedures for the study.
12 Statistical Considerations	Updated all sections to reflect what is in the May 9, 2022 Statistical Analysis Plan.	The COVID-19 global pandemic led to suspension of in-person research study visits from March 2020 to June 2021 in order to protect participants, staff, and the larger community from risk of infection from COVID-19, which necessitated implementing remote data collection procedures. During this period, participants completed the neurocognitive battery over the phone. Beginning in June 2021, in-person visits resumed, and some participants had delayed in-person Year 3 visits. The analytic approach was updated to reflect these changes in the availability and timing of neurocognitive data collection that were necessitated by the pandemic.
12.4 Assumptions analysis	Updated to detail the interim analysis of select parameters (rate of hearing intervention drop-in and drop-out, rate of attrition) used to estimate the original trial sample size. This interim analysis was conducted in June 2019 before closure of trial recruitment.	Changes made based on DSMB recommendations

Contents

Study Summary 7
1 Study objectives 10
2 Background and rationale 10
2.1 Hearing loss and cognition 10
2.2 Pilot research 11
3 Overview of the ACHIEVE trial design 12
4 Study population and eligibility 13
4.1 Inclusion criteria 13
4.2 Exclusion Criteria 14
5 Recruitment and retention 15
5.1 Integration within ARIC-NCS 15
5.2 Field centers 16
5.3 Recruitment strategies 18
5.4 Retention strategies 18
6 Participant rights and confidentiality 19
6.1 Informed consent 19
6.2 Participant confidentiality 20
7 Data collection and measurements 21
7.1 Audiometric assessment 21
7.1.1 Case history 21
7.1.2 Air conduction audiometry 21
7.1.3 Tympanometry 21
7.1.4 Bone conduction audiometry 21
7.1.5 Word Recognition in Quiet 21
7.1.6 Quick Speech in Noise (unaided) 22
7.2 Cognitive battery 22
7.2.1 Administration 22
7.2.2 Ensuring speech understanding test 22
7.2.3 Cognitive domains 23
7.2.4 Delayed word recall test (DWRT) 23
7.2.5 Digit Symbol Substitution test (DSST) 23
7.2.6 Incidental learning 23
7.2.7 Trail Making Test Part A (TMT A) 24
7.2.8 Trail Making Test Part B (TMT B) 24
7.2.9 Logical Memory I and II 24
7.2.10 Digit Span Backwards 24
7.2.11 Boston Naming test 25
7.2.12 Word Fluency 25
7.2.13 Animal Naming 25
7.3 Dementia and MCI assessments 25
7.3.1 Neurological interviews 26
7.3.2 Clinical Dementia Rating Scale (CDR) 27
7.3.3 Functional Assessment Questionnaire (FAQ) 27

7.3.4 Neuropsychiatric Inventory (NPI) 27
7.3.5 Characterization of participants who leave the study early 27
7.4 Inclusion/exclusion/covariates 28
7.4.1 Demographics questionnaire 28
7.4.2 Health history questionnaire 28
7.4.3 Neurologic History 28
7.4.4 Anthropometry 28
7.4.5 Seated blood pressure 29
7.4.6 Blood draw 29
7.4.7 Wide Range Achievement Test (WRAT) 29
7.4.8 Pure-tone audiometry (screening) 29
7.4.9 Vision screening 29
7.4.10 Mini-Mental State Exam (MMSE) 29
7.4.11 Activities of Daily Living (ADL) 30
7.4.12 COVID impact questionnaire 30
7.5 Other pre-specified outcomes 30
7.5.1 Center for Epidemiological Studies Depression and Hopelessness Scale (CES-D) 30
7.5.2 Baecke Physical Activity Questionnaire 31
7.5.3 Hearing Handicap Inventory for the Elderly – Screening Version 31
7.5.4 RAND 36-Item Health Survey 31
7.5.5 Social Network Index 31
7.5.6 UCLA Loneliness Scale 32
7.5.7 Accelerometry 32
7.5.8 Falls and mobility 32
7.5.9 Hospitalizations 32
7.5.10 Grip strength 32
7.5.11 Short Physical Performance Battery (SPPB) 33
7.5.12 Intervention Feedback Form 33
7.6 Communication partners assessments 33
7.7 Schedule of evaluations 33
8 Randomization 37
9 Interventions 37
9.1 Hearing intervention 37
9.2 Successful Aging intervention 39
10 Strategies to promote intervention adherence/compliance & minimize bias from unblinded study participants and staff 41
11 Safety monitoring 44
11.1 Data and safety monitoring board 44
11.2 Adverse events 44
12 Statistical considerations 47
12.1 Sample size 47
12.2 Analytic approach 48
12.3 Global cognitive function factor score 53

12.4 Assumptions analysis 53
13 Data management 54
14 Data handling and record keeping 54
15 Quality assurance and quality control 55
15.1 Fidelity of hearing intervention 55
15.2 Fidelity of successful aging intervention 56
15.3 Quality assurance of data entry 56
16 Study monitoring, auditing, and inspecting 57
17 Timeline 58
18 Organization of the study 59
18.1 Key roles of committees 59
19 Changes as a result of COVID-19 60
19.1 Remote visits 60
19.2 Modifications to neurocognitive assessment 62
19.2.1 Neurocognitive tests with no substantive changes 62
19.2.2 CERAD recall 62
19.2.3 Shortened Mini-Mental State Exam 62
19.2.4 Oral Trails A 62
19.2.5 Oral Trails B 62
19.3 Addition of interim check-in calls 62
20 History of protocol amendments 63
21 References 67
22 Appendix A: Delivery of the Other Intervention after Completing ACHIEVE Trial 71
22.1 Successful Aging intervention for participants originally randomized to the hearing intervention group 71
22.1.1 Overview 71
22.1.2 Schedule of activities 71
22.2 Hearing intervention for participants originally randomized to the Successful Aging intervention group 71
22.2.1 Overview 71
22.2.2 Blended telehealth hearing health care delivery model 71
22.2.3 Schedule of activities for delivery of the hearing intervention to SA participants after completing ACHIEVE 72
22.2.4 Procedures 72
23 Appendix B: Extended Follow-Up of Successful Aging Participants after Delivery of the Hearing Intervention 74
23.1 Background and rationale 74
23.2 Participants 74
23.3 Schedule of activities 75
23.4 Procedures 75
23.5 Safety 76

Study Summary

Title	Aging and Cognitive Health Evaluation in Elders (ACHIEVE) Randomized Trial
Short Title	ACHIEVE
Field Site(s)	• George W. Comstock Center, Johns Hopkins Bloomberg School of Public Health, Washington County, MD<br>• University of Mississippi Medical Center, Jackson, MS<br>• University of Minnesota, Minneapolis, MN<br>• Wake Forest University, Forsyth County, NC
Other Study Sites	• University of South Florida (hearing intervention unit)<br>• University of Pittsburgh (successful aging health education intervention unit)<br>• Johns Hopkins University (study governance and analysis unit)<br>• University of North Carolina, Chapel Hill, NC (Data Coordinating Center)
Design	Randomized, open-label trial comparing hearing rehabilitative intervention versus successful aging control intervention for rate of cognitive decline.
Sample Size and Population	The trial was designed to enroll approximately 850 participants, including participants from the ongoing Atherosclerosis Risk in Communities Neurocognitive Study (ARIC-NCS) and non-ARIC participants recruited from the community ("de novo"). Following the June 2019 interim analysis review, the Data and Safety Monitoring Board (DSMB) supported extending recruitment, and ACHIEVE received an administrative supplement from the National Institute on Aging (NIA) to extend recruitment and increase the sample size up to potentially 1,000 participants; 977participants were enrolled.
Objectives	To determine the effect of a best practice hearing rehabilitative intervention versus a successful aging health education control intervention on decline in global cognitive function (primary outcome measure), decline in cognitive domains, adjudicated incident dementia and mild cognitive impairment (MCI), physical and social functioning, health-related quality of life (HRQL), and physical activity in 70-84 year-old well-functioning and cognitively-normal adults with hearing loss.<br><br>To investigate whether hearing rehabilitative intervention alters established trajectories of cognitive decline in participants recruited from ARIC-NCS, and to investigate the effect of hearing rehabilitative intervention on cognitive decline in persons with Alzheimer's disease risk factors and biomarkers.

Main Inclusion/Exclusion Criteria	Community-dwelling adults aged 70-84 years with mild to moderate audiometric hearing loss, and free from substantial cognitive impairment at baseline.
Randomization and stratification	1:1 permuted block randomization, stratified by severity of hearing loss (mild, moderate), ARIC status (ARIC or non-ARIC participant), and field center. Qualifying spouse/partner pairs are randomized as a unit, stratified by ARIC status and field center.
Study Interventions	**Hearing intervention (HI):** Participants in the hearing intervention group are fitted with a hearing aid, instructed to be worn on a daily basis for study duration, and attend four 1-hour intervention sessions spaced over the 2-3 months post-randomization. Re-instruction in use of devices and hearing rehabilitative strategies will be provided every 6 months.<br>**Successful aging health education control intervention (SA):** The Successful Aging intervention consists of four 1-hour intervention sessions spaced over the 2-3 months post-randomization focusing on participant-selected topics from the 10 Keys™ to Healthy Aging program and upper body stretching. Additional sessions will be provided every 6 months.
Participant Duration	Approximately three years, with four intervention visits in the 2-3 months following baseline and follow-up visits every 6 months until the 3-year visit.
Outcomes	**Primary**: Cognitive decline, as measured by the change from baseline in the global cognitive function factor score.<br>**Secondary**: Time until a composite outcome of (1) adjudicated dementia or adjudicated MCI diagnosis, (2) a 3-point drop in the 30-item MMSE administered in-person, or (3) a 3-point drop in a factor score derived from the 10-item MMSE orientation subscale and 11-item Blessed scale administered over the phone and rescaled to be equivalent to the 30-item MMSE; also, decline in the cognitive domain factor scores for memory, executive function, and language derived from in-person assessments.

Statistical Methodology	Primary: Groups will be compared for the primary outcome using mixed effects models that account for the correlation among repeated measures as well as the correlation between spouses or cohabitating partners. If a linear trend appears reasonable, we will fit a model with a linear slope. If a nonlinear trend is observed, the model will be adapted to include time splines. Continuous time in years from the baseline will be the time scale. An interaction term between treatment assignment and time will be used to test if cognitive change differs by treatment assignment. Model fit will be assessed with residual plots and other statistics (Akaike Information Criterion, Bayesian Information Criterion, etc.). The primary analysis may include adjustments for baseline hearing loss, ARIC vs de novo status, field center, age, sex, education, and APOE ε4 alleles.<br><br>Missing cognitive factor scores among ACHIEVE participants will be generated utilizing multiple imputation by chained equations.<br><br>An independent DSMB will review safety outcomes on a regular basis. A formal interim analysis to evaluate for sample-size re-estimation was initially planned for when 66% of subjects completed the study. The formal interim analysis was cancelled at the recommendation of the DSMB and replaced with a sample size assumptions evaluation conducted in June 2019 prior to recruitment close to re-evaluate the parameter assumptions used to estimate the initial sample size.

1 Study objectives

The aims of the Aging and Cognition Health Evaluation in Elders Randomized Trial (ACHIEVE) are:

Primary aims

Aim 1 To determine the effect of hearing rehabilitative intervention versus a successful aging control intervention on decline in global cognitive function (primary outcome measure) in 70-84 year-old well-functioning and cognitively-normal older adults with hearing loss.

Aim 2 To determine the effects of hearing rehabilitative intervention versus a successful aging control intervention on secondary outcome measures of decline in cognitive domains (memory, executive function, and language); composite outcome consisting of adjudicated incident dementia, adjudicated MCI or a 3-point decline in the MMSE; and on other pre-specified outcomes related to physical and social functioning, HRQL, and physical activity.

Secondary Aims

1. To investigate whether hearing rehabilitative intervention alters established trajectories of cognitive decline in participants recruited from ARIC-NCS. We will leverage cognitive data gathered over the previous 30 years in ARIC to model prior cognitive change as well as prospective change after enrollment in ACHIEVE.

2. To investigate the effect of hearing rehabilitative intervention on cognitive decline in persons with Alzheimer's disease risk factors (ApoE ε4 allele) and biomarkers (neurodegeneration based on structural brain MRI; brain amyloid based on PET). Hearing loss may act as an additional "hit" on the brain in addition to AD and vascular processes. However, unlike these other processes, the effects of hearing loss may still be modifiable with hearing interventions in late life.

2 Background and rationale

2.1 Hearing loss and cognition

Novel approaches for reducing cognitive decline in older adults are needed given the aging of the population and the personal, socioeconomic, and public health implications of cognitive impairment and dementia in older adults. Epidemiologic data now strongly suggest that age-related peripheral hearing loss in older adults is independently associated with accelerated cognitive decline and incident dementia. Mechanistic pathways that could underlie this observed association include the effects of poor audition and distorted peripheral encoding of sound on cognitive load, brain structure, and/or reduced social engagement. These pathways may be amenable to comprehensive hearing rehabilitative treatment consisting of the use of hearing assistive technologies (hearing aids, other integrated hearing assistive devices) and rehabilitative training. To date, however, there has never been a randomized trial that has investigated whether hearing loss treatment could reduce cognitive and other functional

declines in older adults. The ACHIEVE study will be a randomized controlled trial nested within the Atherosclerosis Risk in Communities (ARIC) study. The initial target enrollment was 850 70-84 year-old cognitively normal older adults with hearing loss, who will be randomized 1:1 to the hearing intervention (hearing needs assessment, fitting of hearing devices, education/counseling) or successful aging health education control intervention (individual sessions with a health educator covering healthy aging topics)

2.2 Pilot research

We conducted a pilot study (ACHIEVE-P) from 2015-16 at the Washington County, MD ARIC field site. The objectives of ACHIEVE-P were to determine feasibility of study recruitment, randomization, and retention, assess compliance with study interventions, refine study protocols, and observe for an efficacy signal of the hearing intervention on early 6-month outcomes that may mediate downstream effects of hearing intervention on cognitive functioning. Participants (N=40) were randomized to either the hearing intervention or successful aging intervention in a 1:1 allocation ratio. Among the 40 randomized participants, 27 were from ARIC and 13 were recruited de novo from the community. Participants were followed until study completion (6 months), excluding one participant who died during follow-up for reasons unrelated to the study intervention.

The hearing intervention demonstrated a clear efficacy signal for early proximal outcomes of communication and social functioning. Estimated changes in standardized (z-score) outcomes were qualitatively different by intervention assignment for all measures, including perceived handicap due to hearing loss (as measured by the Hearing Handicap Inventory for the Elderly, $p<0.0001$), loneliness (as measured by the UCLA Loneliness Scale), number of contacts ($p=0.007$), and diversity of social network (as measured by the Cohen Social Network Index) and social, mental, and physical function (as measured by the Short Form (SF)-12). For all measures, estimates of change since baseline are suggestive of improvement or no change for the hearing intervention group, as compared to poorer function in the successful aging group (Table 1).

Table 1. 6-month change in standardized proximal outcomes.

Outcome	Hearing Intervention Mean (SD)	Successful Aging Mean (SD)
Perceived handicap due to HI*	-1.40 (0.96)	0.02 (0.68)
Loneliness*	-0.19 (0.87)	0.22 (0.94)
Social Network†		
Number of people	0.17 (0.65)	-0.42 (0.66)
Diversity	0.15 (1.25)	-0.12 (0.70)
Social Function†	0.00 (0.65)	-0.26 (0.91)
Mental Function†	0.26 (0.80)	-0.14 (0.60)
Physical Function†	0.11 (0.76)	-0.07 (0.40)

* Lower scores are better; † Higher scores are better.

<u>Cognitive performance by intervention assignment</u>

Estimated changes in standardized (z-score) cognitive domain scores over 6 months of follow-up were suggestive of improvement or no change for *all* participants (Table 2). The greatest estimated improvement in cognition was for participants who received the hearing intervention in the domain of memory. The mean change in memory for participants randomized to the hearing intervention was 0.48 ± 0.69 SD, as compared to 0.19 ± 0.66 SD for participants randomized to the successful aging intervention.

Table 2. 6-month change in standardized cognitive domain score, n =40

Cognitive Domain	Hearing Intervention	Successful Aging
	Mean (SD)	Mean (SD)
Memory	0.48 (0.69)	0.19 (0.66)
Language	0.05 (0.38)	0.00 (0.42)
Executive Function	0.03 (0.42)	0.17 (0.47)
Global Function	0.16 (0.42)	0.14 (0.39)

3 Overview of the ACHIEVE trial design

The study is a randomized controlled trial. Approximately 850 participants will be randomly assigned to one of two groups and followed for three years: hearing intervention with hearing aids or successful aging health education control intervention. Participants will be community-dwelling adults aged 70-84 years with audiometric hearing impairment defined as a four-frequency (0.5, 1, 2, 4 kHz) pure-tone average threshold in the better-hearing ear of ≥30 decibels (dB) and <70dB.

The ACHIEVE clinical trial will be nested within the infrastructure of the ongoing Atherosclerosis Risk in Communities Neurocognitive Study (ARIC-NCS) which provides an existing, well-characterized cohort of African-American and white participants who have been followed for nearly 30 years. Recruitment will be based at the four established ARIC field sites (Washington County, MD; Jackson, MS; Forsyth County, NC; Minneapolis Suburbs, MN). To fulfill the ACHIEVE sample size, non-ARIC "de novo" participants recruited from the communities surrounding the ARIC fields centers will also be concurrently enrolled. The recruitment ratio goal is 50% ARIC participants within each field center.

The hearing intervention consists of fitting with hearing aids and other hearing assistive technologies supplied by a single hearing technology manufacturer (Phonak) plus four 1-hour comprehensive, individualized hearing rehabilitation sessions spaced over the 2-3 months post-randomization designed to provide all of the active components of the intervention. Hearing aids are instructed to be worn on a daily basis for study duration. Audiologic outcomes (e.g., hearing aid data logging, real ear measures, speech in noise, etc.) to verify the best-practices hearing intervention are gathered during study intervention visits and semi-annually beginning at 6 months post-randomization. Participants are encouraged to specify an optional Communication Partner (CP) who is encouraged to attend the hearing intervention sessions.

The Successful Aging intervention will follow the protocol and materials developed for the 10 Keys™ to Healthy Aging program by the Center for Aging and Population Health Prevention Research Center at the University of Pittsburgh.[1] This interactive, dynamic program informs older adults about risk factors for diseases. Participants will meet individually with a health educator certified to administer the program every 2-3 weeks for a total of 4 visits over 2-3 months, and the session content will focus on a "Key". Each session will also include a 5-10 minute active upper body extremity stretching program as used in the Lifestyle Interventions and Independence for Elders (LIFE) study.[2] Participants are encouraged to bring a friend or family member with them to intervention visits (analogous to the communication partner for participants randomized to the hearing intervention). Participants will return for booster sessions semi-annually.

Participants will be followed for at least 3 years after randomization with annual assessments, and this study is designed to detect a 0.26 SD difference in rate of decline on domain-specific composite scores between the hearing intervention and control groups over 3 years of post-randomization follow-up with 90% power.

At the completion of the study, participants in the successful aging intervention may also choose to be fitted for a study-supplied hearing aid and provided hearing rehabilitation resource materials over 2-3 intervention sessions if they are interested. Participants in the hearing rehabilitative may also choose to meet with a health educator to cover topics in the 10 Keys™ to Healthy Aging program over 2-3 intervention sessions.

4 Study population and eligibility

The study population consists of adults aged 70-84 years free from substantial cognitive impairment at baseline. All eligible participants must have an adult-onset audiometric hearing loss as measured by a pure-tone average across 4 frequencies (0.5, 1, 2, 4 kHz) ≥ 30 dB HL in the better-hearing ear. Participants with hearing loss that is unlikely to benefit from amplification (e.g., poor word recognition in quiet) will be excluded from the study. Eligibility will be determined over a telephone screening and an in-person screening visit or based on audiometric and other data collected during ARIC-NCS study visits for existing ARIC-NCS participants. Confirmatory audiometric testing will be conducted at the baseline visit (performed on the same day or a separate day from the screening visit) by the study audiologist. Participants who initially qualify for the study based on screening (telephone screening, in-person screening, or screening based on ARIC-NCS data) but who do not meet study eligibility criteria based on data gathered at the baseline visit will be excluded from the study. Eligibility criteria are as follows:

4.1 Inclusion criteria

To be eligible for the study, participants must meet all of the following criteria:

- **Age 70-84 years.** Individuals aged 70-84 at the time of randomization are eligible for participation. This age range will allow recruitment of participants who are at risk for

cognitive decline but who may also be expected to survive for the duration of the trial. This age range is estimated to allow for potential participation of 61% of participants currently enrolled in the ARIC study.

- **Community-dwelling.**
- **Fluent English-speaker.**
- **Residency.** Participants must plan to reside in the local area for the study duration.
- **Audiometric hearing impairment.** Participants must have adult-onset hearing impairment with a four-frequency pure-tone average (0.5, 1, 2, 4 kHz) in the better-hearing ear of ≥30 dB HL (decibels hearing level) and <70 dB HL. This level of hearing impairment is the level at which individuals would be most likely to benefit from the use of conventional amplification devices such as hearing aids.
- **Word Recognition in Quiet score ≥60% in better ear.** A word recognition in quiet score <60% suggests hearing impairment that is too severe to benefit from conventional amplification devices such as hearing aids.
- **Mini-Mental State Exam (MMSE) score ≥23 for individuals with high-school degree or less; Mini-Mental State Exam (MMSE) score ≥25 for individuals with some college or more.** Participants must be at risk for cognitive decline in the range quantified well by neurocognitive testing, and so must be free from more substantial cognitive impairment at baseline.
- **Willingness to participate, be randomized, and adhere to the protocol.** Participants must be willing and able to consent to participate in the study, be willing to be randomized to either the Hearing intervention or to the Successful Aging intervention, and be willing to commit to adhere to the study protocol for the duration of the trial (3 years of a randomly assigned intervention).

4.2 Exclusion Criteria

Potential candidates for enrollment who meet one or more of the following criteria are excluded from participation in the study:

- **Self-reported disability in ≥2 Activities of Daily Living (ADLs)**
- **Any self-reported hearing aid use in the past year.** Trial participants will be randomized to hearing intervention or successful aging intervention and, therefore, participants cannot be receiving treatment for their hearing loss already.
- **ARIC participants only: Diagnosis of adjudicated dementia based on a previous ARIC visit or participant required a proxy to assist with completing informed consent and responding to questions at ARIC Visit 6 or 7.**
- **Vision impairment** (worse than 20/63 on MNREAD Acuity Chart). Participants who cannot see (with correction) well enough to complete the neurocognitive assessment are excluded.
- **Medical contraindication to use of hearing aids** (e.g., draining ear). Because hearing aids will be the primary device used in the hearing intervention, participants with medical contraindications to hearing aid use are excluded.
- **Permanent conductive hearing impairment** as determined by a difference in air audiometry and bone audiometry (“air-bone gap”) greater than 15 dB in 2 or more

contiguous frequencies in both ears. Because the impact of a conductive (versus a sensorineural) hearing loss on cognitive functioning may potentially differ and programming for hearing aids differs for conductive hearing loss, participants with permanent conductive hearing loss are excluded from the trial. Participants with an air-bone gap due to fluid in the ears or other resolvable medical issue may be enrolled in the trial following successful medical resolution of the cause of the air-bone gap (see Temporary Exclusion Criteria below).

- **Unwilling to wear hearing aids on a regular (i.e., daily or near daily) basis**
- No participants are excluded based on race or sex.

Temporary Exclusion Criteria

Participants determined to have a conductive hearing impairment as measured by a difference in air audiometry and bone audiometry (“air-bone gap”) greater than 15 dB in 2 or more contiguous frequencies in both ears are referred for medical follow-up. Should the cause of the air-bone gap be determined to be due to a temporary or treatable medical issue (e.g., fluid or wax in the ears), participants may be enrolled following resolution of the issue (i.e., air-bone gap). Participants with asymmetrical sensorineural hearing (≥20 dB at 2 adjacent frequencies or ≥10 dB at 3 adjacent frequencies) or with other symptoms/signs concerning for a retrocochlear etiology based on the assessment of the audiologist will also not be eligible to participate until a medical clearance/evaluation is obtained.

5 Recruitment and retention

The Operations Committee (see section 18.1) monitors recruitment and retention of participants.

5.1 Integration within ARIC-NCS

The ARIC study is a large, biracial prospective cohort study that enrolled 15,792 participants aged 45-64 years from four US communities in 1987-1989: Jackson, MS, Forsyth County, NC, Washington County, MD, and Minneapolis suburbs, MN. The Jackson cohort was entirely African-American, and the Forsyth County site was about 15% African-American, with all other participants being primarily white. ARIC participants have received multiple assessments of cardiovascular risk factors, measurement of microvascular and macrovascular markers, cognitive testing, PET amyloid, and brain MRI over the last 30 years. The ARIC study is well described with over 1700 papers published in peer-reviewed journals. Details of the overall ARIC study design have been published[3] and can also be found at https://sites.cscc.unc.edu/aric/. From 2011 to 2014, ARIC participants returned for Visit 5 (V5) as part of the ARIC Neurocognitive Study (ARIC-NCS) to evaluate midlife vascular risk factors as predictors of late-life cognitive impairment. Beginning in 2015, ARIC-NCS was jointly funded by NHLBI, NIA, NIDCD, and NINDS (Co-PI: Coresh/Mosley) to conduct ARIC-NCS Visit 6 (V6, 2016-17) and Visit 7 (V7, 2018-19) that will consist of a neurocognitive battery, audiometric testing, and physical functional assessments. The ACHIEVE trial will be nested within ARIC-NCS, and recruitment will occur directly during V7. As such, V6 and V7 data will be used to

directly screen and recruit ARIC participants for ACHIEVE in parallel with participants also recruited de novo from the community.

To maximize efficiency and minimize participant burden, overlapping assessments between the ACHIEVE baseline visit and ARIC-NCS Visit 7 will generally be collected only once and shared across studies. If ACHIEVE baseline is completed first, the remaining ARIC Visit 7 assessments will be completed at a later visit, but shared elements will be omitted from NCS since ACHIEVE baseline data without randomized treatment group will be shared with ARIC-NCS for approved uses. If ARIC Visit 7 is completed first, the ACHIEVE baseline visit must be completed within 2 months to share overlapping data; otherwise a full ACHIEVE baseline must be completed at least 6 months after ARIC Visit 7. Overlapping items are identified in the schedule of assessments and further described in the manual of procedures (MOP).

5.2 Field centers

Recruitment will be based at the four established ARIC field sites (Washington County, MD; Jackson, MS; Forsyth County, NC; Minneapolis suburbs, MN). Participants will be preferentially recruited from ARIC-NCS to increase efficiency through targeted recruitment and use of existing ARIC resources (previously collected data, etc.). Highly trained ARIC staff at the four field centers will be responsible for the recruitment of ACHIEVE participants. Conservative estimates based on recruitment rates during the ACHIEVE-P pilot study project that ~425 of the 850 ACHIEVE participants will be from the ARIC-NCS study.

George W. Comstock Center in Washington County, MD

The George W. Comstock Center is a dedicated research facility, affiliated with the Johns Hopkins Bloomberg School of Public Health, where Washington County residents were recruited and followed for ARIC. The ARIC study as well as a number of other NIH-funded studies are conducted in the Comstock research facility which houses 30 employees in approximately 10,000 square feet of space dedicated to community-based research with multiple examination rooms, conference rooms, and research space. Dr. Coresh is the director of this Center. The Comstock Center is located in Hagerstown, Washington County, Maryland, 75 miles from the Johns Hopkins University campus. The research center has handicap accessible entrances from the street, waiting rooms, examination rooms, interview rooms, phlebotomy and blood processing rooms with space for refrigerators and a freezer, lounge, conference rooms, file rooms, storage space, kitchens for preparation of snacks, and handicap accessible restrooms. The center has adjacent free parking. The Department of Epidemiology faculty and administrative staff oversee and advise on clinical and study conduct issues. The current project coordinator at the Center oversees space and staff sharing issues.

The University of Mississippi Medical Center in Jackson, MS

The University of Mississippi Medical Center (UMMC) has a long history of excellence in cardiovascular and neuro-epidemiologic research including recruitment and retention of African American participants for several large NIH-funded population-based studies (such as ARIC). The site has recruited and followed City residents for ARIC. The Memory Impairment and Neurodegenerative Dementia (MIND) Center was launched in 2010 at UMMC as a new initiative

to elucidate the mechanisms and risk factors for brain aging and age-related diseases such as Alzheimer's. To enhance synergy across projects, cohort and clinical studies focused on aging and neurologic endpoints were brought together under the MIND Center umbrella. Dr. Mosley is the principal investigator for the ARIC Jackson Field Center at UMMC and the director of the MIND Center. Housed in the MIND Center, the ARIC Field Center is conveniently located on UMMC's main campus. The ARIC Field Center includes 18,000 square feet of space including office, interview, and examination rooms. Facilities consist of a reception area and waiting room, multiple interview/procedure rooms, nurses' work station and office space, kitchen, storage areas with locking file cabinets, and phlebotomy and sample processing area with multiple -70°C freezers. Parking is conveniently located immediately behind the building. A dedicated van is available to shuttle study participants to diagnostic facilitates on campus (e.g., MR imaging) or to conduct assessments in the participant's home, if needed. In 2017, the MIND Center (and the ARIC Field Center) will move to a new state-of-the-art research building located on UMMC's main campus (also, with convenient parking). The ARIC clinic space will be outfitted with new state-of-the-art equipment and has been ergonomically designed specifically to enhance research with older participants (e.g., equipped with high/low powered exam tables to facilitate transfer on/off the table, etc.). The ACHIEVE trial at UMMC will be conducted in this new space.

University of Minnesota, Minneapolis, MN

The ARIC Field Site in Minneapolis, located at the Epidemiology Clinical Research Center at the University of Minnesota has recruited and followed residents of selected Minneapolis suburbs for ARIC. Dr. Pankow is the site principal investigator for the ACHIEVE study. The Epidemiology Clinical Research Center (ECRC) is located one block away from the offices of the Division of Epidemiology and Community Health, about 0.5 miles from the University of Minnesota Hospital on the Minneapolis campus, and is readily accessible from all parts of the Twin Cities metropolitan area (within three blocks from two major interstate highways). The ECRC occupies one floor (17,758 square feet) of a two-story building and includes reception area, offices for staff, examination rooms, interview rooms, ultrasound, phlebotomy and blood processing rooms, freezer room, lounge, conference rooms, and storage space. The building meets current regulations for handicapped accessibility and has 100 free adjacent parking spaces. The site was established in February 2000 and is currently providing space and infrastructure for 25 research projects including multi-center epidemiologic studies and clinical trials.

Wake Forest University, Forsyth County, NC

The Forsyth County ARIC Field Site is located at Wake Forest University. Dr. Hayden is the site principal investigator for the ACHIEVE study. The Public Health Research Center (PHRC) is the site for clinic examinations for County residents for ARIC and is located in the Piedmont Plaza I building on the Wake Forest University Baptist Medical Center campus. The PHRC provides investigators within the Division of Public Health Sciences with facilities and staff to perform multi-center clinical trials and observational research. The PHRC is easily accessible to all study participants as it is located on the ground floor of the Piedmont Plaza I Building, within a half mile of the main hospital, and is handicap accessible. With over 5,600 square feet of space, the PHRC has 18 rooms, a laboratory, a large waiting area, adequate parking that is free of charge,

and many additional amenities to enhance the research experience of our participants. This clinic is equipped to handle all examinations and tests associated with the ACHIEVE clinical trial.

5.3 Recruitment strategies

Each field site will implement a variety of strategies to achieve their recruitment targets with ACHIEVE participants being recruited both from ARIC-NCS and de novo from the surrounding communities. Recruitment of ARIC participants will occur concurrently with ARIC-NCS Visit 7 in close collaboration with the ARIC-NCS steering committee and as described in section 5.1. Recruitment of de novo participants will be from the surrounding ARIC field site communities. ACHIEVE field site PIs and field site staff have overseen recruitment of older adults for other epidemiologic clinical studies and will employ site-specific strategies that have demonstrated prior success with recruitment of older adults. Such strategies include: utilizing established research registries, targeted advertisements in aging-related publications/radio, established field site relationships with local churches/retirement centers, mass mailings, etc.

5.4 Retention strategies

In order to maximize adherence, at the time of screening, only those participants who are determined by study personnel to fully understand the commitments of the study and are likely to follow the study protocol including regular use or not (as randomized) of a hearing aid are enrolled. Enrolling dedicated participants from those still attending ARIC examinations after nearly 30 years is likely to maximize study retention.

The following procedures are implemented in order to enhance retention:

- When scheduling the clinic visit, participants will be asked about:
    - Preferred time and date of examination
    - How participants prefer to get to the clinic visit
    - Need for assistance getting to or moving around the clinic
    - Existence of any medical conditions (e.g., diabetes, dietary restrictions) which might affect the examination and/or type of snack provided.
- To enhance response following the scheduling telephone call by ACHIEVE staff, a packet will be mailed to the participant prior to the scheduled appointment. This pre-appointment packet confirms the examination date and time and reviews the preparation procedures. Prior to the examination a reminder call will also be made to the participant.
- Free parking is provided to all participants. Participants will be reimbursed for travel costs, or transportation will be covered for a participant if he/she is not able to drive and/or obtain a ride to attend a study visit.
- Participant study incentives will include study-related items (pens, bags, mailed holiday cards) and/or modest payments (~$20) for participating in each study visit. Individual field sites will determine the participant incentives to provide based on their previous experience and knowledge of their participants and community.
- Participants will be contacted by telephone to reschedule the appointment if eligible participants fail to arrive for a scheduled appointment or cancel their appointments.

- Home visits and telephone visits by study staff will be conducted as necessary when participants may be physically unable to come to the field site (e.g., from illness or injury). These visits will be informed by the experiences of study staff in performing such visits in ARIC-NCS Visits 5 and 6.
- Participants randomized to the hearing intervention and successful aging intervention group will also be informed (and reminded throughout their study participation) that they will have the option of receiving the other intervention upon their completion of the study.
- Each no-show case will be individually reviewed by the interviewer and when necessary by the supervisor. Efforts to engage the participant will include a combination of telephone contacts, letters, and the possibility of offering an abbreviated exam. Field site staff, in consultation with the study coordinator and/or field site PI and/or quality control committee, will determine how long to continue contact efforts. A participant will only be considered "withdrawn" if he/she explicitly requests to withdraw from the study. Participants are free to refuse or re-enter the study protocol after refusal at any time.

6 Participant rights and confidentiality

6.1 Informed consent

A signed consent form is obtained from each participant. The consent form describes the purpose of the study, the procedures to be followed, and the risks and benefits of participation. The purpose of the informed consent form is:

- To inform the prospective participant as much and as accurately as possible about:
    - The procedures involved in the study
    - What is expected of participants who consent to enroll
    - What the study can and cannot provide to the participant
    - What are the reasonable risks and benefits
    - What are the alternatives to participation
- To document the participant's consent to participate in screening, and all of the respective procedures involved.
- To provide a prospective participant with a legal document summarizing the study and his or her rights as a study participant.
- To provide the participant with ongoing explanations and continuing information that help the participant decide whether to begin or continue in the research study.

Reobtaining consent in the event of cognitive impairment

- Although those with dementia will not be enrolled at baseline, given the age of the cohort some participants may develop cognitive impairment over the course of the study. Those determined to have significantly reduced cognitive capacity during the trial and who wish to remain in the study will be re-consented with consent from a designated proxy to continue to participate in the study.

- If re-consent with a proxy is required, the re-consent may occur in person or remotely. Remote consent would be obtained by mailing two copies of the consent form to the proxy, having trained study personnel review the consent form with the proxy and participant over the telephone (answering questions and ensuring understanding), and having the proxy and participant sign one copy of the form and mail it back. Upon receipt, the study personnel who obtained consent would sign the form and document the consent process in a note in the participant's chart.
- Classifying decision-making capacity is challenging and often task specific. Given the minimal risk associated with the study procedures, relatively conservative criteria will be used to trigger re-consent with a proxy. These include: (1) a diagnosis of dementia based on cognitive testing at any of the ACHIEVE exams or (2) judgment of our trained staff at the time of an exam. These criteria adhere to the published recommendations of the Alzheimer's Association (Alzheimer Disease and Associated Disorders 2004; 18:171-175).

6.2 Participant confidentiality

Data from the ACHIEVE study are used only in aggregate, and no identifying characteristics of individuals will be published or presented. Results of select testing (weight, body mass index, and blood pressure) are given to participants, and they can choose to share those data with their health care provider. Alert values related to blood pressure or depression results are sent to participants' private physicians, if participants agree, and are also sent to the participant. Information, including results of testing to be shared with a participant's primary care physician, is not released without written permission of the participant, except as necessary for monitoring by IRB.

Confidentiality of data is maintained by using research identification numbers that uniquely identify each individual. This study utilizes safeguards established as part of the parent study (ARIC) to ensure the security and privacy of participants' study records. Research records are kept in locked file cabinets within locked rooms at the study site. Only selected study personnel will have access to participants' study records on a need-to-know basis. Data are stored on password-protected computers with regularly updated virus software. Identifying information is only kept in the files where it is necessary for the conduct of the study and linkage to other files. In analysis files, only study IDs are used to identify participants.

In compliance with the Health Insurance Portability and Accountability Act (HIPAA) and the Standards for Privacy of Individually Identifiable Health Information of the Department of Health and Human Services, ACHIEVE accesses personal health information and medical records only after receiving signed informed consent. Participants' medical records that are obtained for review and abstraction are kept in a locked cabinet that is separate from other file cabinets. Only selected study personnel have access to these files.

7 Data collection and measurements

7.1 Audiometric assessment

The audiometric diagnostic battery will be conducted by a trained audiologist for all participants, regardless of treatment assignment. The diagnostic battery is based on current American Academy of Audiology guidelines.[4] Cerumen management by the study audiologist will be performed as needed. Participants with more severe cerumen impactions that cannot be easily cleared by the study audiologist will be provided with over-the-counter cerumenolytic ear drops (e.g., Debrox, Murine) and advised to follow-up with their primary care provider or an otolaryngologist.

All audiometric testing will occur in a sound-attenuated booth. The WhisperRoom (www.whisperroom.com/) was chosen due to its small footprint and lower weight compared to traditional metal sound booths.

7.1.1 Case history

Audiological history including history of noise exposure, prior hearing aid use, etc.

7.1.2 Air conduction audiometry

Behavioral measurement of minimally perceptible tones tested across the frequencies most important for speech communication delivered through headphones.

7.1.3 Tympanometry

Objective measurement that determines integrity of the tympanic membrane/ossicles and assists in determination of a sensorineural hearing loss free of middle ear problems.

7.1.4 Bone conduction audiometry

Behavioral measurement of minimally perceptible tones tested across the frequencies most important for speech communication delivered through a bone oscillator, which allows determination of a sensorineural hearing loss free of middle ear problems.

7.1.5 Word Recognition in Quiet

Assesses the participant's ability to repeat back monosyllabic words presented at a comfortable listening level, scored as percent correct, to confirm that the participant's speech perception abilities are consistent with a hearing loss that can be helped through traditional hearing aid intervention.[5]

7.1.6 Quick Speech in Noise (unaided)

The QuickSIN is a speech-in-noise test that measures the signal-to-noise ratio (SNR) necessary for a listener to correctly identify 50% of key words on sentences presented in a babble background noise. A listener's abilities to understand speech in noisy backgrounds cannot be predicted by the audiogram, and this measure provides a tool to counsel the patient on realistic expectations for success with hearing aids and guides intervention decisions regarding hearing assistive technology (e.g., remote FM microphone). [Development of the test article;[6] Validation article[7]]

7.2 Cognitive battery

7.2.1 Administration

To assess cognitive decline and characterize dementia and Mild Cognitive Impairment (MCI), an efficient but comprehensive neuropsychological assessment will be administered by trained and certified examiners. The battery of cognitive measures (described below) are well-validated, standardized instruments that are widely used in clinical and epidemiologic studies of cognitive function and dementia, including ARIC-NCS, and include most of the measures recommended in the Uniform Data Set (UDS) implemented in 2005 across all National Institute on Aging-sponsored Alzheimer's Disease Centers.

A trained examiner administers the cognitive function tests in a fixed order, one right after the other, during a single session in a quiet room. To ensure that the participant understands each task, test instructions are presented verbally and visually (e.g., with a practice template or written instructions). The measures in the cognitive battery are briefly described below. Note that the MMSE (screener and secondary outcome) and WRAT (covariate) are also administered during the cognitive assessment and are described in sections 7.4.10 and 7.4.7, respectively.

7.2.2 Ensuring speech understanding test

Prior to performing the neurocognitive assessment, a brief test will be conducted to determine whether or not the participant can adequately hear the examiner. In this test, five sentences are read aloud to the participant by the psychometrist, and the participant is asked to repeat back the sentence. Participants are scored on the number of target words repeated back correctly (3 target words/sentence). Steps on how to proceed with neurocognitive testing if audibility is not established are outlined in the MOP.

The ACHIEVE Steering Committee developed this protocol to guard against poor speech understanding from hearing loss directly confounding administration of neurocognitive tests with auditory stimuli. This protocol was developed in collaboration with ACHIEVE Co-I's with expertise in neuropsychology (Albert, Mosley, Knopman, Rebok) and audiology (Chisolm, Eddins) and was successfully pilot tested in the ACHIEVE pilot study.

7.2.3 Cognitive domains

The ACHIEVE study will utilize the neurocognitive battery that was previously administered in ARIC-NCS[8] (individual tests that constitute the battery are described in sections 7.2.4 – 7.2.13). Global cognitive function and three cognitive domains are derived from factor scores[9] (see Section 12.3). These domains include:

- Memory
- Executive function
- Language

7.2.4 Delayed word recall test (DWRT)

The DWRT is a measure of verbal memory that requires the participant to recall a list of 10 common nouns following a short delay. The participant is presented with a stimulus card for each of 10 words. The examiner reads each word aloud, and asks the participant to repeat the word and use it in a sentence. This procedure is repeated, providing two exposures to the words. Following an approximate 5-minute delay, during which the (non-verbal) digit symbol substitution test (DSST) is given, the participant is asked to recall as many words as possible. Scores range from 0 to 10 words recalled.

7.2.5 Digit Symbol Substitution test (DSST)

The DSST is a measure of psychomotor speed and sustained attention. Besides its own value, the DSST also serves as a non-verbal distracter task, interposed between learning and recall for the DWRT above. The participant is asked to translate numbers (1-9) to symbols using a key provided at the top of the test form. The participant is provided with a pencil (without an eraser). Instructions are provided in a deliberate and slow pace. One point is given for each correctly drawn symbol completed within the 90-second time limit. Scores range from 0-93.

7.2.6 Incidental learning

The Incidental Learning Test was adapted from the Wechsler Adult Intelligence Scale, Revised as a Neuropsychological Instrument (WAIS-R NI) and provides a non-verbal measure of recent memory. Following the DSST, the participant is presented with the Incidental Learning Template. The participant is asked to write down as many of the DSST symbols as he/she can remember, in any order. Next, the participant is asked to write down the number that was paired with each of the symbols from the DSST. Two scores are yielded: 1) Free Recall: total number of symbols recalled, regardless of pairing and 2) Pairing: number of correct symbols correctly paired with corresponding numbers. Scores for each range from 0-9.

7.2.7 Trail Making Test Part A (TMT A)

The TMT A is a timed task in which participants connect numbers in sequence as quickly as possible. TMT measures attention, sequencing, mental flexibility, and visual search and motor function. In TMT A, the participant is asked to draw a line and connect a series of numbers (from 1-25) as quickly as possible. Prior to the test part, the participant is given a sample test to demonstrate the task. The score for TMT A is the number of seconds required to complete the task. A maximum of 240 seconds (4 minutes) and 5 errors is allowed.

7.2.8 Trail Making Test Part B (TMT B)

The TMT Part B is a timed task in which participants connect letters and numbers in sequence as quickly as possible. TMT measures attention, sequencing, mental flexibility, and visual search and motor function. In TMT B, the participant is asked to draw a line and connect a series of numbers and letters, alternating between a given number and letter (e.g., 1 to A, A to 2, 2 to B, B to 3, etc.) as quickly as possible. Prior to the test part, the participant is given a sample test to demonstrate the task. The score for TMT B is the number of seconds required to complete the task. A maximum of 240 seconds (4 minutes) and 5 errors is allowed.

7.2.9 Logical Memory I and II

This test, part of the Wechsler Memory Scale-Revised version, provides a measure of immediate and delayed verbal recall for the number of ideas presented in two stories, which are read to the participant. Two stories are read to the participant, each at a slow and deliberate pace. After each story is presented, the participant is asked to recall as much of the story as possible. The Logical Memory I score provides a measure of immediate recall and is calculated as the average number of ideas recalled from Story A and B. Each story contains 25 scoring units and the maximum score is 25.

An approximate 20-minute delay follows, during which the remaining (non-memory) tests are administered. Following the delay period, the participant is again asked to recall the stories. The Logical Memory II score provides a measure of delayed recall and is calculated as the average number of story elements recalled from Story A and B. As each story contains 25 scoring units and the maximum score is 25.

7.2.10 Digit Span Backwards

Digit Span Backwards is part of the Wechsler Memory Scale-Revised and provides a measure of attention and working memory. The participant is read a series of numbers progressively increasing in length from two to eight digits. After the numbers are read, the participant is asked to repeat the numbers in the reverse order. Two trials at each digit length are performed (i.e., 2

trials with 2 digits, 2 trials with 3 digits, etc.). The test is discontinued after two consecutive errors of the same length item. Scores range from 0-12.

7.2.11 Boston Naming test

The Boston Naming Test assesses visual naming ability using black-and-white drawings of common objects. For this study, the 30-item version used by the National Alzheimer's Coordinating Centers Uniform Data Set will be used. The participant is presented with a series of line drawings of objects and asked to name each object. The items become progressively more difficult based on their frequency of occurrence in the English language. A total score is calculated as the number of spontaneously produced correct responses. Scores may range from 0-30.

7.2.12 Word Fluency

The Word Fluency Test is a measure of verbal functioning. In this task, the participant is asked to produce as many words as possible that begin with the letters F, A, and S within a time limit of 60 seconds for each letter, avoiding proper nouns, variations, plurals, and repetitions. The score is the total number of admissible words produced across letters.

7.2.13 Animal Naming

Animal Naming is a measure of category fluency (semantic association). Category fluency, and specifically animal naming, is part of the Boston Diagnostic Aphasia Examination, the Stanford-Binet test, and the CERAD. The participant is asked to name as many different animals as possible within a 60-second time limit. The score is given as the sum of all admissible names.

7.3 Dementia and MCI assessments

A syndromic diagnosis of MCI and dementia will be determined and used as a secondary outcome. Current criteria for MCI[10] and dementia,[11] which were in part established by investigators for this project, are now well-established and have been employed successfully in ARIC-NCS. Details of the diagnostic procedures and normative data have been published and are enumerated in the MOP. Briefly, MCI and dementia syndromic diagnoses are determined by a panel of clinicians and neuropsychologists taking into account performance on the neuropsychological battery (test scores are compared to age, education, and race-specific norms), cognitive decline across study visits, and subject and informant interviews regarding cognitive functional status. Based on these elements, all examined participants have either a computer-algorithmic classification or an expert-adjudicated classification. The computer algorithm, also developed by the expert panel for ARIC-NCS, has been used successfully to enhance uniformity in applying the diagnostic criteria. In ARIC-NCS, the computer algorithm-

reviewer agreement was high: 99% for normal, 94% for MCI, and 95% for dementia, suggesting that the algorithm accords well with clinical judgment.

An MCI diagnosis is assigned in persons without dementia who meet all 3 criteria below:

1. Cognitive decline in general cognitive performance, and
2. At least one neuropsychological cognitive domain Z score < -1.5 Z, and
3. Either Functional Activities Questionnaire (FAQ) score or Clinical Dementia Rating Scale (CDR) Sum of Boxes, not both, may meet threshold for impairment (these measures are described below in section 7.3.2 and 7.3.3).

A dementia diagnosis is assigned by either:

A) A low MMSE score (<21 for Caucasians or <19 for African-Americans), even in the absence of more complete cognitive testing or

B) Meeting all three of the following criteria:

1. Cognitive decline in general cognitive performance, and
2. At least one neuropsychological cognitive domain scores < -1.5 Z, and
3. CDR Sum of Boxes meets threshold for impairment and FAQ score may meet threshold for impairment.

Participants failing to meet criteria for MCI or dementia are classified “normal”.

The informant interviews noted above are conducted with a knowledgeable informant at every exam where the participants meet a priori criteria for poor cognitive performance and who have significant cognitive decline from prior exams. These measures are no longer collected once criteria for dementia are met. Participants who leave the study early will have procedures for ascertainment of dementia detailed in Section 7.3.5.

7.3.1 Neurological interviews

The neurologic interviews include the Clinical Dementia Rating Scale (CDR) and the Neuropsychiatric Inventory (NPI). In addition, the Functional Activities Questionnaire (FAQ) is used in determining a participant’s level of daily functioning, but does not have a dedicated interview or form; rather, all FAQ items are embedded within the CDR interview. Each of the measures are well-validated, standardized instruments that have been widely used in both clinical and epidemiologic studies of dementia.

7.3.2 Clinical Dementia Rating Scale (CDR)

The CDR gives important information about daily functioning, and is a required element in the determination as to whether an individual is demented or has MCI, or is normal. The CDR includes the CDR Participant (CDP), and the CDR Informant (CDI), and the CDR Summary (CDS).

The CDP (the portion of the CDR administered to the participant) is administered to all participants. The CDP form is administered by a certified staff member to the participant while the participant is seated, and requires no equipment for administration.

The CDI is administered by a certified staff member with a knowledgeable informant and can be administered in the clinic, if the informant is available, or may be collected by phone.

After completion of these two components (and not in the presence of the participant or informant), a trained staff member will score the CDR (on the CDS form) based on the responses to the questions on both the CDP and CDI. The CDR scores range from 0 (normal) to 3 (severe impairment) for each of the following 6 areas: memory, orientation, judgment and problem solving, community affairs, home and hobbies, and personal care. The CDR Sum of Boxes sums over the 6 areas where each area score ranges from a 0 (normal function), 1 (has difficulty, but does by self), 2 (requires assistance), to 3 (dependent). A CDR Sum of Boxes >3 indicates impairment.

7.3.3 Functional Assessment Questionnaire (FAQ)

There are 9 items from the informant interview of the CDR which are also FAQ questions (there are 10 FAQ questions; one CDR question encompasses two FAQ questions). The FAQ score is calculated by summing the 9 individual items (1 item is added twice). Scores per item range from a 0 (normal function), 1 (has difficulty, but does by self), 2 (requires assistance), to 3 (dependent). An FAQ score >5 indicates impairment.

7.3.4 Neuropsychiatric Inventory (NPI)

The NPI consists of questions relating to personality and behavioral changes commonly seen in dementia. This scale is completed with a knowledgeable informant after the CDI, either in clinic or by telephone.

7.3.5 Characterization of participants who leave the study early

A final interview will be attempted with all participants who are either unable (e.g., due to illness) or unwilling to complete the study. Trained interviewers will collect information by telephone regarding the reason for withdrawal and offer to schedule a home visit for an abbreviated final

exam. In the absence of a final home exam, because dropouts may be more likely to have dementia, we will attempt to ascertain dementia status using a telephone-based assessment with the participant or informant interview conducted with a knowledgeable informant.

In the case where the participant is alive and able to communicate by phone, we will administer the Six Item Screener (SIS). The SIS is a short instrument developed to identify cognitive impairment in older adults. In the case where the participant has died or is otherwise unable to communicate by phone, dementia status may be characterized by informant interview via telephone using the AD8--a brief instrument, derived from the Clinical Dementia Rating Scale, developed to discriminate between normal aging and dementia. Notably, these procedures parallel ARIC's ongoing dementia surveillance methods. For ARIC participants, additional sources of information may also be used to complete ascertainment of dementia cases (e.g., discharge codes from hospitalizations, CMS, and ICD codes on death certificates).

7.4 Inclusion/exclusion/covariates

7.4.1 Demographics questionnaire

This is an interviewer-administered questionnaire given to de novo participants to gather age, sex, employment status, race, ethnicity, education, and marital status.

7.4.2 Health history questionnaire

This is an interviewer-administered questionnaire given to each participant, which documents a number of chronic diseases or conditions (e.g., hypertension, diabetes, stroke, Parkinson's disease, osteoporosis, among others). The collection of a medical history on each participant permits the assessment of overall health.

7.4.3 Neurologic History

This questionnaire includes items about past neurologic diagnoses and treatments. This information will be used as possible covariates and by MCI/dementia adjudicators.

7.4.4 Anthropometry

Participant height and weight will be measured using standardized study protocols from the parent study (ARIC). These are detailed in the MOP. Anthropometric measures include height, weight, waist and hip circumference, and body fat. These measures may be used to assess the risk of disease.

7.4.5 Seated blood pressure

Seated systolic and diastolic blood pressure will be measured using standardized study protocols from the parent study (ARIC). These are detailed in the MOP.

7.4.6 Blood draw

A blood draw for ApoE ε4 genotyping in non-ARIC participants in ACHIEVE will be conducted at baseline and done using standardized study protocols. ARIC participants have previously had ApoE genotyping performed.

7.4.7 Wide Range Achievement Test (WRAT)

The WRAT is a widely used measure of academic achievement and commonly used to provide an estimate of premorbid functioning in adults with neurological conditions. In addition to education level, scores on the WRAT Reading subtest will be taken into account by Dementia/MCI reviewers when interpreting neuropsychological tests results. Following brief instructions, the participant is handed a test card and asked to read the words on the card aloud. Responses are recorded on the paper form. The test is discontinued following 5 consecutive errors. Scores range from 0-70.

7.4.8 Pure-tone audiometry (screening)

Air conduction audiometry is conducted to see if the participant meets the audiometric inclusion criteria. Confirmatory audiologic testing performed by an audiologist is conducted during the baseline assessment.

7.4.9 Vision screening

This test is to determine the smallest print the participant can read. The MNREAD Acuity Chart is used as the vision screener. Full details can be found in the MOP.

7.4.10 Mini-Mental State Exam (MMSE)

The MMSE was developed as a brief, standardized instrument for screening a limited number of cognitive functions.[12] We will use the MMSE to exclude those with significant cognitive impairment at baseline and to track cognitive decline and dementia during study follow-up. The MMSE is administered by interview. A detailed script is provided for each item and task. Scores range from 0-30.

7.4.11 Activities of Daily Living (ADL)

Assessed by an interviewer-administered questionnaire, ADLs assess a person's ability to perform basic activities of daily living (e.g., getting in/out of bed or chairs, bathing, dressing, eating, toileting).

7.4.12 COVID impact questionnaire

Added in January 2021, this brief questionnaire asks questions about how the coronavirus pandemic, or COVID-19, have affected the participant.

7.5 Other pre-specified outcomes

7.5.1 Center for Epidemiological Studies Depression and Hopelessness Scale (CES-D)

Depressive symptoms have been linked to a number of important health outcomes including cardiovascular disease risk factors, coronary heart disease morbidity and mortality, cognitive functioning, and MCI/dementia. In ACHIEVE, depressive symptoms will be assessed using the Center for Epidemiologic Studies Depression Scale (CES-D) Short Form. The CES-D Short Form is derived from the original 20-item CES-D.[13] In addition to a reduced administration time and clearer response options (relative to the 20-item version), the Short Form is highly correlated with the original (r > .94), has a high internal consistency, retains the same factor structure as the original, and has a similar positive predictive value as a screening tool for identifying clinical depression. It takes approximately 3 minutes to complete this 12-item questionnaire. The questionnaire is administered by interview. As a scale for depression, responses must be provided by the participant, not a proxy. Because of the sensitive nature of some of the questions, interviewers must take care to ask questions and record responses in a sensitive and non-judgmental manner. Most of the questions are self-explanatory; however, if the respondent is unclear, the interviewer will repeat the question and use general phrases, such as: "Answer as best you can, based on how you have felt over the past week." Interviewers should not lead participants to an answer but remain neutral. Participants are asked to rate each item on a 3-point scale (scored 0 to 2) on the basis of "how often you have felt this way during the past week." Response categories are:

- Hardly ever or never (scored as 0)

- Some of the time (scored as 1)

- Much or most of the time of (scored as 2)

Positively worded items are reverse-coded, and a total score is calculated as the sum of the responses to questions 1-11. Total scores range from 0 to 22, with higher scores indicating greater expression of depression. The 12th item is a rating of hopelessness that is not included in the total score.

7.5.2 Baecke Physical Activity Questionnaire

This is an interviewer-administered questionnaire[14] given to each participant that measures the frequency and duration of self-reported activities, which is currently used in ARIC. This questionnaire asks about habitual physical activities including sport-related activities during leisure time, non-sport related physical activity during leisure time, and television viewing. Composite scores of sport during leisure time and leisure time excluding sport are generated as continuous measures each ranging from 1 to 5.

7.5.3 Hearing Handicap Inventory for the Elderly – Screening Version

This is an interviewer-administered questionnaire given to each participant to gather data on the perception of the impact of hearing loss.[15] This questionnaire assesses the social and emotional components of perceived hearing impairment such as embarrassment, and limits on personal and social life.

7.5.4 RAND 36-Item Health Survey

The RAND-36 Health Survey is an interviewer-administered scale that assesses health-related quality of life (HRQL).[16] It is a generic measure, as opposed to one that targets a specific age, disease, or treatment group. Physical health and mental health component scores are each derived using factor analysis, with mean of 50 and standard deviation (SD) of 10 (range 0 to 100, with higher scores indicating better health).

7.5.5 Social Network Index

The Cohen Social Network Index (SNI) is an interviewer-administered questionnaire given to each participant to gather social network data.[17] The Social Network Index evaluates two outcome variables: (1) Social Network Diversity – Number of social roles in which the participant had regular contact with for at least once every 2 weeks, and (2) People in Social Network – Total number of people with whom the participant had regular contact (at least once every 2 weeks). Examples of items include “How many other relatives (other than your spouse, parents & children) do you feel close to?”, “Do you attend any classes (school, university, technical training, or adult education) on a regular basis?”, “Are you currently involved in regular volunteer work?”, among others.

7.5.6 UCLA Loneliness Scale

This is an interviewer-administered questionnaire given to each participant to gather loneliness data.[18] This 20-item questionnaire measures subjective ratings of social isolation and loneliness (e.g., lacking companionship, feeling left out, and isolated from others, among others) using a 4-point Likert scale. Positively worded items are reverse-coded, and an average score ranging from 1 to 4 is generated, with higher scores indicating greater expression of loneliness.

7.5.7 Accelerometry

Physical activity is objectively assessed using the Actigraph Link accelerometer, an FDA approved, triaxial, water-resistant, wrist-worn device that can be worn 24 hours a day, continuously measuring intensity, duration, and frequency of physical activity. Participants will be fitted with the device during their clinic visit and asked to continue wearing the device at all times for the subsequent 7 days.[19] Participants will be asked to wear the accelerometer at baseline and again at follow-up to detect differences in physical activity and sedentary behaviors.

7.5.8 Falls and mobility

This is an interviewer-administered questionnaire given to each participant to record living circumstances, self-reported physical ability, fatigue, and falls.

7.5.9 Hospitalizations

This is an interviewer-administered questionnaire given to each participant to record recent hospitalizations since the last time of study contact.

7.5.10 Grip strength

Grip strength will be measured using standardized study protocols from the parent study (ARIC). These are detailed in the MOP. Grip strength (kg of force) is objectively assessed with a hand-held dynamometer. After one practice trial, participants are asked to complete two trials, squeezing as hard as possible, with a 15-20 second rest between trials. Grip strength is not completed for those who have had surgery on both hands or on both wrists in the previous 3 months. If only one side is affected, the unaffected side is tested. The test can be performed if the participant has a current flare-up of pain in their wrist or hand, for example arthritis or tendonitis. This information is recorded on the data collection form.

7.5.11 Short Physical Performance Battery (SPPB)

The Short Physical Performance Battery[20] will be conducted using standardized study protocols from the parent study (ARIC). These are detailed in the MOP. The SPPB is a series of physical performance tests designed to assess lower extremity function in older adults. The SPPB ranges in score from 0-12; higher scores indicate better function. The total score is the sum of 3 component scores: chair stands, balance, and 4-meter walk; each component score ranges from 0-4. Exclusion from any performance test is based on examiner assessment or participant concerns that the test would be unsafe. Walking aids are allowed during the 4-meter walk only, if participants feel they are necessary.

7.5.12 Intervention Feedback Form

This gathers feedback about the study intervention that participants are receiving. Participants rate how strongly they agree or disagree with a series of statements about the ACHIEVE study intervention they received.

7.6 Communication partners assessments

Communication partners (e.g., spouse) of participants randomized to the hearing intervention group are invited to join the study and contribute data related to their own quality of life and their observations of the effects of the hearing intervention on the participant. Communication partners are adults (18+ years) who communicate with the participant on a daily or near-daily basis. Informed consent is obtained during the 1-2 months after randomization when the hearing intervention participant is receiving the first 2 study intervention visits which the communication partner may also be attending in a supportive role. Demographic data will be gathered at enrollment, and health-related quality of life data (RAND36; 7.5.4) and data on the communication partner’s observations of the effects of the hearing intervention on the participant (International Outcomes Inventory – Significant Other) will be gathered semi-annually to annually. Individuals who are also enrolled in the ACHIEVE study as a randomized participant are not eligible to also be enrolled/consented as a communication partner in the ACHIEVE study for another randomized participant (e.g., spousal pairs randomized as a dyad to the hearing intervention group). If the communication partner can no longer participate, data collection for the communication partner will end. Participants are welcome to invite a new communication partner to join them at the study visits but no data will be gathered from this communication partner.

7.7 Schedule of evaluations

Table 3 details when data will be gathered. Data collection visits may be split across multiple days or multiple settings (clinic visits, home visits, telephone contacts, or video calls) to meet the needs of our participants.

Table 3. Schedule of Evaluations

Assessment	Pre-screening	Screening	Baseline	Intervention and Follow-Up Visits									
		Visit 0	Visit 1	Visit 2	Visit 3	Visit 4	Visit 5	Visit 6	Visit 7	Visit 8	Visit 9	Visit 10	Visit 11
		(Day -31 to Day 0)	Day 0	(W1-3)	(W3-5)	(W6-8)	(W8-10)	(6mo)	(12mo)	(18mo)	(24mo)	(30mo)	(36mo)
				A	B	C	D		Yr1		Yr2		Yr3
**Inclusion Criteria and Covariates**													
Informed Consent		X	X										
Demographics	X[1]		X										
Health History Form			X						X		X		X
Hearing and Noise exposure			X										
Vision Screening		X											
Activities of daily living	X												
Anthropometry			X[2]						X		X		X
Seated blood pressure			X[2]						X		X		X
Blood draw for ApoE			X[1,3]										
WRAT			X[1]										
Neurologic history			X						X		X		X
**Audiometry**													
Air conduction audiometry	X[1]	X							X		X		X
Bone conduction audiometry		X							X[4]		X[4]		X[4]
Tympanometry		X							X		X		X
Word Recognition in Quiet		X							X		X		X
Quick Speech in Noise (unaided)		X							X		X		X
**Neurocognitive Battery**													
Ensuring speech understanding test			X[2]					X	X	X	X	X	X
MMSE		X[2]	X[2]					X	X	X	X	X	X
Delayed Word Recall Test			X[2]						X		X		X
Digit Symbol Substitution Test			X[2]						X		X		X
Incidental Learning			X[2]						X		X		X
Trail Making Test Part A			X[2]						X		X		X
Trail Making Test Part B			X[2]						X		X		X
Logical Memory 1			X[2]						X		X		X
Logical Memory 2			X[2]						X		X		X
Digit Span Backward			X[2]						X		X		X
Boston Naming Test			X[2]						X		X		X
Word Fluency (FAS)			X[2]						X		X		X
Animal Naming			X[2]						X		X		X
**Non-Neurocognitive Measures**													
Clinical Dementia Rating Scale - Participant (CDP)			X						X		X		X
Dementia/MCI evaluation if applicable[5] (CDI, CDS, NPI)			X[5]						X[5]		X[5]		X[5]

Assessment	Pre-screening	Screening Visit 0 (Day -31 to Day 0)	Baseline Visit 1 Day 0	Visit 2 (W1-3) A	Visit 3 (W3-5) B	Visit 4 (W6-8) C	Visit 5 (W8-10) D	Visit 6 (6mo)	Visit 7 (12mo) Yr1	Visit 8 (18mo)	Visit 9 (24mo) Yr2	Visit 10 (30mo)	Visit 11 (36mo) Yr3
				Intervention and Follow-Up Visits									
Adverse event assessment/ Unanticipated problems		X	X	X	X	X	X	X	X	X	X	X	X
CES-D Scale			X[2]					X	X		X		X
Hearing Handicap Inventory for the Elderly - Screening Version			X[2]					X	X		X		X
RAND 36-Item Health Survey			X					X	X		X		X
Cohen Social Network Index			X					X	X		X		X
UCLA Loneliness Scale			X					X	X		X		X
Accelerometry			X						X		X		X
Falls and mobility questionnaire			X						X		X		X
Hospitalizations			X					X	X	X	X	X	X
Grip Strength			X[2]						X		X		X
Short Physical Performance Battery			X[2]						X		X		X
Physical Activity Form			X						X		X		X
Intervention feedback form								X					X
COVID impact questionnaire[6]										X	X	X	X
**SUCCESSFUL AGING INTERVENTION PARTICIPANTS ONLY**													
Successful aging intervention visit/booster session				X	X	X	X	X	X	X	X	X	
**HEARING INTERVENTION PARTICIPANTS ONLY**													
**Hearing Intervention Assessment Outcomes**													
Real-ear aided response					X	X[7]	X[7]	X[7]		X[7]		X[7]	
Speech Intelligibility Index (aided)					X	X[7]	X[7]	X[7]		X[7]		X[7]	
Quick Speech in Noise (aided)							X		X		X		X
Hearing Aid Data Log					X	X	X	X	X	X	X	X	X
International Outcome Inventory for comprehensive intervention							X	X	X	X	X	X	X
Client-Oriented Scale of Improvement				X		X	X						
**COMMUNICATION PARTNERS FOR PARTICIPANTS RANDOMIZED TO HEARING INTERVENTION**													
Informed Consent Form				X[8,9]	X[8,9]								
Demographics				X[8,9]	X[8,9]								
RAND 36-Item Health Survey				X[8,9]	X[8,9]			X[9]	X[9]		X[9]		X[9]
International Outcome Inventory – Significant Other							X[9]		X[9]		X[9]		X[9]

1. Procedures only needed for participants not in ARIC-NCS.
2. Measures shared across ACHIEVE and ARIC-NCS V7.
3. Typically obtained at the baseline visit, but could be obtained at a follow-up visit if not obtained at the baseline visit.

Assessment	Pre-screening	Screening	Baseline	Intervention and Follow-Up Visits									
		Visit 0	Visit 1	Visit 2	Visit 3	Visit 4	Visit 5	Visit 6	Visit 7	Visit 8	Visit 9	Visit 10	Visit 11
		(Day -31 to Day 0)	Day 0	(W1-3)	(W3-5)	(W6-8)	(W8-10)	(6mo)	(12mo)	(18mo)	(24mo)	(30mo)	(36mo)
				A	B	C	D		Yr1		Yr2		Yr3

4. Only required at initial hearing test or follow-up visits if there has been a change in pure-tone air conduction.
5. Collected if participant is selected for informant interview, identified on the Selected to Stage 2 report in the study database.
6. Added in January 2021.
7. Only required when the participant needs a programming adjustment in the software.
8. Only needs to be completed once at either visit A or B.
9. Only completed if communication partner is present for the visit and willing to participate.

8 Randomization

Randomization, stratified by ARIC study status, field center, and hearing impairment level is completed within the Carolina Data Acquisition and Reporting Tool (CDART) web-based data management system. Although ACHIEVE hearing aid intervention is by nature un-masked, in order to minimize bias based on review of accumulating data by the project team, the ACHIEVE PI, co-investigators, and key project staff except Data Coordinating Center staff and one unblinded statistician will remain blinded to accumulating data by treatment group.

- The intervention group to which a participant is assigned is determined by an allocation schedule developed by the Data Coordinating Center.
- After final determination of eligibility and confirmation of informed consent, each participant will be randomized in a 1:1 ratio to either the hearing intervention or to the successful aging intervention. To ensure balance between the treatment groups, participants will be randomized in permuted order blocks of varying sizes within strata defined by severity of hearing loss, defined as mild (PTA ≥ 30 dB HL and < 40 dB HL) or moderate (PTA ≥40 dB HL and < 70 dB HL), participant status (ARIC or non-ARIC participant), and by field site. Block size will not be revealed to field center staff as this would allow them to determine the final treatment assignment of a block before ascertaining eligibility and obtaining consent.
- Randomization will be performed at the end of the baseline visit.
- When spouses or cohabitating partners are both eligible and randomized together, they will be randomized as a unit with, arbitrarily, the first spouse/partner of the pair to be selected according to the random assignment procedure and the second spouse/partner of the pair receiving the same assignment. Spouse/partner pairs will be randomized in spouse/partner-pair specific permuted order blocks of varying sizes within strata defined by participant status (at least one spouse/partner pair in ARIC or both non-ARIC participants) and by field site.

9 Interventions

9.1 Hearing intervention

The conceptual framework for the development of the manualized patient-centered, best-practices hearing intervention was the World Health Organization's International Classification of Functioning, Disability and Health (ICF, http://www.who.int/classifications/icf/en/). The ICF focuses on a person's ability to engage in activities and participate in life situations, as impacted by changes in body structures and/or functions, and influenced by environmental and personal contextual factors. The main objective of intervention is improving a person's quality of life by eliminating or minimizing activity limitations and participation restrictions. To reach this objective the hearing intervention includes the identification of individual needs, the setting of specific goals, engagement in shared-informed decision-making, and the development of self-management abilities.

The hearing intervention (HI) steps were developed from review of the most current and comprehensive evidence-based practice guideline for the rehabilitative treatment of hearing loss in adults published by the American Academy of Audiology (AAA) in 2006, along with consideration of subsequent research. An outline of the ACHIEVE-HI components was reviewed by an expert panel of audiologists (including M. Valente, L. Humes, H. Solodar, and R. McArdle among others) during a meeting in Tampa, FL in December 2014. Recommended modifications were incorporated into the hearing intervention, which was utilized in a feasibility study conducted at the University of South Florida with 20 older adult participants and initiated in May 2015. The results of implementation in the feasibility study guided the use of the hearing intervention in the subsequent ACHIEVE-P randomized pilot study which was initiated at the Washington County, MD ARIC field site in August 2015. Experiences of study participants and site staff from both the feasibility and pilot studies were used to revise the hearing intervention, and this revised protocol was then reviewed in a face-to-face meeting of ACHIEVE investigators and an external expert audiology consultant, C. Palmer in April 2016. Recommendations for clarification were incorporated into the final hearing intervention protocol which is included in the MOP.

The hearing intervention consists of 4 sessions (Sessions A-D, each session ~1 hr) spaced over the 2-3 months post-randomization and are designed to provide all of the active components of the intervention. Audiologic outcomes (e.g., hearing aid data logging, real ear measures, speech in noise, etc.) to verify the best-practices hearing intervention are gathered semi-annually beginning at 6 months post-randomization. Communication partners (CP) are often a key to success to intervention for older adults with hearing loss and are encouraged to attend the hearing intervention sessions.

Session A is focused on the identification of individualized goals utilizing the Client Oriented Scale of Improvement (COSI) and the fitting of hearing aids (Session A). In addition to standard audiometric testing conducted in all ACHIEVE participants at baseline, an auditory needs assessment, involving specialized behavioral and self-report assessments, is completed in order to determine patient-specific recommendations for hearing aid intervention, including determination of signal processing and features (e.g., directional microphones, direct auditory inputs, noise management programs, frequency modulation [FM] devices, Bluetooth streaming, etc.). Participants will be fit binaurally with substantively equivalent technology that is currently found in Phonak Audéo V90-312T receiver-in-the-canal (RIC) hearing aids. Specification for hearing aid fitting and verification can be found in the MOP. Participants will be provided with materials to support self-management of hearing loss and communication in real-world settings, via the hearing intervention Toolkit (full details in MOP). The toolkit materials were developed based on previous research by Hickson and colleagues and with consideration of variations in health literacy, and cover the following topics: understanding hearing loss, realistic expectations, communicating in quiet, listening at a distance, communicating in background noise, using communication strategies and tactics, and resources for adults with hearing loss and their communication partners. The written materials are supplemented with a series of reusable learning objects (e.g., video clips) entitled C_2HEAR which are designed to support first-time hearing aid users while they adapt to using hearing aids.

Session B occurs approximately 1-3 weeks after hearing aid fitting in order to allow for acclimatization to occur. If modifications are needed to the hearing aid fitting, they can occur and be documented in this or any subsequent sessions. During Session B, informal assessment of progress toward goals will be determined and needs for various hearing assistive technologies (HATs) will be assessed. HATs provide a more direct coupling of the sound source to the hearing aid, minimizing the negative effects of distance and background noise. Additional materials to support self-management are provided.

Session C occurs approximately 1-3 weeks after Session B. Short-term hearing aid outcomes are assessed and individually selected HATs are dispensed. Each participant will receive at least one HAT, depending on his or her specific goals, and outcomes of hearing aid use. These HATs may include the following or generally equivalent devices: Phonak ComPilot II (streaming device); Phonak ComPilot Air II (streaming device); Phonak TVLink II; Phonak clip-on mic; and/or the Phonak Roger System. Systematic orientation to HAT use is provided and additional self-management tools are dispensed.

Session D occurs approximately 1-3 weeks after Session C. Short-term outcomes of the comprehensive intervention approach, including use of hearing aids, HATs, and development of self-management skills, are assessed. Additional materials as needed to support self-management are provided as well as a supply of batteries.

Booster Intervention/Interim Visits: Scheduled semi-annual visits will capture audiologic outcomes and verify that hearing aids and HATs are functioning properly. Re-instruction in use of devices and hearing rehabilitative strategies will be provided during these visits. Booster sessions may be offered in a group format to allow for group rehabilitation for participants who are interested. We anticipate that unscheduled interim visits may also be required periodically (e.g., hearing aid malfunction), and these visits to trouble-shoot and/or repair malfunctioning hearing aids and/or HATs will be scheduled as needed if the issue cannot be resolved through a telephone conversation.

Full details of the hearing intervention can be found in the MOP.

9.2 Successful Aging intervention

Overview

The successful aging health education control intervention (Successful Aging intervention, or SA intervention) will follow the protocol and materials developed for the 10 Keys™ to Healthy Aging program by the Center for Aging and Population Health Prevention Research Center at the University of Pittsburgh.[1] This interactive, dynamic program informs older adults about risk factors for diseases. This program was developed from evidence-based research and aims to target the causes of disease and disability that could be greatly reduced, postponed, or eliminated to promote successful and healthy aging in functional older adults. The Successful Aging intervention contains the most up-to-date prevention guidelines available based on the current recommendations from leading groups such as the United States Preventive Services

Task Force, Centers for Disease Control, and Institute of Medicine. Many adults age with more than one medical condition. A holistic approach to health provides a broad range of opportunities for individuals to become engaged and involved. This is important as the Successful Aging intervention focuses on the health and well-being of the whole person. Although the original "Keys" had 10 domains, the "Social Contact" Key will not be used (to avoid crossover effects with the main hearing loss intervention being tested), and a "Caregiving and Health" Key will instead be used for individuals for whom the smoking Key is not applicable.

Individual participants in the Successful Aging intervention group will receive educational information and materials on health-related topics relevant to older adults. As a participant in the Successful Aging intervention, each individual will have an opportunity to practice skills and learn about: the importance of preventing disease and disability, information on Keys or health topics of his/her choice, and personal risk factors. In general, participants will receive information on a variety of health topic areas of relevance to older adults and learn how to actively "take charge" of their health and well-being in seeking out appropriate medical information, services, and resources. The health educator will tailor the Successful Aging intervention to each participant and provide guidance through the educational materials and ongoing support and encouragement over the duration of the study.

At the first intervention visit, using a Prevention in Practice (PIP) report, the interventionist will present an introduction to all of the Keys and support the participant in identifying the specific topics of importance. At the first visit the interventionist will also go through the lowering systolic blood pressure key (this key was identified as the most popular key by Dr. Glynn and colleagues). Additionally, the interventionist will teach the participant proper technique for a series of upper body stretches. The second, third and fourth intervention sessions will contain a recap of the previous session and a new key. The participant will also do their body stretching routine. Each session will last approximately one hour, and each Key is designed to be presented during this time. Regardless of the specific Key being presented, each session will include a didactic education component as well as activities, goal-setting, and homework assignments to be completed by the following session.

Specific Successful Aging Module Options

Each participant randomized to the Successful Aging intervention will have the opportunity to cover the following Keys or health topics:

Key #1: Lower Systolic Blood Pressure
Key #2: Stop Smoking (if the participant is a smoker; if the participant is not a smoker they will skip this key)
Key #3: Participate in Cancer Screening
Key #4: Get Immunized Regularly
Key #5: Regulate Blood Glucose
Key #6: Lower LDL Cholesterol
Key #7: Be Physically Active
Key #8: Maintain Healthy Bones, Joints, and Muscles
Key #9: Combat Depression
Key #10: Caregiving and Health

Contact Mode and Frequency

Participants randomized to this group will meet individually with a health educator certified to administer the program every 2-3 weeks for a total of 4 visits over about 2-3 months. This program was effectively piloted in this cohort and previously implemented by Dr. Glynn in the Aging Successfully with Pain RCT.[21,22] To further enhance retention and perceived benefit, each session will also include a 5-10 minute active upper body extremity stretching program as used in the Lifestyle Interventions and Independence for Elders (LIFE) study.[2] Participants will return for booster sessions semi-annually (+/- about one month). Session content for booster sessions will be keys 5-9 (or 6-10 for non-smokers). The upper body stretching program will also be implemented during the booster sessions.

Visit 1 (Session A): Weeks 1-3
Visit 2 (Session B): Weeks 3-5
Visit 3 (Session C): Weeks 6-8
Visit 4 (Session D): Weeks 8-10

Participants who miss a scheduled visit will be contacted by intervention staff to reschedule or to assist with overcoming barriers to attendance (e.g., transportation). Additional support will be provided by aging experts from the University of Pittsburgh who have years of experience overcoming barriers associated with adherence to the program.

Full details of the Successful Aging intervention can be found in the MOP.

10 Strategies to promote intervention adherence/compliance & minimize bias from unblinded study participants and staff

Adherence to the study intervention will be assessed at each study visit using questions designed to capture adherence in both the hearing and successful aging intervention groups.

Strategies for Promoting Adherence in the Hearing Intervention

Strategies to promote adherence in the Hearing Intervention include:

- Expectations of potential benefits of the intervention are structured given the participant's level of hearing impairment in order to ensure that participants' expectations are reasonable and realistic.
- Individual assessment and instruction.
- Participants are asked to bring a communication partner with them to the intervention visits.
- Repeated assessment of individualized hearing goals with the audiologist and development of strategies to meet those goals throughout the intervention period.
- Participants who miss a scheduled meeting are contacted by telephone by study staff to encourage continued participation and to evaluate and overcome barriers to participation.

- Home visits for intervention sessions will be considered on a case-by-case basis by the study staff and interventionist when needed because of participant inability to travel to clinic-based study visits.
- Participants are informed that they are allowed to keep the hearing aids and other hearing technologies for free if they complete all study visits.
- At the completion of the study, participants in the hearing intervention may also choose to receive the successful aging intervention.

Strategies for Promoting Adherence in the Successful Aging Intervention

Strategies to promote adherence in the Successful Aging Intervention include:

- Expectations of potential benefits of the intervention are structured in order to ensure that participants' expectations are reasonable and realistic.
- Individual assessment and instruction.
- Participants can bring a friend or family member with them to intervention visits (analogous to the communication partner for participants randomized to the hearing intervention).
- Establishes concrete and reasonable goals related to attending the Successful Aging sessions and participating in that intervention throughout the intervention period.
- Easy-to-read written materials are distributed at each visit to prompt regular and appropriate participation.
- Participants are encouraged to track behavior changes related to topics covered as part of the program.
- Participants who miss a scheduled meeting are contacted by telephone by study staff to encourage continued participation and to evaluate and overcome barriers to participation.
- Home visits for intervention sessions will be considered on a case-by-case basis by the study staff and interventionist when needed because of participant inability to travel to clinic-based study visits.
- At the completion of the study, participants in the successful aging intervention may also choose to be fitted for hearing aids and provided hearing rehabilitation resource materials if they are interested.

Strategies to Minimize Bias from Unblinded Study Participants and Staff

Neither study participants nor study technicians collecting outcome data can feasibly be blinded to randomization status. We will minimize bias from unblinded study participants and staff through the following strategies based on recommendations from Boutron et al.[23] for blinding in non-pharmacological trials:

- <u>Use of an attention control intervention</u> Participants are randomized to one of two interventions – hearing rehabilitative intervention vs. an established successful aging intervention that serves as an attention control. This successful aging intervention is

based on the 10 Keys™ to Healthy Aging program developed by the Center for Aging and Population Health Prevention Research Center at the University of Pittsburgh[1] and which was successfully implemented as the attention control intervention in the unblinded Lifestyle Interventions and Independence for Elders (LIFE) study.[2] The use of an attention control intervention that is perceived as having value to study participants can minimize participant dissatisfaction with not obtaining the hearing loss intervention. In the ACHIEVE-Pilot study, study staff reported strong participant enthusiasm for the successful aging intervention. The study overall will communicate that it has two interventions aimed at successful aging. They are very different and expected to have different benefits which are unknown. The goal is to identify which intervention has which benefits including cognition and other measures of aging.

- Blinding of study participants to study hypothesis Informational material and the consent provided to study participants will emphasize that the ACHIEVE study is comparing two established interventions in which it is not known whether either intervention could affect cognitive decline. Blinding of study participants to the explicit hypothesis that the hearing intervention reduces cognitive decline can help promote acceptance and adherence to both study interventions.
- Standardized protocols for training of data collectors & assessment of study outcomes Strict and standardized protocols for assessment of neurocognitive functioning will be implemented to minimize bias during data collection. Protocols (see Manual of Procedures) for both training/certification of psychometrists and quality assurance/control have been well-established and tested in both ARIC-NCS and the ACHIEVE-Pilot study. These protocols incorporate regular recordings/observations of data collection sessions for feedback/quality control and centralized training of all psychometrists on a standardized protocol for neurocognitive test administration. Importantly, we will also utilize a supplementary standardized protocol incorporating an assessment of spoken language understanding to ensure that speech understanding is not confounding neurocognitive testing or other data assessments (section 7.2.2). Dementia adjudication procedures based on consensus conference review of study data will be blinded to randomization status. These procedures will help minimize potential bias from unblinded data collection staff.
- Data collectors & study coordinators will not have access to cognitive testing results from prior study visits Cognitive test data from prior study visits will not be available to data collectors and study coordinators in order to avoid unintentional and possibly unconscious bias by study staff during data collection. All data collection staff are distinct from staff who are involved with intervention administration. In addition, the primary and key secondary outcomes based on cognitive testing factor scores described in section 12.3 will not be made available to the site study staff.

Of note, the ACHIEVE Steering Committee did consider at length alternate study designs to allow for blinding of study technicians including having all participants use a body-worn sound amplifier at all visits, having all participants use no hearing devices at any visits (to mimic the state at baseline testing), having all participants wear “covers” over their ears, or having participants randomized to successful aging wear “sham” hearing aids during testing. The

former two approaches would adversely affect neurocognitive testing (in which prevailing neuropsychometric standards stipulate that participants use whichever sensory aids they use on a regular daily basis). All four approaches were found to be pragmatically infeasible through discussion with field site staff and potential study participants.

11 Safety monitoring

11.1 Data and safety monitoring board

An independent Data and Safety Monitoring Board (DSMB) has been constituted in collaboration with the NIA. The DSMB will provide oversight to ensure that the trial accrues at a sufficient rate and that the safety and privacy of all study participants are maintained. DSMB members are clinicians/investigators with expertise in clinical trials, neurology, dementia, biostatistics, audiology, and otolaryngology. A DSMB charter which was approved by the DSMB and the NIA specifies guidelines for DSMB conduct and early stopping rules for safety and futility. We anticipate meetings of the DSMB every 6 months after the first year, always focused on participant safety, but initially focusing on recruitment and follow-up.

There will be no interim analysis for efficacy. We do not expect to see an efficacy signal of sufficient magnitude to stop the trial with periods of follow-up shorter than 3 years. Combined with the comparatively shorter recruitment period, there is minimal expected benefit from an interim efficacy analysis. A formal interim analysis to evaluate for futility and sample-size re-estimation will be performed after 66% of subjects have completed the study, as described below in section 12.4.

11.2 Adverse events

Study participation and exposure to the hearing aid intervention is expected to have a low risk of adverse events for the participant. At the same time, the age of the participants may naturally lead to numerous deleterious health outcomes. In order to efficiently collect safety information that is relevant to study participation, interventions, and procedures, detailed information concerning a **pre-specified set** of adverse events and serious adverse events will be collected and evaluated throughout the conduct of the trial.

An ***adverse event (AE***) is an untoward medical occurrence, whether or not considered study-related, which occurs during the conduct of a clinical trial

A ***serious AE*** **(SAE)** is any AE that results in any of the following outcomes:

- Death
- Life-threatening
- Inpatient hospitalization or prolongation of existing hospitalization
- Results in persistent or significant disability or incapacity
- A *major* congenital anomaly or birth defect
- Important medical event that may not result in one of the above outcomes, but may jeopardize the health of the study participant or require medical or surgical intervention to prevent one of the outcomes listed above.

For this study, ***only the following adverse events and serious adverse events will be recorded and reported***:

Adverse Events

- Otitis externa
- Cerumen impaction or ear foreign body requiring removal by a physician

Serious Adverse Events

- Death from any cause

An adverse event of otitis externa is defined as inflammation or infection of the ear canal resulting in pain, swelling, irritation, itching, or other related symptoms as diagnosed by the study audiologist or a physician. Adverse event severity is defined as:

- **Mild** if the symptoms are self-limited and resolve with interventions such as transiently limiting hearing aid use and/or the use of over-the-counter pharmacological therapies such as hydrocortisone cream or swimmer's ear drops.
- **Moderate** if the symptoms require evaluation and management by a physician *and* the use of topical prescription pharmacological therapies such as antibiotic ear drops.
- **Severe** if the symptoms require evaluation and management by a physician and the use of oral or parenteral antibiotics.

An adverse event of a cerumen impaction or ear foreign body requiring removal by a physician is defined as a cerumen impaction and/or ear foreign body that cannot be routinely managed by the study audiologist and requires evaluation and management by a physician (typically an otolaryngologist). Adverse event severity is defined as:

- **Mild** if the cerumen impaction or foreign body is resolved without further need for therapy besides over-the-counter pharmacological therapies such as cerumenlytic drops.
- **Moderate** if there is an associated otitis externa requiring the use of topical prescription pharmacological therapies such as antibiotic ear drops.
- **Severe** if the cerumen impaction or ear foreign body results in a perforation of the tympanic membrane or an associated otitis externa requiring the use of oral or parenteral antibiotics.

AEs or SAEs will be defined as **unexpected** or **expected** based on the judgement of the field site PI in consultation with the study audiologist and/or study PI Lin (who is a board-certified otolaryngologist) based on the following definitions:

- **Unexpected** – nature, severity, or frequency of the event is not consistent with information about the condition under study or intervention in the protocol and consent form

- **Expected** – event is known to be associated with the intervention or condition under study.

Study-relatedness of AEs or SAEs will be based on the judgement of the field site PI in consultation with the study audiologist and/or study PI Lin based on the following guidelines:

- **Definitely Related:** The adverse event is clearly related to the investigational intervention – i.e., an event that follows a reasonable temporal sequence from administration of the study intervention, follows a known or expected response pattern to the suspected intervention, that is confirmed by improvement on stopping and reappearance of the event on repeated exposure and that could not be reasonably explained by the known characteristics of the subject's clinical state.
- **Possibly Related***:* An adverse event that follows a reasonable temporal sequence from administration of the study intervention, that follows a known or expected response pattern to the suspected intervention, but that could readily have been produced by a number of other factors.
- **Not Related***:* The adverse event is clearly not related to the investigational agent/procedure - i.e., another cause of the event is most plausible; and/or a clinically plausible temporal sequence is inconsistent with the onset of the event and the study intervention and/or a causal relationship is considered biologically implausible.

Unanticipated Problems

An unanticipated problem is defined as an incident, experience, or outcome that meets all of the following criteria:

- Unexpected – in terms of nature, severity, or frequency given the procedures and interventions used in the ACHIEVE study as described in the protocol and informed consent given the characteristics of the study population
- Possibly or definitely related to the participation in the ACHIEVE study
- Suggests that the research placed the participant or others at a greater risk of harm (including physical, psychological, economic, or social harm) than was previously known or recognized

Some but not all adverse events may qualify as unanticipated problems.

The DSMB will review all recorded treatment-emergent adverse events and all serious adverse events (SAEs) from this pre-specified list and will provide a report to the Steering Committee and to the local IRBs. An SAE that is ***unexpected and possibly or definitely related to study participation or study intervention*** will be reported within 48 hours by the sites to the local IRB and the PI to the NIA and DSMB.

Adverse events and serious adverse events will be recorded on the electronic Adverse Events Form per study instructions. Additionally, for ***unexpected and possibly or definitely related***

serious adverse events only, sites will record a narrative description of the serious adverse event, including any relevant lab test results and dates.

All completed AE forms will be forwarded promptly to the Coordinating Center where they will be reviewed for completeness by the Coordinating Center staff and the ACHIEVE Principal Investigator (Lin). In particular, the Principal Investigator will assure that documentation of each event is adequate to permit accurate inferences regarding causation (e.g., temporal associations, onset, course, response to patient or physician intervention, alternative etiologies) and severity.

Full details of procedures for reporting adverse events can be found in the MOP.

12 Statistical considerations

12.1 Sample size

Aim 1: Comparison of hearing rehabilitative intervention versus successful aging control intervention for 3-year cognitive decline

Our sample size calculation is based on the primary outcome, the change in the Global Cognitive Function Factor Score from baseline to year 3. Estimates are based on the rate of change in the global cognitive function factor score from the first third of participants in the ongoing ARIC Visit 6 study (2016 to 2017) in addition to data from other representative studies with similar cohort characteristics as ARIC/ACHIEVE (i.e., ACTIVE and HealthABC). We also take into account assumptions regarding the rate of loss to follow-up as well as the rate of cross-over, where participants in the successful aging control group begin to wear hearing aids and participants in the hearing intervention group stop wearing the devices.

We based these calculations on a t-test comparing groups on change from baseline at 3 years to align with the primary analysis (a mixed effects model treating time as categorical) and aimed to achieve 90% power using a 2-sided test with a p=0.05 significance level. We conservatively chose parameter estimates that fell within the mid-range of observed values from these studies.

Parameter assumptions include:

- Change in global cognitive function factor score in the control group per year: Estimated at -0.08 standard deviations (SD)/year (corresponding to -0.24 SD in 3 years)

- Standard deviation of change in global cognitive function factor score per year: Estimated at 0.09 (corresponding to 0.27 at 3 years)

- Drop-in/Drop-out: Estimated as a net total of 15% (drop-in + drop-out) for the 3-year study. This value is conservative. We assumed immediate drop-in/drop-out rather than later drop-in/drop-out (i.e., 1-2 years into the study) which would have less impact on reducing the observed effect size. We will continue to optimize the design to keep both

drop-in and drop-out rates low (e.g., rigorous training for active group vs. energized successful aging group promised free hearing aids at trial completion). Among 13 participants in the ACHIEVE-Pilot study who were randomized to the hearing intervention and have since been followed to 1 year, we did not observe any drop-out (discontinuation of hearing aid use) at 1 year (12 participants reported > 8 hours of hearing aid use/day and 1 participant reported 4-8 hours use/day). Similarly, drop-in rates will likely be minimized because all participants in the successful aging group are informed that they will receive free hearing intervention after 3 years of follow-up (out-of-pocket costs for hearing aids are otherwise currently ~$4700 and are rarely covered by insurance). Rates of uptake of hearing aid use remain low in the community. In the HealthABC study among individuals with hearing loss in the inclusion range for the ACHIEVE study, rates of hearing aid uptake were ~3-4% per year.

- Missing data/withdrawal from competing events (e.g., mortality not associated with dementia): Estimated at 10%/year (corresponding to 27% in 3 years)

Based on these parameters and with 850 total individuals randomized, we will have 90% power to detect a 35% difference in annual cognitive decline between the hearing intervention vs successful aging intervention at 3 years follow-up.

Aim 2: For the secondary outcome of incident cognitive impairment, a dataset was constructed of participants in the ARIC cohort who had comparable characteristics to the individuals enrolled in ACHIEVE, did not exhibit signs of cognitive impairment at the baseline, and who were monitored over a period of 5 to 8 years. The observed rate was 50 cases of incident cognitive impairment per 1000 person-years. Assuming a 30% reduction in the hearing intervention condition and a 25% loss of person-time due to attrition, the planned analysis would have 80% power to detect a hazard ratio of 0.54 when using a 2-sided test and $p < 0.05$.

12.2 Analytic approach

Analyses will follow the intention-to-treat (ITT) principle in which subjects will be analyzed in the group to which they were randomized, regardless of whether or not they received the assigned intervention. Primary analyses will be based on the ITT population, which includes all randomized subjects. In addition, a secondary analysis of the primary outcome will be completed for the per-protocol (PP) population, defined as a subset of the ITT population who completed the 8-10 week intervention period, had no hearing aid intervention drop-in for the control group, and had no major protocol deviations. Major protocol deviations include violations in inclusion and exclusion criteria at enrollment and poor compliance with hearing aids for the hearing aid intervention group, defined as subjects who discontinue hearing aid use. All major protocol deviations will be identified in a blinded fashion prior to database lock. Complier average causal effect analysis will also be conducted to mitigate bias present in per protocol analyses.

Consistent with best practices in clinical trials, we will assess the comparability of the randomized groups with respect to known baseline confounders such as hearing loss (pure-tone average in the better hearing ear <40 dB vs ≥40 dB), recruitment source (ARIC vs de novo),

field center, age, sex, education, and the presence of APOE ε4 alleles.[24] If, despite randomization, there is an imbalance between the treatment conditions, we will adjust for relevant confounders as well as explore the use of a precise, locally efficient, augmented, simple estimator[25] which may offer greater precision.

Primary Outcome

The primary outcome is cognitive decline, as measured by the change in a global cognitive function factor score. The factor score is derived from a confirmatory factor analysis model that identifies common covariation among all cognitive tests administered in-person. Factor scores are generated for each participant at each in-person assessment using a measure harmonization and item banking approach.[26, 27] Factor loadings and latent means for each neurocognitive test are estimated using data from the ACHIEVE baseline.

Primary Analysis

Groups will be compared for the primary outcome using mixed effects models that account for the correlation among repeated measures as well as the correlation between spouses or cohabitating partners. If a linear trend appears reasonable, we will fit a model with a linear slope. If a nonlinear trend is observed, the model will be adapted to include time splines.[28] Continuous time in years from the baseline will be the time scale. An interaction term between treatment assignment and time will be used to test if rates of cognitive change differ by treatment assignment. Model fit will be assessed with residual plots and other statistics (Akaike Information Criterion, Bayesian Information Criterion, etc.). The primary analysis may include adjustments for the baseline hearing loss, ARIC vs de novo status, field center, age, sex, education, and APOE ε4 alleles.

Missing cognitive factor scores among ACHIEVE participants will be generated utilizing multiple imputation by chained equations.[29] The number of imputations needed to generate valid parameter estimates will be determined by a two-stage analysis.[30] The imputation model will include (1) in-person cognitive factor scores, (2) MMSE and Six-item Screener scores, (3) adjudicated incident MCI or dementia, (4) race, (5) time variables indicating when a participant with missing data might have completed an assessment based on time from randomization to missed visit, and (6) all previously listed covariates. Interactions between variables in the imputation model will be tested and added as necessary. The imputation will be conducted in stages so that concurrent and past measurements, but not future measurements, inform the imputed values. The validity of the imputation model will be assessed by comparing observed values to imputed values among a 20% sample selected at random and a 20% sample selected based on the probability of missingness estimated from a logistic regression model.

The primary analysis will focus on cognitive factor scores imputed prior to death. An analysis comparing pre- and post-death cognitive factor scores will be performed using values generated from an imputation model in which death is included as an auxiliary variable. A similar procedure will be used to contrast pre- and post-dementia cognitive factor scores.

Sensitivity Analyses of the Primary Outcome

Additional analyses of the primary outcome may include, but are not limited to:

(a) An analysis of global cognitive function in which data from in-person cognitive evaluations will be stratified by condition before a factor score is computed for each participant. The factor scores generated will be standardized using the condition-specific baseline mean and SD.

(b) An analysis of a co-calibrated[31,32] global cognitive function factor score in which the ten tests administered in-person and the six tests administered over the phone will be included in the confirmatory factor analysis model. Modeling constraints will be applied to scale the co-calibrated factor score to the same metric as the in-person factor score utilized in the primary analysis.[33] Mode of data collection (in-person, phone) will be included in the analytic model as a covariate.

(c) Use of full-information maximum likelihood to account for missing data. Parameter estimates from these analyses will be compared to estimates from multiple imputation analyses to gauge the impact of including auxiliary variables in the imputation model.

(d) Control-based multiple imputation, in which missing values of the primary outcome for the hearing aid condition without a dementia diagnosis are imputed based on the successful aging control intervention condition. This analysis will investigate the strength of the primary analysis results to the missing at random assumption.

(e) Analysis of cognitive decline based on a categorized version of time (visits) rather than continuous time in years.

(f) A replication of the primary analyses stratified by subgroups of ARIC vs de novo participants. Interaction between intervention condition and recruitment group will be tested in additional models relative to $p < 0.10$.

(g) Exploration of the impact on the primary analysis by further adjustment for additional explanatory variables, as mentioned above.

Exploratory Analyses of the Primary Outcome

a) **Differential practice effects by recruitment source** The study recruited participants from ARIC and from a community sample. ARIC participants have had prior exposure to testing while non-ARIC participants have not. To address this potential limitation, we will use a mixed effects modeling framework to examine practice effects using an indicator for the first visit in each condition, allowing the practice effect parameter to vary by recruitment source and keeping the estimated intervention effect constant. We will test the fit of that model to one where the intervention effect differs by condition to evaluate whether differential practice effects by recruitment source affects the intervention condition difference. This is a test of effect modification.

b) **Does intervention alter the trajectory of cognitive decline** In the subset of ARIC participants, we will model prior cognitive change (before the baseline ACHIEVE visit) as well as prospective change through study end (2021-22) using linear spline models, to determine if the rate of change of cognitive decline during ACHIEVE in each condition is different than the rate of change in the preceding years in ARIC visit 5 and 6. We will also test the interaction hypothesis that the hearing intervention deceleration in decline is larger than that in the successful aging intervention condition. The power for analyses in the ARIC subgroup will be lower than optimal since the proportion of ACHIEVE participants recruited from ARIC was smaller than originally anticipated.

c) **Subgroup analyses by demographics** Given the lower prevalence of hearing loss in women compared to men and blacks compared to other races, an exploratory analysis will be conducted stratifying by sex, race, and education. We will also conduct analyses stratifying by level of hearing loss and level of baseline global cognitive function factor score. Interaction between intervention condition and subgroups will be tested in additional models relative to $p < 0.10$.

d) **Subgroup analyses by AD risk factors** We will investigate a further refinement of the primary analysis with subgroup analysis for known AD risk factors, including but not limited to ≥1 APOE ε4 allele, as well as diabetes or hypertension. A subset of ARIC participants will be defined as more likely to be at high risk of AD based on reduced temporal lobe volume meta ROI[33] by structural MRI and no small vessel disease (ARIC Visit 5, 2011-13).

e) **Exclusion of tests with only auditory stimuli** Among the full study cohort, we will derive a revised global cognitive function factor score excluding tests with only auditory stimuli (Logical Memory, Digits Backward). We will use model constraints in a latent variable modeling framework to scale these revised factor scores to be on the same metric as the factor scores in the primary analysis.[34]

f) **Mediation by social function** Investigate the extent to which the cognitive improvements are mediated by improvements *in social function* using causal mediation methods.[35]

Secondary Outcomes

An important secondary outcome is time until a composite of (1) adjudicated dementia determined from in-person or phone-based evaluations, (2) adjudicated MCI[26] determined from in-person evaluations (3) a 3-point drop in the 30-item MMSE administered in-person, or (4) a 3-point drop in a factor score derived from the 10-item MMSE orientation subscale and 11-item Blessed scale administered over the phone and rescaled to be equivalent to the 30-item MMSE. Intervention conditions will be compared for the time until the composite outcome utilizing a discrete-time, cause-specific proportional-hazards model with a complimentary log-log link. The same baseline covariates specified for the mixed effects model will be integrated into the proportional-hazards model. Time on study will be the time scale. As a sensitivity analysis, we will examine variations of the composite outcome that include (1) adjudicated MCI from phone-

based evaluations and (2) unadjudicated MCI diagnoses identified algorithmically. In exploratory analyses, we will analyze time until (1) adjudicated dementia or MCI diagnosis and (2) decline in MMSE and Blessed scores as separate events.

Three additional secondary outcomes that will be examined include decline in the cognitive domain factor scores for memory, executive function, and language[9] derived from in-person assessments. Intervention conditions will be compared using the same statistical methods described above for the primary outcome. Results from the analysis of the four secondary outcomes will be included in the primary outcome manuscript.

Adjustment for Multiple Comparisons

Statistical significance for the primary outcome will be defined as $p < 0.05$. Secondary outcomes will be evaluated for statistical significance with a Hochberg modification to the Bonferroni adjustment, in which the p-values of the five outcomes will be ordered. The largest p-value will be compared relative to $p < 0.05$, and if met, all parameters will be considered significant. If not, then the second largest p-value will be assessed relative to $p < 0.05/2 = 0.025$, and if met, then it and all other parameters will be considered significant, and so on for the third p-value compared to $0.05/3=0.017$, the fourth compared to $0.05/4 = 0.012$, and the fifth compared to $0.05/5 = 0.01$.

Additional Pre-Specified Outcomes

Additional measures include measures of social and physical function, physical activity, and HRQL. Analyses of all other outcomes are considered exploratory in nature and will not be viewed as providing confirmatory tests of hypotheses. There will be no adjustment for multiple comparisons of the exploratory pre-specified outcomes, and p-values will be provided for descriptive purposes only.

Outcomes will be modeled continuously (outcomes transformed to account for non-normality if necessary) or categorized according to clinically-relevant cut-points.[37] Intervention groups will be compared using the same approach adopted for the primary analysis.[37]

12.3 Global cognitive function factor score

Properties and advantages

The global cognitive function factor score uses all available cognitive test data, has interval-level properties,[38] is internally consistent using ARIC-NCS data (Cronbach's alpha=0.87), has minimal floor or ceiling effects,[39] and demonstrates reliable measurement precision over a broad range of cognitive ability. We have previously demonstrated criterion validity and established cut-points for clinically relevant impairment of the general cognitive performance factor score.[39] We have since extended the harmonization to 26 studies with over 60,000 people, most of which have longitudinal data. Using simulation, we verified the cognitive metric is the same across dataset.[39] We compared precision of our approach with other approaches to combining data using external data: underscoring enhanced precision in a sample of 10,875 persons in 9 datasets, our approach required the smallest sample size to detect cognitive decline with 80% power (N=232) compared to using only the MMSE (N=277) or summarizing available tests into a z-score (N=291).[34,40] We further validated the approach against change in hippocampal volume and overall cortical thickness.[34] The approach is consistent with other harmonization techniques and has been used in several other published studies[33, 39-44] including demonstrations that the factors have the same meaning across datasets with different cognitive tests.[33, 39-43, 45-49]

12.4 Assumptions analysis

An assumptions analysis took place in June 2019, prior to the closure of the initial recruitment period. The goal of the analysis was to evaluate a subset of the assumptions described above and assess the value of a possible increase in the sample size. Since ACHIEVE was not going to be stopped early for efficacy or futility, regardless of the interim results, and the interim analysis only investigated assumptions around attrition and drop-in/drop-out rates, not outcomes, the interim analysis did not lead to increased type I error rates. Moreover, it was important to determine the extra required sample size, if any, as soon as possible in order to promptly begin the second stage of recruitment and expand the sample while there were still active participants from the initial stage.

Both the attrition rate and the drop-in/drop-out rate were examined based on 1 year follow-up data from approximately 200 participants. The data suggested that the original assumptions made in the sample size calculations were reasonable. However, there was potential variability in the estimates since partial data were used in the analysis. Consequently, the DSMB recommended increasing the sample size up to 1000 participants. The final sample size is 977 participants. This increased sample provided additional power for all tests, including greater than 90% power to detect a difference in cognitive decline after accounting for the estimated attrition and drop-in/drop-out rate as well as 80% power to detect a hazard ratio of 0.60 in the proportional-hazards analysis of incident cognitive impairment

13 Data management

Trained data management and study management staff at the UNC CSCC will be responsible for coordinating data management. The Data Coordinating Center (DCC) will develop a customized web-based data management system for the ACHIEVE trial, using the Carolina Data Acquisition and Reporting Tool (CDART), a state-of-the-art data management system that is currently used for ARIC-NCS. A complete description all data management procedures can be found in the MOP.

Data collection

The CSCC will lead the translation of the protocol data collection specifications into a consolidated set of clear, unambiguous data collection forms (electronic case report forms, eCRFs). Each eCRF will have a corresponding paper form to be used by sites in cases where data cannot be entered at the time of collection. Most of the CRFs have already been developed as part of ACHIEVE-P study and ARIC-NCS. Developing CRFs can uncover discrepancies within the protocol; thus, forms, the protocol, and manual of procedures will be finalized in unison during study startup.

Randomization and unblinding

Randomization, stratified by ARIC study status, field center, and hearing impairment level is completed within CDART. Although ACHIEVE hearing aid intervention is by nature un-masked, in order to minimize bias based on review of accumulating data by the project team, the ACHIEVE PI, co-investigators and key project staff except data coordinating center staff and one unblinded statistician will remain blinded to accumulating data.

Data reporting & extraction

CDART reports are run from the database in real-time and can run across multiple research sites to provide participant feedback, assist in participant visit scheduling, monitoring data quality and protocol adherence, track study enrollment, and identify medical alert values (e.g., blood pressure, depression scores, etc.).

Data security and confidentiality

CDART uses a flexible, secure authentication system requiring a username and password. In accordance with HIPAA, all individually identifiable information is encrypted and decrypted for local on-screen display at clinical centers for their own participants.

14 Data handling and record keeping

Confidentiality

Information about study subjects will be kept confidential and managed according to the requirements of the Health Insurance Portability and Accountability Act of 1996 (HIPAA). Those regulations require a signed subject authorization informing the subject of the following:

- What protected health information (PHI) will be collected from subjects in this study
- Who will have access to that information and why
- Who will use or disclose that information
- The rights of a research subject to revoke their authorization for use of their PHI

In the event that a subject revokes authorization to collect or use PHI, the investigators, by regulation, retain the ability to use all information collected prior to the revocation of subject authorization. For subjects that have revoked authorization to collect or use PHI, attempts should be made to obtain permission to collect at least vital status (i.e. that the subject is alive) at the end of their scheduled study period.

Source Documents

Source data is all information, original records of clinical findings, observations, or other activities in a clinical trial necessary for the reconstruction and evaluation of the trial. Source data are contained in source documents. Examples of these original documents, and data records include: hospital records, clinical and office charts, laboratory notes, memoranda, subjects' diaries or evaluation checklists, pharmacy dispensing records, recorded data from automated instruments, copies or transcriptions certified after verification as being accurate and complete, microfiches, photographic negatives, microfilm or magnetic media, x-rays, subject files, and records kept at the pharmacy, at the laboratories, and at medico-technical departments involved in the clinical trial.

Electronic Case Report Forms

The study electronic case report form (CRF) will be the primary data collection instrument for the study. All data requested on the CRF will be recorded and all missing data will be explained. For ease of data collection, paper forms can be used by the site, in which case the paper form becomes part of the source documents.

Records Retention

Co-PIs and field center PIs will retain study essential documents and specimens for up to 10 years following study completion.

15 Quality assurance and quality control

Established quality assurance procedures used in ARIC-NCS will be followed. Full details can be found in MOP.

15.1 Fidelity of hearing intervention

To ensure fidelity of the hearing intervention, field site training of study staff, including study audiologist and delegated hearing intervention study staff, as well as intervention delivery, receipt, and enactment will be monitored by the USF co-investigator team - Drs. Chisolm,

Eddins, Sanchez, and Arnold. Fidelity assurance begins with centralized training conducted by the USF co-investigator team. Should there be personnel changes during the course of the study, the new field site audiologist or study staff will be trained as-needed either during a site visit or remotely, depending on individual field site needs. Training will consist of informational and hands-on learning activities and will be assessed via skill observation with mock participants as well as a written examination. During the first quarter of participant testing, weekly phone calls will take place between each site and the USF team to address site-specific issues and monitor overall fidelity. During the second and third quarters, phone calls will take place biweekly, and during the fourth quarter and onward, phone calls will take place monthly. In addition, a random sample of paper data forms will be selected for chart review at regular intervals throughout the trial. Chart reviews will be conducted to verify accurate data entry from the paper source to CDART. Site visits and remote monitoring will also be conducted, determined based on sites' needs, for additional support or as supplemental training for new study audiologists. Study audiologists will be informed at centralized training that a sample of sessions will be video or audio recorded, and 10% will be chosen at random for remote review with feedback provided to the study audiologist by the USF team. Trackable documentation will be kept for accountability including certificates of study staff and study audiologist training and reports after site visits and phone calls.

15.2 Fidelity of successful aging intervention

To ensure fidelity of the Successful Aging intervention, observations of health educators will be conducted via a site visit conducted by Dr. Glynn. Health educators will be informed at centralized training that a sample of sessions will be also be audio taped; ten percent of the sessions will be chosen at random and feedback provided to the health educator by Dr. Glynn. Further, Dr. Glynn will lead monthly Successful Aging subcommittee calls to ensure the control program is being conducted in the manner outlined in the manual of procedures as well as to troubleshoot any participant-related issues.

15.3 Quality assurance of data entry

All staff involved with data collection will be required to have appropriate electronic data management system training. The training course includes a primer on using CDART for data collection and data reporting. In addition to training and providing reference documentation, the DCC may be contacted by telephone with questions about using CDART. CDART has embedded quality control measures in the application. These include reports on missing data fields and range-checks on each data field where applicable. Additional reports may be developed to check cross-form consistencies. These reports facilitate timely identification and resolution of problems in data collection and processing. The CDART User's Manual is found in MOP.

16 Study monitoring, auditing, and inspecting

Study Monitoring Plan

The Co-PIs and field center PIs will monitor the study to ensure quality and integrity of data collected. They will review study files, regulatory documents, consent forms and allocate adequate time for other study monitoring activities. Field centers will be monitored periodically by representatives from the data coordinating center. The Co-PIs and field center PIs will also ensure that the monitor or other compliance or quality assurance reviewer is given access to all the above noted study-related documents and study related facilities and has adequate space to conduct the monitoring visit.

Auditing and Inspecting

Co-PIs and field center PIs will permit study-related monitoring, audits, and inspections by the IRB, University compliance and quality assurance groups of all study related documents (e.g., source documents, regulatory documents, data collection instruments, study data etc.). PIs will ensure the capability for inspections of applicable study-related facilities.

Ethical Considerations

This study is to be conducted according to US and international standards of Good Clinical Practice (FDA Title 21 part 312 and International Conference on Harmonization guidelines), applicable government regulations and Institutional research policies and procedures.

This protocol and any amendments will be submitted to a properly constituted Institutional Review Board (IRB), in agreement with local legal prescriptions, for formal approval of the study conduct. The decision of the IRB concerning the conduct of the study will be made in writing to the investigator and a copy of this decision will be provided to the sponsor before commencement of this study.

All subjects for this study will be provided a consent form describing this study and providing sufficient information for subjects to make an informed decision about their participation in this study. This consent form will be submitted with the protocol for review and approval by the IRB for the study. The formal consent of a participant, using the IRB-approved consent form, will be obtained before that participant undergoes any study procedure. The participant will sign the consent form, and the investigator-designated research professional obtaining the consents.

17 Timeline

Project YR	1				2				3				4				5			
Quarter	1	2	3	4	1	2	3	4	1	2	3	4	1	2	3	4	1	2	3	4
Finalize Protocol	x																			
Finalize MOP/forms	x																			
Finalize Analytic Plan	x																			
Establish DSMB	x	x																		
Training		x				x				x				x				x		
Recruitment			x	x	x	x	x	x	x	x										
Intervention			x	x	x	x	x	x	x	x										
Follow-up				x	x	x	x	x	x	x	x	x	x	x	x	x	x	x	x	x
Primary Analysis																				x
Publication/Closeout																				x

*Interim analysis for sample size re-estimation will be done after 66% of study completion (expected during year 5).

18 Organization of the study

Organizational structure for year one of the ACHIEVE study is shown in Figure 1. In brief, the Steering Committee will oversee all study activities and be chaired by the two Co-PI's (Lin, Coresh) and be comprised of all committee chairs and center leads. The ACHIEVE working group committees (Chairs) include Operations (Lin/Ring), Design and Analysis (Couper/Coresh) Neurocognitive & Dementia Adjudication (Mosley/Albert), Secondary Outcomes/Ancillary Studies (Schrack/Coresh), Intervention Fidelity & Quality (Chisolm/Glynn), Publications (Deal/Coresh), DCC (Couper/Burgard). Any updates to the organizational structure will be noted in the MOP.

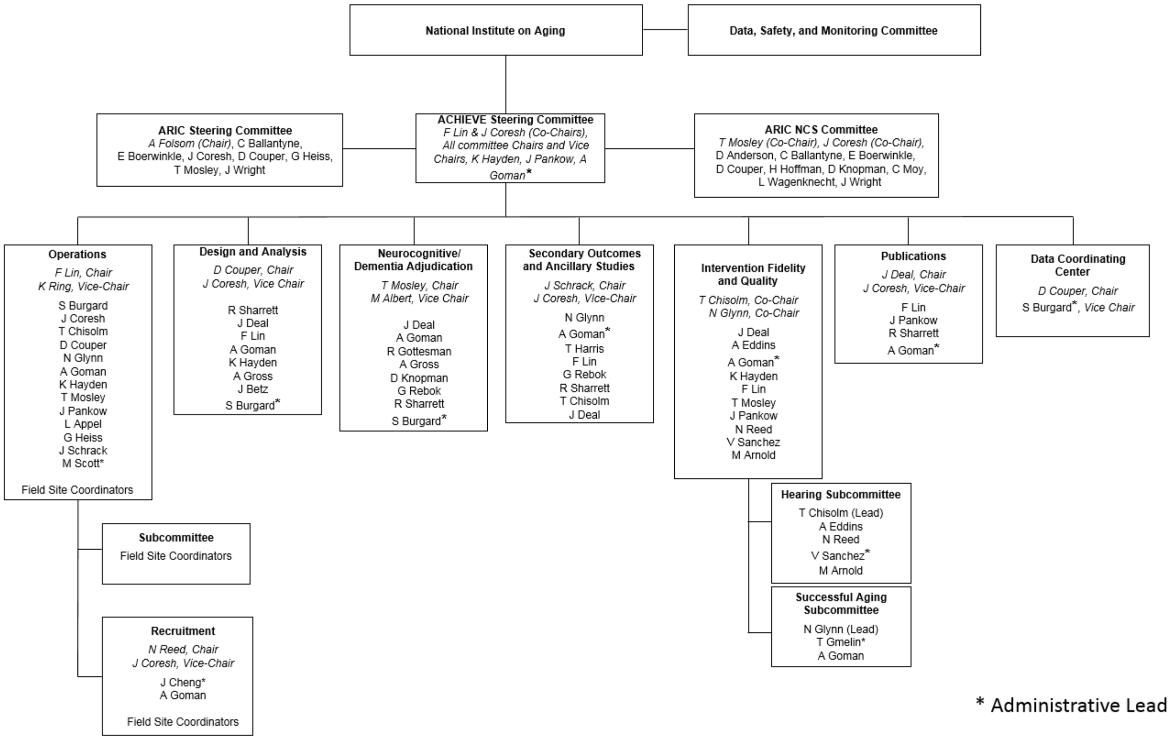

Figure 1. Organizational structure for year one of the ACHIEVE study

18.1 Key roles of committees

- Steering Committee
  - The steering committee will meet regularly to manage the study.
- Operations Committee
  - Monitoring recruitment/retention
  - Staff training
  - Development of MOP, CDART, data forms
  - Coordination with ARIC-NCS
  - Data management implementation
  - Subcommittee: Field Site Research Coordinators
  - Subcommittee: Recruitment

- Design and Analysis Committee
    - Finalize analytic plan and protocol for ACHIEVE trial
    - Develop DSMB charter and DSMP for ACHIEVE in partnership with NIA
- Neurocognitive/ Dementia Adjudication Committee
    - Oversight of neurocognitive battery administration
    - MCI/dementia adjudication
    - QA/QI of neurocognitive battery administration and collected data
- Secondary Outcomes and Ancillary Studies Committee
    - Oversight of secondary outcome battery
    - Consideration of ancillary studies
    - QA/QI of secondary battery administration and collected data
- Intervention Fidelity and Quality Committee
    - Oversight of fidelity of study interventions
    - Subcommittee: Hearing intervention
    - Subcommittee: Successful aging intervention
- Publications Committee
    - Review of all planned conference abstracts and manuscripts
- Data coordinating center
    - Management of data.
    - Organization of centralized training.

19 Changes as a result of COVID-19

The impact of the COVID-19 global pandemic has necessitated changes to the ACHIEVE protocol. This is to protect the ACHIEVE participants, ACHIEVE study staff, and the wider community from the risk of infection from COVID-19 and to adhere to institutional, state, and federal guidelines in response to the pandemic. This section details the changes as a result of COVID-19.

19.1 Remote visits

In mid-March 2020, all field sites suspended in-person study visits. Until in-person data collection is deemed safe to resume with appropriate protections in place, semi-annual and annual visits, including neurocognitive assessment, intervention booster sessions, and secondary outcome data collection, will occur via telephone or HIPAA-compliant video call (e.g., Zoom) according to the schedule shown in the table below. Specific guidance for these telephone contacts is provided in a COVID-19 MOP Appendix. For ACHIEVE participants due for their final Year 3 study visit while field site clinics were still closed, their final study visit will be a split visit that will comprise both a phone-based component on schedule and a delayed in-person component that will be conducted once field site clinics reopen. At these in-person visits, final data elements will be collected, and participants will be able to receive the other study intervention, as promised at the beginning of the trial.

Table 4. Schedule of Evaluations for Remote Data Collection

Assessment	Follow-Up Visits					
	Visit 6 (6mo)	Visit 7 (12mo) Yr1	Visit 8 (18mo)	Visit 9 (24mo) Yr2	Visit 10 (30mo)	Visit 11 (36mo) Yr3
**Covariates**						
Health History Form	X[1]	X	X[1]	X	X[1]	X
**Neurocognitive Battery**						
Ensuring speech understanding test	X	X	X	X	X	X
MMSE/Partial CDP Hybrid		X		X		X
CERAD Recall Test		X		X		X
Trail Making Test Part A [oral]		X		X		X
Trail Making Test Part B [oral]		X		X		X
Digit Span Backward		X		X		X
Word Fluency (FA)		X		X		X
Animal Naming		X		X		X
Clinical Dementia Rating Scale – Telephone Version of Participant Interview		X		X		X
Dementia/MCI evaluation if applicable[2]		X		X		X
Six-Item Screener[3]	X	X	X	X	X	X
AD8[4]	X	X	X	X	X	X
**Non-Neurocognitive Measures**						
CES-D Scale	X	X		X		X
Hearing Handicap Inventory for the Elderly - Screening Version	X	X		X		X
SF-36 Health Survey	X	X		X		X
Cohen Social Network Index	X	X		X		X
UCLA Loneliness Scale	X	X		X		X
Falls and mobility questionnaire		X		X		X
Hospitalizations	X	X	X	X	X	X
Physical Activity Form		X		X		X
Intervention Feedback Form	X					X
COVID impact questionnaire[5]			X	X	X	X
**SUCCESSFUL AGING INTERVENTION PARTICIPANTS ONLY**						
Successful aging intervention visit/booster session	X	X	X	X	X	
**HEARING INTERVENTION PARTICIPANTS ONLY**						
Intervention Check-up Form	X	X	X	X	X	X
International Outcome Inventory for comprehensive intervention	X	X	X	X	X	X

1. Only questions about hearing aid use.
2. Clinical dementia rating (informant and summary) forms and neuropsychiatric interview collected from participant's informant if participant meets criteria for additional data collection based on the neurocognitive battery.
3. Collected at the semi-annual visits. Only collected at the annual visit if unable to collect neurocognitive data.
4. Collected from knowledgeable informant at any visit only if participant is unable to complete the annual neurocognitive battery and/or the SIS.
5. Added in January 2021.

19.2 Modifications to neurocognitive assessment

19.2.1 Neurocognitive tests with no substantive changes

See section 7.2 for comparison of the in-person neurocognitive assessments. The content of the ensuring speech understanding test, the digits backwards test, and animal naming test remains the same as described in section 7.2 but with some modifications to instructions for telephone administration. The content of the CDP (see section 7.3.2) remains the same but the order of administration has been modified for use over the telephone. The word fluency test has been shortened to collect on 'F' and 'A' words. 'S' words has been dropped.

19.2.2 CERAD recall

An oral 10-item word list memory measure (CERAD, The Consortium to Establish a Registry for Alzheimer's Disease[50]) test is used in lieu of the visual Delayed Word Recall test (see section 7.2.4).

19.2.3 Shortened Mini-Mental State Exam

This includes a subset of items from the Mini-Mental State Exam (see section 7.4.10). The shortened version facilitates telephone interviewing. Two questions from the original MMSE have been adjusted for phone administration and 19 original items have been dropped. A detailed script is provided for each item.

19.2.4 Oral Trails A

An oral version of Trails A in lieu of the visual version described in Sections 7.2.7. The participant is asked to count from 1 to 25 as quickly as they can. Up to 5 attempts are allowed for this task. The total time the participant took to complete the series, the total numbers correct and the total number of errors are recorded. The maximum time permitted to complete the task is 4 minutes. If the participant makes 5 errors then the test is stopped.

19.2.5 Oral Trails B

An oral version of Trails B in lieu of the visual version described in section 7.2.8. The participant is asked to alternate between letters and numbers as quickly as they can until they reach the number 13. Up to 5 attempts are allowed for this task. The total time the participant took to complete the series, the total numbers and letters correct, and the total number of errors are recorded. The maximum time permitted to complete the task is 4 minutes. If the participant makes 5 errors then the test is stopped.

19.3 Addition of interim check-in calls

As a result of COVID-19 and its effects, the ACHIEVE team is adding interim calls to participants initiated by the interventionist (i.e., audiologist or health educator) to occur between the scheduled semi-annual and annual visits (i.e., around 9, 15, 18, 21, 27, and 33 months post-

randomization) until in-clinic visits resume. The purpose of these calls is to reinforce intervention-specific content (e.g., hearing aid use, health topics covered previously), check on participant well-being, and remind participants that the study is ongoing, though remotely, and we continue to value their contributions. These calls are considered both an additional retention tool and an adherence tool and are collected at the site's discretion.

20 History of protocol amendments

Minor changes to page numbering and formatting have been made throughout the document as part of the protocol updates. Substantive modifications between versions are detailed below.

Protocol version	Affected section	Brief description of change	Brief rationale for change
v1.7 (28 Jul 2017)	Original version of the protocol approved by site IRBs.		
v1.8 (16 Nov 2017)	4.2 Exclusion criteria	Vision criteria changed from no worse than 20/40 to no worse than 20/63.	The criteria for the ACHIEVE pilot was to read 14-point font, which corresponds to 20/63. It was always the intention to use the same criteria in ACHIEVE, but the corresponding MN acuity was inadvertently stated as 20/40 instead of 20/63.
	6.1 Informed consent	Description of re-obtaining consent in the event of cognitive impairment was added.	This information was included in the MOP but had been omitted from the protocol.
	7.5.4 SF-12v2 Health Survey	SF-12 survey replaced with SF-36 survey.	The parent study ARIC no longer uses the SF-12 due to licensing restrictions. The SF-12 is a short version of the SF-36.
	7.7 Schedule of evaluations	Table modified to reflect the correct schedule of evaluations. A pre-screening column was added, changes were made to some assessment names, and edits were made to when data on inclusion criteria and covariates are collected across pre-screening, screening, and baseline.	ACHIEVE Steering Committee decision.
	10. Strategies to promote intervention adherence	Successful aging participants "can" bring a partner changed from "encouraged" to match with the MOP.	The partner is not enrolled in the study but may accompany the participant to sessions.
	11.2 Adverse events	Addition of "Unanticipated Problems" section.	This section provides a general description of NIH criteria of unanticipated problems.
	12.1 Sample size calculation	Wording of one sentence changed from "mixed effects" to "multiple imputation ANCOVA".	This sentence was inadvertently not updated when we modified the primary analysis method from a mixed model to multiple imputation ANCOVA.

v1.9<br>(1 Jul 2020)	Study Summary, 4. Study population and eligibility	Sample size updated to reflect expanded recruitment up to 1000 participants.	The DSMB recommended extending recruitment, and the NIA issued an administrative supplement to support this change.
	5.4 Retention strategies	Clarified that study staff will determine how long to continue contact efforts, but that participants are only considered "withdrawn" if they explicitly request to withdraw.	This is a clarification.
	7.7 Schedule of evaluations	Sentence added to allow data collection visits to be split across multiple days or settings.<br><br>The ESU form is collected at all follow-up visits. The table was updated to reflect that point.	Other parts of the protocol already allowed for telephone contacts or home visits, but this information has been clearly added here to allow maximum flexibility to accommodate participants.
	9.2 Successful Aging intervention	Key #10 updated to "Caregiving and Health".	This key was previously under development and has since been finalized.
	12.4 Interim Analysis for sample size re-estimation	Updated to detail the interim analysis that was conducted in June 2019 in lieu of the originally planned interim analysis.	Changes made based on DSMB recommendations
	19. Changes as a result of COVID-19	NEW section added to outline the substantive modifications to ACHIEVE as a result of COVID-19. This section details the addition of telephone contacts and changes to outcome assessments.	The COVID-19 global pandemic led to suspension of in-person research study visits to protect participants, staff, and the larger community from risk of infection from COVID-19, which necessitated implementing remote data collection procedures.
	20. History of protocol amendments	NEW section added outlining prior substantive protocol amendments.	It is helpful to have the full history of protocol changes within the current protocol document.
v1.10<br>(9 Nov 2021)	5.4 Retention strategies	Added that telephone visits may be conducted to collect data from participants unable to come to the field site.	Telephone visits were previously added as a modality of data collection, as noted in sections 7.7 and 19 but had not been added to the retention section.
	6.1 Informed consent	Details on obtaining re-consent with a proxy, including remote consent, were added.	Due to the ongoing pandemic, the ability to obtain consent remotely from a proxy was necessary for participants who had previously provided informed consent but have been determined to have significantly reduced cognitive capacity during the course of the trial.
	6.2 Participant confidentiality	Details on the results provided to participants and their private physicians were updated.	These changes reflect corrections to match with how the study is being implemented. As ACHIEVE is a trial studying cognition, information related to cognitive impairment is

			not shared with participants. Audiometry results are not routinely shared with participants or their physicians (but can be shared with participants if requested). The only results shared directly with a participant's physician relate to alert values, and these are only shared with the physician if the participant has given approval for the results to be shared.
	7.4.12 COVID impact questionnaire; 7.7 Schedule of evaluations; 19.1 Remote visits – Table 4	The protocol is being updated to reflect the addition of the COVID impact questionnaire effective January 2021 for the semi-annual and annual visits.	This questionnaire was previously submitted and approved for use but had not yet been incorporated into the study protocol. This brief questionnaire, which was based on the National Alzheimer's Coordinating Center (NACC) COVID-19 Impact Survey, was added to understand how COVID-19 may be affecting participants.
	7.7 Schedule of evaluations	The CDP, CDI, CDS, and NPI were removed from the semi-annual visits.	The removal from the semi-annual visits is a correction to reflect that these assessments were only intended to be completed annually (and for the CDI, CDS, and NPI, only if applicable).
	7.7 Schedule of evaluations	The Client-Orientated Scale of Improvement (COSI) collection schedule was updated to reflect that the goals were only discussed during hearing intervention sessions A, C, and D, and not at the subsequent semi-annual and annual visits.	This change is a correction to reflect the proper timing of assessment as planned and implemented.
	17 Timeline	The timeline was updated.	The updates to the timeline reflect the extended recruitment period that was recommended by the DSMB and supported by an administrative supplement from the NIA.
	19.1 Remote visits	Information regarding the split Year 3 visits with a delayed in-person component after the field sites reopened.	This change, which was previously submitted and approved, is now being incorporated into the protocol. While the field sites were closed due to COVID, it was necessary to split the Year 3 visits, with a delayed in-person visit after the field sites reopened, to allow for the best quality data for the primary endpoint, consistent with the baseline in-person measurement. Having a delayed in-person visit also allowed for delivery of the other study intervention upon trial completion, as was promised to participants.

	22 Appendix A	This section was added to detail the delivery of the other intervention to participants upon their completion of the ACHIEVE Trial.	The protocol and consent stated that participants would be able to receive the other intervention (i.e., the successful aging intervention for participants originally randomized to the hearing intervention, or vice versa) upon completion of their final (Year 3) ACHIEVE visit. Although the descriptions of the interventions were previously outlined in protocol section 9 as they related to the trial, the protocol did not previously specifically address what the delivery of the other interventions would look like after participants had already completed the trial.
	23 Appendix B	This section was added to describe optional extended follow-up of the participants randomized to the successful aging group after completing the ACHIEVE trial and receiving hearing aids and the hearing intervention program.	We would like to learn how receiving hearing aids affects the participants' hearing and communication, as well as social, mental, physical, and overall well-being. We would also like to understand how much participants use their hearing aids and the hearing care support from the study audiologists. Participants will be asked to provide consent to (a) allow the study to use information related to the delivery of their hearing aids and hearing support as part of their research record, and (b) participate in additional data collection assessments similar to what has been collected during their ACHIEVE visits.

21 References

1. Newman AB, Bayles CM, Milas CN, et al. The 10 Keys to Healthy Aging: Findings from an innovative prevention program in the community. *J Aging Health*. 2010;22(5):547-566.

2. Fielding RA, Rejeski WJ, Blair S, et al. The lifestyle interventions and independence for elders study: Design and methods. *J Gerontol - Ser Biol Sci Med Sci*. 2011;66 A(11):1226–1237. doi:10.1093/gerona/glr123

3. The ARIC Investigators. The Atherosclerosis Risk in Communities (ARIC) Study: design and objectives. *Am J Epidemiol*. 1989;129(4):687-702.

4. American Academy of Audiology. American Academy of Audiology Task Force: Guidelines for the audiological management of adult hearing impairment. *Audio Today*. 2006;18:32-36.

5. Hornsby B, Mueller G. Monosyllabic word testing: Five simple steps to improve accuracy and efficiency. AudiologyOnline, Article #11978. http://www.audiologyonline.com/

6. Killion MC, Niquette PA, Gudmundsen GI, Revit LJ, Banerjee S. Development of a quick speech-in-noise test for measuring signal-to-noise ratio loss in normal-hearing and hearing-impaired listeners. *J Acoust Soc Am*. 2004;116(4 Pt 1):2395-2405.

7. Wilson RH, Mcardle RA, Smith SL. An Evaluation of the BKB-SIN, HINT, QuickSIN, and WIN Materials on Listeners With Normal Hearing and Listeners With Hearing Loss. *J Speech Lang Hear Res*. 2007;50(August 2007):844-856.

8. ARIC Visit 5 and NCS Study protocol Manual of Operations 2 - Home and Field Center Procedures. Accessed January 16, 2016. http://www.cscc.unc.edu/aric/

9. Gross AL, Power MC, Albert MS, et al. Application of Latent Variable Methods to the Study of Cognitive Decline When Tests Change over Time. *Epidemiol Camb Mass*. 2015;26(6):878-887. doi:10.1097/EDE.0000000000000379

10. Albert MS, DeKosky ST, Dickson D, et al. The diagnosis of mild cognitive impairment due to Alzheimer’s disease: Recommendations from the National Institute on Aging-Alzheimer’s Association workgroups on diagnostic guidelines for Alzheimer’s disease. *Alzheimers Dement J Alzheimers Assoc*. 2011;7(3):270-279. doi:10.1016/j.jalz.2011.03.008

11. McKhann GM, Knopman DS, Chertkow H, et al. The diagnosis of dementia due to Alzheimer’s disease: Recommendations from the National Institute on Aging-Alzheimer’s Association workgroups on diagnostic guidelines for Alzheimer’s disease. *Alzheimers Dement J Alzheimers Assoc*. 2011;7(3):263-269. doi:10.1016/j.jalz.2011.03.005

12. Folstein MF, Folstein SE, McHugh PR. “Mini-mental state”. A practical method for grading the cognitive state of patients for the clinician. *J Psychiatr Res*. 1975;12(3):189-198.

13. Radloff LS. The CES-D Scale A Self-Report Depression Scale for Research in the General Population. *Appl Psychol Meas*. 1977;1(3):385-401. doi:10.1177/014662167700100306

14. Baecke JA, Burema J, Frijters JE. A short questionnaire for the measurement of habitual physical activity in epidemiological studies. *Am J Clin Nutr*. 1982;36(5):936-942.

15. Ventry IM, Weinstein BE. Identification of elderly people with hearing problems. *ASHA*. 1983;25(7):37-42.

16. Ware J, Kosinski M, Keller SD. A 12-Item Short-Form Health Survey: construction of scales and preliminary tests of reliability and validity. *Med Care*. 1996;34(3):220-233.

17. Cohen S, J DW, Skoner DP, Rabin BS, Gwaltney JM. Social ties and susceptibility to the common cold. *JAMA*. 1997;277(24):1940-1944.

18. Russell D, Peplau L a, Cutrona CE. The revised UCLA Loneliness Scale: Concurrent and discriminant validity evidence. *J Pers Soc Psychol*. 1980;39(3):472-480. doi:10.1037/0022-3514.39.3.472

19. Schrack JA, Zipunnikov V, Goldsmith J, et al. Assessing the "physical cliff": detailed quantification of age-related differences in daily patterns of physical activity. *J Gerontol A Biol Sci Med Sci*. 2014;69(8):973-979. doi:10.1093/gerona/glt199

20. Guralnik JM, Simonsick EM, Ferrucci L, et al. A short physical performance battery assessing lower extremity function: association with self-reported disability and prediction of mortality and nursing home admission. *J Gerontol*. 1994;49(2):M85-94.

21. Morone NE, Greco CM, Moore CG, et al. A Mind-Body Program for Older Adults With Chronic Low Back Pain. *JAMA Intern Med*. 2016;15213:329–337. doi:10.1001/jamainternmed.2015.8033

22. Morone NE, Greco CM, Rollman BL, et al. The design and methods of the aging successfully with pain study. *Contemp Clin Trials*. 2012;33(2):417-425. doi:10.1016/j.cct.2011.11.012

23. Boutron I, Guittet L, Estellat C, Moher D, Hróbjartsson A, Ravaud P. Reporting methods of blinding in randomized trials assessing nonpharmacological treatments. *PLoS Med*. 2007;4(2):e61. doi:10.1371/journal.pmed.0040061

24. Senn S. Testing for baseline balance in clinical trials. *Stat Med*. 1994;13(17):1715-1726.

25. Colantuoni E, Rosenblum M. Leveraging prognostic baseline variables to gain precision in randomized trials. Stat Med. 2015 Aug 15;34(18):2602-17. doi: 10.1002/sim.6507. Epub 2015 Apr 14. Erratum in: Stat Med. 2017 Nov 30;36(27):4419. PMID: 25872751; PMCID: PMC5018399.

26. Armstrong N, Gitlin L, Parisi J, Carlson M, Rebok G, Gross A. E pluribus unum: Harmonization of physical functioning across intervention studies of middle-aged and older adults. PLoS One. 2017;12(7):e0181746.

27. Chan K, Gross A, Pezzin L, Brandt J, Kasper J. Harmonizing measures of cognitive performance across international surveys of aging using item response theory. Journal of Aging and Health. 2015;27(8):1392-1414.

28. Klahr S, Levey A, Beck G, et al. The Effects of Dietary Protein Restriction and Blood-Pressure Control on the Progression of Chronic Renal Disease. The New England Journal of Medicine.1994; 330:877-884. DOI: 10.1056/NEJM199403313301301

29. Van Buuren S. Multiple imputation of discrete and continuous data by fully conditional specification. Statistical Methods in Medical Research. 2007; 16(3): p. 219-242.

30. von Hippel P. How many imputations do you need? A two-stage calculation using a quadratic rule. Sociological Methods & Research. 2018: p. 1-20.

31. Crane, P. K., Narasimhalu, K., Gibbons, L. E., Mungas, D. M., Haneuse, S., Larson, E. B., Kuller, L., Hall, K., & van Belle, G. (2008). Item response theory facilitated cocalibrating cognitive tests and reduced bias in estimated rates of decline. Journal of Clinical Epidemiology, 61(10), 1018–27.e9. doi:10.1016/j.jclinepi.2007.11.011

32. Gibbons, L. E., Feldman, B. J., Crane, H. M., Mugavero, M., Willig, J. H., Patrick, D., Schumacher, J., Saag, M., Kitahata, M. M., & Crane, P. K. (2011). Migrating from a legacy fixed-format measure to CAT administration: calibrating the PHQ-9 to the PROMIS depression measures. Quality of Life Research, 20(9), 1349–1357. doi:10.1007/s11136-011-9882-y.

33. Gross AL, Sherva R, Mukherjee S, et al. Calibrating longitudinal cognition in Alzheimer's disease across diverse test batteries and datasets. *Neuroepidemiology*. 2014;43(3-4):194-205. doi:10.1159/000367970

34. Knopman DS, Griswold ME, Lirette ST, et al. Vascular imaging abnormalities and cognition: mediation by cortical volume in nondemented individuals: atherosclerosis risk in communities-neurocognitive study. *Stroke J Cereb Circ*. 2015;46(2):433-440. doi:10.1161/STROKEAHA.114.007847

35. MacKinnon, D. P. (2008). Multivariate applications series. Introduction to statistical mediation analysis. New Jersey: Taylor & Francis Group/Lawrence Erlbaum Associates.

36. Knopman DS, Gottesman RF, Sharrett AR, Wruck LM, Windham BG, Coker L, Schneider AL, Hengrui S, Alonso A, Coresh J, Albert MS, Mosley TH Jr. Mild Cognitive Impairment and Dementia Prevalence: The Atherosclerosis Risk in Communities Neurocognitive Study (ARIC-NCS). Alzheimers Dement (Amst). 2016;2:1-11. doi: 10.1016/j.dadm.2015.12.002. PMID: 26949733; PMCID: PMC4772876.

37. Davis SM. Mixed models for repeated measures with categorical time effects (MMRM). In: *Clinicals Trials with Missing Data: A Guide for Practitioners*. John Wiley & Sons; 2014.

38. Lord FM. The Relation of Test Score to the Trait Underlying the Test. *ETS Res Bull Ser*. 1952;1952(2):517-549. doi:10.1002/j.2333-8504.1952.tb00926.x

39. Gross AL, Jones RN, Fong TG, Tommet D, Inouye SK. Calibration and validation of an innovative approach for estimating general cognitive performance. *Neuroepidemiology*. 2014;42(3):144-153. doi:10.1159/000357647

40. Gross AL, Mungas DM, Crane PK, et al. Effects of education and race on cognitive decline: An integrative study of generalizability versus study-specific results. *Psychol Aging*. 2015;30(4):863-880. doi:10.1037/pag0000032

41. Curran PJ, McGinley JS, Bauer DJ, et al. A Moderated Nonlinear Factor Model for the Development of Commensurate Measures in Integrative Data Analysis. *Multivar Behav Res*. 2014;49(3):214-231. doi:10.1080/00273171.2014.889594

42. Schneider BC, Gross AL, Bangen KJ, et al. Association of vascular risk factors with cognition in a multiethnic sample. *J Gerontol B Psychol Sci Soc Sci*. 2015;70(4):532-544. doi:10.1093/geronb/gbu040

43. Sisco S, Gross AL, Shih RA, et al. The role of early-life educational quality and literacy in explaining racial disparities in cognition in late life. *J Gerontol B Psychol Sci Soc Sci*. 2015;70(4):557-567. doi:10.1093/geronb/gbt133

44. Fieo R, Mukherjee S, Dmitrieva NO, et al. Differential item functioning due to cognitive status does not impact depressive symptom measures in four heterogeneous samples of older adults. *Int J Geriatr Psychiatry*. 2015;30(9):911-918. doi:10.1002/gps.4234

45. Morris, J. C., Mohs, R. C., Rogers, H., Fillenbaum, G., & Heyman, A. (1988). Consortium to establish a registry for Alzheimer's disease (CERAD) clinical and neuropsychological assessment of Alzheimer's disease. Psychopharmacology bulletin, 24(4), 641–652.

46. Gross AL, Jones RN, Inouye SK. Development of an Expanded Measure of Physical Functioning for Older Persons in Epidemiologic Research. *Res Aging*. 2015;37(7):671-694. doi:10.1177/0164027514550834

47. Purgato M, Gross AL, Jordans MJD, de Jong JTVM, Barbui C, Tol W. Psychosocial interventions for children exposed to traumatic events in low- and middle-income countries: study protocol of an individual patient data meta-analysis. *Syst Rev*. 2014;3:34. doi:10.1186/2046-4053-3-34

48. Takane Y, Leeuw J de. On the relationship between item response theory and factor analysis of discretized variables. *Psychometrika*. 1987;52(3):393-408. doi:10.1007/BF02294363

49. van der Leeuw G, Eggermont LHP, Shi L, et al. Pain and Cognitive Function Among Older Adults Living in the Community. *J Gerontol A Biol Sci Med Sci*. 2016;71(3):398-405. doi:10.1093/gerona/glv166

50. Morris JC, Heyman A, Mohs RC, et al. The Consortium to Establish a Registry for Alzheimer’s Disease (CERAD). Part I. Clinical and neuropsychological assessment of Alzheimer’s disease. *Neurology*. 1989;39(9):1159-1165. doi:10.1212/wnl.39.9.1159

22 Appendix A: Delivery of the Other Intervention after Completing ACHIEVE Trial

22.1 Successful Aging intervention for participants originally randomized to the hearing intervention group

22.1.1 Overview

Upon completion of the Year 3 ACHIEVE visit, all participants from the hearing intervention group will be offered a binder containing the Successful Aging intervention program materials on topics important for healthy aging (see section 9.2 Successful Aging intervention for an overview of the program).

Participants will be offered that they can choose to receive a follow-up call from the health educator. They can also choose to schedule an in-person session with the health educator to review health goals relevant to them.

22.1.2 Schedule of activities

Completion of any follow-up calls or in-person sessions with the health educator is considered optional, at the participant's discretion. The timing of any follow-up sessions is flexible, and there are no set windows for when the sessions must occur, but are generally encouraged to occur within 1-2 months of receiving the binder.

22.2 Hearing intervention for participants originally randomized to the Successful Aging intervention group

22.2.1 Overview

Upon completion of the Year 3 ACHIEVE visit, all participants from the successful aging intervention group will be offered to receive the comprehensive hearing intervention program, including hearing aids and other devices customized to participants' hearing needs and listening goals, along with follow-up care for 6 months.

22.2.2 Blended telehealth hearing health care delivery model

Participants receiving the hearing intervention program after completion of the successful aging program will be offered a comparable, though different, hearing intervention program than the participants initially randomized to the hearing intervention program. These participants will receive hearing aids that are compatible with remote hearing aid programming. Participants will be encouraged to receive their hearing intervention sessions remotely via telehealth. Participants may receive these sessions in person, if preferred by the participant or if the participant does not meet the technological requirements for telehealth (i.e., internet connection, smart device).

22.2.3 Schedule of activities for delivery of the hearing intervention to SA participants after completing ACHIEVE

Assessment	*Hearing Intervention Session[a]*				6-month check-up
	A	B[b]	C[c]	D[d]	
Instructions for telehealth	X				
Hearing aid dispensing, programming, and instruction in care and use	X				
HAT dispensing	X[e]	X[e]	X[e]	X[e]	
Counseling, education, and self-management support	X	X	X	X	
SA Intervention check-up	X	X	X	X	X
International Outcome Inventory for Comprehensive Hearing Intervention (IOI-CHI)				X	
Client-Oriented Scale of Improvement (COSI) goal setting	X				
COSI goals achievement				X	
Telehealth acceptance pre-intervention (TAP)	X				

[a]Hearing intervention session A will be conducted in person. Sessions B, C, and D will be conducted remotely using telehealth unless participant or staff preference is in-person sessions.
[b]Session B will occur approximately 3 weeks after Session A.
[c]Session C will occur approximately 6 weeks after Session A.
[d]Session D will occur approximately 8 weeks after Session A.
[e]HAT dispensing is expected to occur during the hearing intervention program sessions, but the specific session(s) when it occurs may vary depending upon participant goals.

22.2.4 Procedures

The activities and assessments listed in the table above are intended to be used by the audiologists to deliver the hearing intervention. These activities and assessments, with the exception of telehealth components (instruction in telehealth and the telehealth acceptance pre-intervention questionnaire), mirror those of the hearing intervention that the participants originally randomized to the hearing intervention received.

The telehealth acceptance pre-intervention questionnaire (TAP) questionnaire assesses the participant’s comfort with and acceptance of telehealth HHC prior to start of the telehealth intervention, in order to help guide the audiologist in determining whether remote or in-person sessions may be a better fit for the participant or where additional instruction related to telehealth may be needed.

The hearing intervention program will be similar to what was described in section 9.1 above.

Session A will occur in person and involves providing an overview of the participant’s hearing loss, determining the participant’s level of comfort with telehealth, setting goals using the Client Oriented Scale of Improvement (COSI), fitting the hearing aids using best practices, providing orientation for how to use the devices, determining which sections of the ACHIEVE-HI Toolkit for Self-Management© and RLO videos best relate to the participant’s needs, and orienting the participant to eAudiology and myPhonak applications.

Session B focuses on management and troubleshooting of hearing aids and HATs, including any need for fine-tuning of device settings, and counseling for self-management, including reviewing the “Hearing Loss and Your Listening Goals” and the “Communication Strategies” Toolkit sections. It is at Session B that the intervention becomes more individualized based on the unique needs and experiences of the participant. Session B will default to a telehealth session but can be completed as an in-person session if necessary.

Session C is similar to Session B. Session C will default to a telehealth session but can be completed as an in-person session if necessary.

Session D is similar to Session C and also involves completion of the IOI-CHI.

Around 6 months after Session A, participants will return to the clinic for an in-person visit to check whether the participant has any questions or concerns related to their listening goals, the hearing aids, or hearing assistive technologies.

23 Appendix B: Extended Follow-Up of Successful Aging Participants after Delivery of the Hearing Intervention

Note: The Hearing Intervention participants are already being offered extended follow-up as part of a separate trial (Randomized Trial of Telehealth vs. Conventional Hearing Care Delivery in the ACHIEVE Study [ACHIEVE-HIFU]; R01DC019408; IRB00284937 [JHU sIRB]).

23.1 Background and rationale

As part of participation in the ACHIEVE trial, upon completion of the final Year 3 visit, participants are offered the intervention that they were not initially randomized to receive (i.e., participants who were randomized to receive the hearing intervention program are offered the successful aging education program, and vice versa – see section 22 Appendix A: Delivery of the Other Intervention after Completing ACHIEVE Trial). For the successful aging participants who receive the hearing intervention program after Year 3, we would like to learn how receiving hearing aids affects the participants' hearing and communication, as well as social, mental, physical, and overall well-being. We also want to understand how much participants use their hearing aids and the hearing care support from the study audiologists, as well as the modality of hearing care support (telehealth vs. in-person).

23.2 Participants

To be eligible for the extended follow-up, participants must:

1) have been eligible for and participated in the successful aging intervention arm of the ACHIEVE trial,
2) agree to receive the hearing intervention program, and
3) be able and willing to participate in this extended follow-up (i.e., provide separate consent for the extended follow-up).

ACHIEVE participants who have developed cognitive impairment are eligible for the extended follow-up visits if they are willing to participate and are consented with a proxy (see section 6.1 Informed consent).

23.3 Schedule of activities

	42-month	Year 4
Informed consent	X[a]	
**Hearing Intervention**		
SA Intervention check-up	X	X
International Outcome Inventory for Comprehensive Hearing Intervention	X	X
**Audiometry**		
Air conduction audiometry		X
Bone conduction audiometry		X[b]
Tympanometry		X
Word Recognition in Quiet		X
Quick Speech in Noise (unaided)		X
**Social, Mental, and Physical Outcomes**		
CES-D Scale	X	X
Hearing Handicap Inventory for the Elderly - Screening Version	X	X
RAND-36 Health Survey	X	X
Cohen Social Network Index	X	X
UCLA Loneliness Scale	X	X
Hospitalizations	X	X
Qualifying (S)AE assessment	X	X
Abbreviated neurocognitive battery[c]	X	
Full neurocognitive battery[d]		X
Clinical Dementia Rating Scale - Participant (CDP)		X
Dementia/MCI evaluation if applicable (CDI, CDS, NPI)		X[b]
Baecke activity questionnaire		X
Falls and mobility questionnaire		X
Accelerometry		X
Grip strength		X
Short Physical Performance Battery		X
**Covariates**		
Health history		X
Neurologic history		X
Anthropometry		X
Seated blood pressure		X
COVID impact questionnaire	X	X
Telehealth acceptance post-intervention (TAF)	X	X

[a]Informed consent can occur following the ACHIEVE Year 3 visit, at Hearing Intervention Session A, or at the 42-month visit prior to any data collection.
[b]Procedures that are intermittent based on protocol.
[c]Ensuring Speech Understanding (ESU) and MMSE only.
[d]ESU, MMSE, Delayed Word Recall Test, Digit Symbol Substitution Test, Incidental Learning, Trail Making Test Part A and Part B, Logical Memory I and II, Digit Span Backward, Boston Naming Test, Word Fluency (FAS), and Animal Naming.

23.4 Procedures

See section 7 Data collection and measurements for descriptions of all procedures, except for the telehealth acceptance questionnaire post-intervention. The telehealth acceptance post-intervention questionnaire (TAF) is an interviewer-administered questionnaire that assesses the

participant's comfort with and acceptance of telehealth hearing health care after starting the telehealth intervention.

As part of participation in this extended follow-up, participants will be able to continue receiving hearing follow-up care from the study audiologist (e.g., maintenance, adjustments, counseling, monitoring of listening goals) for an additional 6 months beyond what was covered as part of the delivery of the other intervention (i.e., for 1 year after receiving the hearing aids). For each participant issue, the audiologist will document the modality of support provided (e.g., telephone, video call, clinic visit, hybrid), the number and duration of the encounters, the type of issue, and its resolution.

23.5 Safety

As only one questionnaire (TAF) has been added vs. what was collected in the main ACHIEVE trial, there are no new risks associated with extended follow-up.

STATISTICAL ANALYSIS PLAN: ACHIEVE

PROTOCOL: Aging and Cognitive Health Evaluation in Elders (ACHIEVE) Randomized Trial

GRANT Number R01AG055426

NCT Number NCT03243422

SAP VERSION 5.2

SAP DATE: February 23, 2023

PROTOCOL DATE: November 9, 2021 (V.1.10)

SPONSOR: National Institute on Aging

PREPARED BY: Collaborative Studies Coordinating Center
Department of Biostatistics
University of North Carolina at Chapel Hill

AUTHORS: Will be listed in order of contribution as draft is prepared

REVISIONS HISTORY:

Version	Author	Date	Description
1	David Couper, Lisa Gravens-Mueller	9/4/20	Original SAP
2	David Couper, James Pike, Lisa Gravens-Mueller	4/15/21	Updated SAP due to COVID 19
3	David Couper, James Pike, Lisa Gravens-Mueller	8/20/21	Additional updated due to COVID 19
4	David Couper, James Pike, Lisa Gravens-Mueller	3/14/22	Details added to clarify analytic plan
5	David Couper, James Pike, Lisa Gravens-Mueller	4/27/22	Noted in section 7.5 that results from the analysis of secondary outcomes will be included in primary outcome manuscript
5.1	David Couper, James Pike, Lisa Gravens-Mueller	11/11/22	Minor Update: Clarified definition of secondary outcome and described sensitivity analyses.
5.2	David Couper, James Pike, Lisa Gravens-Mueller	2/23/23	Minor Update: Clarified use of Hochberg modification to the Bonferroni adjustment.

Changes from version 1.0 to 2.0

Affected section	Brief description of change	Brief rationale for change
4. Study Design	Added: Each participant will be followed for 36 months; the COVID-19 pandemic made in person visits impossible from mid-2020 to mid-2021 delaying the closeout 36 month visit for many participants.	Due to COVID-19
4. Study Design	Added: Four intervention visits occur every three weeks following randomization. Routine follow-up clinic visits occur every six months until month 36. The purpose is to gather measures of primary and secondary outcomes. Starting in April 2020, remote data collection of primary and secondary outcomes became necessary due to the coronavirus pandemic (see section 7.1 for description of alternative modes of data collection). In January 2021, due to the coronavirus pandemic, it was decided participants' final study visit will be a split visit that will comprise first a phone-based component AND later an in-person component (with final cognitive assessment and receipt of the other study intervention) that will be conducted once field site clinics reopen in the future.	Due to COVID-19
7.1 General Considerations	Removed the third mode of neurocognitive data collection (Video Conferencing). Updated the calibration analysis and noted that factor analysis will be performed using MPlus 8.6 or later	It was decided not to use the video conferencing for neurocognitive data collection during COVID-19
7.3 Primary Analysis	Updated to note if a nonlinear trend is observed, the model will be adapted to include time splines. Updated the covariates to sex, race-center, and education, Cohort type (ARIC vs De novo. Removed the word "ANCOVA" from all covariates included in the ANCOVA model	Due to COVID-19 neurocognitive collection will be over the phone until in-person visits can resume
7.4 Sensitivity Analyses of the Primary Outcome	Updated one of the sensitivity analyses to look at cognitive decline based on a categorized version of time (visit) rather than continuous time in years	Due to COVID-19 the amount of follow-up time will be longer than 3 years for some participants.

7.6 Exploratory Analyses of the Primary Outcome	Updated the Exploratory analysis looking at whether or not the intervention alter the trajectory of cognitive decline	Due to COVID-19
10. COVID-19	Added information regarding conducting the year 3 visit as a split visit.	Due to COVID-19

Changes from version 2.0 to 3.0

**Affected section**	**Brief description of change**	**Brief rationale for change**
7.1 General Considerations	Updated administration of the neurocognitive assessment to note in-person visits resumed in June 2021. Removed the paragraph on calibration.	Due to COVID-19
7.3 Primary Analysis	Noted that we will be using both the in-person and telephone based neurocognitive assessments.	Due to COVID-19
7.4 Sensitivity Analyses of the Primary Outcome	Added a new Sensitivity analysis "An analysis of a co-calibrated[10,11] global cognitive function factor scores in which the ten tests administered in-person and the six tests administered over the phone will be included in the confirmatory factor analysis model. Modeling constraints will be applied to scale the co-calibrated factor score to the same metric as the in-person factor score utilized in the primary analysis[21]. Mode of data collection (in-person, phone) will be included in the analytic model as a covariate."	Due to COVID-19
7.5 Key Secondary Outcomes	Clarified which secondary outcome would be consider the main and elaborated the definitions.	Due to COVID-19

Changes from version 3.0 to 4.0

**Affected section**	**Brief description of change**	**Brief rationale for change**
7.1, General Considerations and 7.3 Primary Analysis	Added threshold for dichotomization of hearing loss and removed the ambiguous term "stratum".	Clarified how hearing loss would be defined as a covariate in the primary analysis.
7.1 General Considerations 7.3 Primary Analysis	Removed phone-based cognitive scores and added race to the imputation model. Removed the sentence "The phone-based global cognitive function factor score will be included in the imputation model as an auxiliary variable."	A test of 35 separate models revealed that phone-based global cognitive function factor scores did not improve the performance of the imputation model. However, adding race as an auxiliary variable resulted in a small improvement.

7.3 Primary Analysis	For the primary analysis the time scale was specified as “years from the baseline.”	Clarified the timeframe for cognitive decline.

Changes from version 4.0 to 5.0

**Affected section**	**Brief description of change**	**Brief rationale for change**
7.5 Secondary Outcomes	Clarified that results from the analysis of secondary outcomes will be included in the primary outcome manuscript	Specified the importance of examining intervention effects on both cognitive decline and adjudicated dementia.

Changes from version 5.0 to 5.1

**Affected section**	**Brief description of change**	**Brief rationale for change**
7.5 Secondary Outcomes	Clarified definition of secondary outcome and described sensitivity analyses.	Specified source of information for adjudicated MCI and dementia.

Changes from version 5.1 to 5.2

**Affected section**	**Brief description of change**	**Brief rationale for change**
7.7 Adjustment for Multiple Comparisons	Clarified use of Hochberg modification to the Bonferroni adjustment.	Specified that the Hochberg modification to the Bonferroni adjustment will be applied to five outcomes (1) decline in global cognitive function, (2) time to incident cognitive impairment, (3) decline in memory domain, (4), decline in executive function domain, and (5) decline in language domain.

<u>SAP Signatures</u>

DSMB Board and National Institute on Aging June 2, 2022

Principal Investigator – Frank Lin, MD, PhD May 16, 2022

Protocol Statistician – David Couper, PhD May 16, 2022

TABLE OF CONTENTS

1. Introduction 7
2. Background 7
3. Objectives 7
4. Study Design 7
   4.1 Inclusion Criteria 8
   4.2 Exclusion Criteria 9
5. Power 9
   5.1 Preliminary Analysis 9
   5.2 Evaluation of Assumptions 10
6. Analysis Populations 10
   6.1 Intent-to-Treat Population 10
   6.2 Per Protocol and Complier Population 11
7. Statistical Analysis 11
   7.1 General Considerations 11
   7.2 Primary Outcome 12
   7.3 Primary Analysis 13
   7.4 Sensitivity Analyses of the Primary Outcome 13
   7.5 Secondary Outcomes 14
   7.6 Exploratory Analyses of the Primary Outcome 14
   7.7 Adjustment for Multiple Comparisons 15
   7.8 Additional Outcomes 15
8. Global cognitive function factor score 15
9. COVID-19 Considerations 16
10. Software and Statistical Programming 16
11. References 16

LIST OF ABBREVIATIONS

ACTIVE	Advanced Cognitive Training for Independent and Vital Elderly study
AD	Alzheimer's Disease
APOE	Apolipoprotein E
ARIC	Atherosclerosis Risk in Communities
CDR	Clinical Dementia Rating
dB	decibels
DSMB	Data and Safety Monitoring Board
HealthABC	The Health, Aging, and Body Composition Study
HRQL	Health-Related Quality of Life
ITT	Intention-to-treat
MCI	Mild Cognitive Impairment
MMSE	Mini-Mental State Exam
SD	Standard deviation

1. Introduction

ACHIEVE is a randomized controlled trial. Approximately 850 participants will be randomly assigned to one of two conditions and followed for three years: hearing intervention with hearing aids or successful aging intervention. Participants will be community-dwelling adults aged 70-84 years with audiometric hearing impairment defined as a four-frequency (0.5, 1, 2, 4 kHz) pure-tone average threshold in the better-hearing ear of ≥30 decibels (dB) and <70dB. This document gives details of the planned statistical analyses.

2. Background

Novel approaches for reducing cognitive decline in older adults are needed given the aging of the population and the personal, socioeconomic, and public health implications of cognitive impairment and dementia in older adults. Epidemiologic data now strongly suggest that age-related peripheral hearing loss in older adults is independently associated with accelerated cognitive decline and incident dementia[1-4]. Mechanistic pathways that could underlie this observed association include the effects of poor audition and distorted peripheral encoding of sound on cognitive load, brain structure, and/or reduced social engagement. These pathways may be amenable to comprehensive hearing rehabilitative treatment consisting of the use of hearing assistive technologies (hearing aids, other integrated hearing assistive devices) and rehabilitative training. To date, however, there has never been a randomized trial that has investigated whether hearing loss treatment could reduce cognitive and other functional declines in older adults.

3. Objectives

The main objective of ACHIEVE is to determine the effect of a hearing intervention versus a successful aging control intervention on the primary outcome of global cognitive decline in 70-84 year-old well-functioning and cognitively-normal adults with hearing loss. An important secondary outcome is time until cognitive impairment (defined in section 7.5). Additional secondary outcomes include decline in the cognitive domains of memory, executive function, and language. Changes in social and physical function, physical activity, and health-related quality of life (HRQL) will be examined in exploratory analyses.

Additional exploratory objectives in ACHIEVE include: 1) to investigate whether the hearing intervention alters within-individual pre- to post-intervention trajectories of cognitive decline in the subgroup of participants recruited from the Atherosclerosis Risk in Communities (ARIC) cohort who have been followed for ~30 years prior to enrollment in the ACHIEVE trial; and 2) to evaluate the effect of hearing intervention on rates of cognitive decline in individuals with different demographics, biomarkers, and Alzheimer's disease risk factors.

4. Study Design

ACHIEVE is a randomized, open-label trial comparing a hearing intervention versus a successful aging control intervention. The original plan for enrollment was to recruit 850 participants with approximately 425 in each condition. The final enrollment was expanded to 977 participants following a sample size assumption analysis (see section 5.2) conducted in June 2019. Enrollment began in November 2017. Each participant will be followed for 36 months; the COVID-19 pandemic made in-person visits impossible from mid-2020 to mid-2021 delaying the closeout 36 month visit for many participants.

Participants in the hearing intervention condition are fitted with a hearing aid, instructed to be worn on a daily basis for study duration, and attend four 1-hour intervention sessions spaced over the 2-3 months post-randomization. Re-instruction in use of devices and hearing rehabilitative strategies will be provided every 6 months.

The successful aging control intervention consists of four 1-hour intervention sessions spaced over the 2-3 months post-randomization focusing on participant-selected topics from the 10 Keys™ to Healthy Aging program and upper body stretching. Additional sessions will be provided every 6 months.

When spouses or cohabitating partners are both eligible and randomized together, they will be randomized as a unit with, arbitrarily, the first spouse/partner of the pair to be selected according to the random assignment procedure and the second spouse/partner of the pair receiving the same assignment. Spouse/partner pairs will be randomized in spouse/partner-pair specific permuted order blocks of varying sizes within strata defined by participant status (at least one spouse/partner pair in ARIC or both non-ARIC participants) and by field site.

Pre-screening will determine if an individual meets the inclusion criteria. The screening visit is used to consent participants and to determine if an individual meets audiometric, vision, and cognitive inclusion criteria. During the baseline visit assessments measures of primary and secondary outcomes will be collected.

Four intervention visits occur every three weeks following randomization. Routine follow-up clinic visits occur every six months until month 36. The purpose is to gather measures of primary and secondary outcomes. Starting in April 2020, remote data collection of primary and secondary outcomes became necessary due to the coronavirus pandemic (see section 7.1 for description of alternative modes of data collection). In January 2021, it was decided participants' final study visit will be a split visit that will comprise a phone-based component AND a subsequent in-person component (with final cognitive assessment and receipt of the other study intervention) that will be conducted once field site clinics reopen following closures prompted by the coronavirus pandemic.

4.1 Inclusion Criteria

To be eligible for the study, participants must meet all of the following criteria:

- Age 70-84 years at the time of randomization. This age range will allow recruitment of participants who are at risk for cognitive decline but who may also be expected to survive for the duration of the study.
- Community-dwelling.
- Fluent English-speaker.
- Residency. Participants must plan to reside in the local area for the study duration.
- Audiometric hearing impairment. Participants must have adult-onset hearing impairment with a four-frequency pure tone average (0.5, 1, 2, 4 kHz) in the better-hearing ear of ≥ 30 decibels and <70dB. This level of hearing impairment is the level at which individuals would be most likely to benefit from the use of conventional amplification devices such as hearing aids.
- Word recognition in quiet score ≥60% in better ear. A word recognition in quiet score <60% suggests hearing impairment that is too severe to benefit from conventional amplification devices such as hearing aids.
- Mini-Mental State Exam (MMSE) score ≥ 23 for individuals with high-school degree or less; MMSE score ≥ 25 for individuals with some college or more; Participants must be at risk for cognitive decline in the range quantified well by neurocognitive testing, and so must be free from more substantial cognitive impairment at baseline.

Participants must be willing and able to consent to participate in the study, be willing to be randomized to either the hearing intervention or to the successful aging control intervention and be willing to commit to adhere to the protocol for the duration of the study.

4.2 Exclusion Criteria

Potential candidates for enrollment who meet one or more of the following criteria are excluded from participation in the study:

- Self-reported disability in ≥ 2 or more Activities of Daily Living.
- Any self-reported hearing aid use in the past year. Participants will be randomized to hearing intervention or successful aging control intervention and, therefore, participants cannot already be receiving treatment for their hearing loss.
- ARIC participants only: Diagnosis of adjudicated dementia based on a previous ARIC visit or participant required a proxy to assist with completing informed consent and responding to questions at ARIC visit 6 or 7.
- Vision impairment (worse than 20/40 on MN Near Vision Card). Participants who cannot see (with correction) well enough to complete the neurocognitive assessment are excluded.
- Medical contraindication to use of hearing aids (e.g., draining ear). Because hearing aids will be the primary device used in the hearing intervention, participants with medical contraindications to hearing aid use are excluded.
- Conductive hearing impairment as determined by a difference in air audiometry and bone audiometry ("air-bone gap") greater than 15 dB in 2 or more contiguous frequencies in both ears. Because the impact of a conductive (versus a sensorineural) hearing loss on cognitive functioning may potentially differ and programming for hearing aids differs for conductive hearing loss, participants with permanent conductive hearing loss are excluded from the study. Participants with an air-bone gap due to fluid in the ears or other resolvable medical issue may be enrolled in the study following successful medical resolution of the cause of the air-bone gap.
- Unwilling to wear hearing aids on a regular (i.e., daily or near daily) basis.
- No participants are excluded based on race or sex.

Temporary Exclusion Criteria: Participants determined to have a conductive hearing impairment as measured by a difference in air audiometry and bone audiometry ("air-bone gap") greater than 15 dB in 2 or more contiguous frequencies in both ears are excluded from the study and referred for medical follow-up. Should the cause of the air-bone gap be determined to be due to a temporary or treatable medical issue (e.g., fluid or wax in the ears), participants may be enrolled following resolution of the issue (i.e., air-bone gap). Participants with asymmetrical sensorineural hearing (≥20 dB at 2 adjacent frequencies or ≥ 10 dB at 3 adjacent frequencies) or with other symptoms/signs concerning for a retrocochlear etiology based on the assessment of the audiologist will also not be eligible to participate until a medical clearance/evaluation is obtained.

5. Power

5.1 Preliminary Analysis

Data from the ARIC cohort, the Advanced Cognitive Training for Independent and Vital Elderly (ACTIVE) randomized trial, and the Health Aging and Body Composition (HealthABC) study were utilized to estimate that in the absence of an intervention a factor score (described in section 8.0) of global cognitive function would decline at a rate of 0.08 standard deviations (SDs) per year with a variability of 0.09 SDs. These estimates of the slope and variance were translated into expected values at Year 3. Power was conservatively estimated using a simplified t-test approach, while recognizing that greater power would be provided in the analysis of the primary outcome of cognitive decline through the application of repeated measures mixed effects models (described in section 7.3). The original sample size for ACHIEVE was 850, which provided greater than 90% power to detect a 35% difference in annual cognitive decline (Intervention: -0.052, Control: -0.08) with a 2-sided test and $p < 0.05$.

Attrition due to withdrawal or mortality was estimated at 10% per year, corresponding to 27.1% over 3 years. This is a conservative estimate given that *total* attrition in ACHIEVE is 6.7% as of October 6th, 2021. The drop-in/drop-out rate was estimated at 15% over 3 years. This rate reflects participants in the successful aging control condition who begin to wear hearing aids (drop-in) and participants in the hearing intervention condition who stop wearing hearing aids (drop-out). As of October 6th, 2021 there are 46 participants in the control condition who are currently using hearing aids (9.4% drop-in) and 8 participants in the intervention condition who are not currently using hearing aids (1.6% drop-out). After accounting for a 15% drop-in/drop-out rate, the difference at Year 3 was reduced to 29% (Intervention: -0.057, Control: -0.08). With an attrition rate of 10% per year, the initial sample size for ACHIEVE provided greater than 80% power to detect a difference utilizing a 2-sided t-test and $p < 0.05$.

For the secondary outcome of incident cognitive impairment, a dataset was constructed of participants in the ARIC cohort who had comparable characteristics to the individuals enrolled in ACHIEVE, did not exhibit signs of cognitive impairment at the baseline, and who were monitored over a period of 5 to 8 years. The observed rate was 50 cases of incident cognitive impairment per 1000 person-years. Assuming a 30% reduction in the hearing intervention condition and a 25% loss of person-time due to attrition, the proposed proportional-hazards model (described in section 7.5) would have 80% power to detect a hazard ratio of 0.54 when using a 2-sided test and $p < 0.05$.

5.2 Evaluation of Assumptions

An assumptions analysis took place in June 2019, prior to the closure of the initial recruitment period. The goal of the analysis was to evaluate a subset of the assumptions described above and assess the value of a possible increase in the sample size. Since ACHIEVE was not going to be stopped early for efficacy or futility, regardless of the interim results, and the interim analysis only investigated assumptions around attrition and drop-in/drop-out rates, not outcomes, the interim analysis did not lead to increased type I error rates. Moreover, it was important to determine the extra required sample size, if any, as soon as possible in order to promptly begin the second stage of recruitment and expand the sample while there were still active participants from the initial stage.

Both the attrition rate and the drop-in/drop-out rate were examined based on 1 year follow-up data from approximately 200 participants. The data suggested that the original assumptions made in the sample size calculations were reasonable. However, there was potential variability in the estimates since partial data were used in the analysis. Consequently, the DSMB recommended increasing the sample size up to 1000 participants. The final sample size is 977 participants. This increased sample provided additional power for all tests, including greater than 90% power to detect a difference in cognitive decline after accounting for the estimated attrition and drop-in/drop-out rate as well as 80% power to detect a hazard ratio of 0.60 in the proportional-hazards analysis of incident cognitive impairment.

6. Analysis Populations

6.1 Intent-to-Treat Population

Analyses will follow the intention-to-treat (ITT) principle in which subjects will be analyzed in the condition to which they were randomized, regardless of whether they received the assigned intervention. Primary analyses will be based on the ITT population, which includes all randomized subjects.

6.2 Per Protocol and Complier Population

A secondary analysis of the primary outcome will be completed for the per-protocol population, defined as a subset of the ITT population who completed the 2-3 months intervention period, had no hearing aid intervention drop-in for the control condition, and had no major protocol deviations. Major protocol deviations include violations in inclusion and exclusion criteria at enrollment and poor compliance with hearing aids for the hearing aid intervention condition, defined as subjects who discontinue hearing aid use. All major protocol deviations will be identified in a blinded fashion prior to database lock. Complier average causal effect analysis will also be conducted to mitigate bias present[5] in per protocol analyses.

7. Statistical Analysis

7.1 General Considerations

Study data will be monitored on an on-going basis by the ACHIEVE Data Coordinating Center (DCC). The DCC will send data clarification requests to the clinical sites for resolution while the study is on-going. Final cleaning and editing of the study database will be carried out after the final participant completes their last follow-up visit. Unblinding of treatment assignments will not be performed until the study data are cleaned, queries resolved, and database lock achieved. A permanent archive of the database will be maintained by the DCC. Randomization, stratified by ARIC study status, center, and hearing impairment level is completed within CDART, the DCC's data management system. Built-in algorithms first check for missing key items and eligibility criteria, reducing protocol violations associated with enrollment. Although the ACHIEVE hearing aid intervention is, by nature, un-masked, to minimize bias based on review of accumulating data by the project team, the ACHIEVE PI, co-investigators, and key project staff, except for key data coordinating center staff, will remain blinded to accumulating data. This will be done by applying a dummy ID onto the treatment-specific data forms as they are transferred from CDART to SAS for data cleaning and interim reporting.

This statistical analysis plan (SAP) was developed prior to review of the study dataset and under the assumption that all data were collected in adherence to the protocol and in accordance with good clinical research practices. As the data are analyzed, some deviation is anticipated (e.g., missing data; small sample sizes). In instances where these deviations would make the proposed analyses inappropriate, modifications to the analysis plan will be made and noted in the final report.

Consistent with best practices in clinical trials, we will assess the comparability of the randomized conditions with respect to known baseline confounders, such as hearing loss (pure tone average in the better hearing ear <40 db vs 40+ db), recruitment source (ARIC vs de novo), center, age, sex, education, and the presence of APOE ε4 alleles[6]. If, despite randomization, there is an imbalance between the treatment conditions we will adjust for relevant confounders as well as explore the use of a precise, locally efficient, augmented, simple estimator[7] which may offer greater precision.

At the outset of the study, an in-clinic neurocognitive assessment comprised of ten tests (see table 7.1) was administered. The assessment included the delayed word recall (exposure and recall)[8] logical memory test (I and II)[9], incidental learning test[10], word fluency test (letters F, A, and S)[11], animal naming test[11], Boston naming test[12], digit symbol substitution[9], trail making tests parts A and B[13], and the digit span backwards[9]. All ten tests were used to compute a global cognitive function factor score (described in section 8.0) for each participant[14] that was standardized to the baseline of the study.

Due to the outbreak of COVID-19 (see section 9.0 for details), a modified six-test phone-based neurocognitive assessment was administered beginning in July 2020. The assessment included the Consortium to Establish a Registry for Alzheimer's Disease (CERAD) word list (immediate and delayed)[15]

an abbreviated version of the word fluency test (letters F and A), the animal naming test, oral versions of trail making tests parts A and B, and the digit span backwards. The six tests were utilized to compute a phone-based global cognitive function factor score.

In-person administration of the original ten tests resumed in June 2021. All ACHIEVE participants will be invited to complete an in-person assessment even if the target date for the 36-month follow-up has passed. If a participant does not complete an in-person assessment, an in-person global cognitive function factor score will be imputed (see section 7.3).

Table 7.1. ACHIEVE Neurocognitive Test Battery Components

ACHIEVE in-clinic test order[1]	ACHIEVE telephone test order[1]
Ensuring Speech Understanding	Ensuring Speech Understanding for Telephone
Mini Mental State Exam (MMSE)	MMSE / CDR - Participant Hybrid for Telephone[2]
Delayed Word Recall - Exposure	CERAD Immediate[3]
Digit Symbol Substitution	Digit Span Backward
Delayed Word Recall	CERAD Delayed[3]
Incidental Learning	Oral Trails A[2]
Word Fluency (FAS)	Oral Trails B[2]
Animal Naming	Word Fluency (FA)
Logical Memory I	Animal Naming
Digit Span Backwards	
Trails A	
Trails B	
Boston Naming	
Logical Memory II	

[1]Test order changes across modes of administration to enable timing delays for memory tests.
[2]Modification applied to the in-clinic version of the neurocognitive test for telephone administration.
[3]Indicates neurocognitive test not included in the original 10-test battery.

All programs used in the statistical analysis of study data will be documented, tested, and archived. Archiving of statistical analyses at the DCC includes the original written specifications for the analyses, any subsequent modifications, the computer program file, and the log, list and other output files produced by the program. The DCC will undertake all the analyses of study data using SAS® version 9 or later. The factor analysis will be performed using Mplus 8.6 or later.

7.2 Primary Outcome

The primary outcome is cognitive decline, as measured by the change in a global cognitive function factor score. The factor score is derived from a confirmatory factor analysis model that identifies common covariation among all cognitive tests administered in-person. Factor scores are generated for each participant at each in-person assessment using a measure harmonization and item banking approach[16, 17]. Factor loadings and latent means for each neurocognitive test are estimated using data from the ACHIEVE baseline. The mean of the global cognitive function factor score at the baseline is set to 0 and the SD is set to 1. Fixed parameter estimates from the baseline model are then integrated into models that estimate the factor mean and SD at each subsequent assessment. This process ensures that changes over time represent changes in the underlying factor rather than changes in the scaling or meaning of the constructs. The assumption of longitudinal measurement invariance is supported by prior studies that utilized similar neurocognitive tests[18, 19]. A final fixed parameter model analyzes available data collected in-person and

computes a global cognitive function factor score for each participant at every assessment in which one or more neurocognitive tests were completed. A comparable process is used to generate factor scores for the cognitive domains of memory, executive functioning, and language.

7.3 Primary Analysis

We will examine cognitive decline within each condition using mixed effects models that account for the correlation among repeated measures as well as the correlation between spouses or cohabitating partners. If a linear trend appears reasonable, we will fit a model with a linear slope. If a nonlinear trend is observed, the model will be adapted to include time splines[20]. Continuous time in years from the baseline will be the time scale. An interaction term between treatment assignment and time will be used to test if rates of cognitive change differ by treatment assignment. Model fit will be assessed with residual plots and other statistics (Akaike Information Criterion, Bayesian Information Criterion, etc.). The primary analysis may include adjustments for the baseline hearing loss, ARIC vs de novo status, center, age, sex, education, and APOE ε4 alleles.

Missing cognitive factor scores among ACHIEVE participants will be generated utilizing multiple imputation by chained equations[21]. The number of imputations needed to generate valid parameter estimates will be determined by a two-stage analysis[22]. The imputation model will include (1) in-person cognitive factor scores, (2) MMSE and Six-item Screener scores, (3) adjudicated incident MCI or dementia, (4) race, (5) time variables indicating when a participant with missing data might have completed an assessment based on time from randomization to missed visit, and (6) all previously listed covariates. Interactions between variables in the imputation model will be tested and added as necessary. The imputation will be conducted in stages so that concurrent and past measurements, but not future measurements, inform the imputed values. The validity of the imputation model will be assessed by comparing observed values to imputed values among a 20% sample selected at random and a 20% sample selected based on the probability of missingness estimated from a logistic regression model.

The primary analysis will focus on cognitive factor scores imputed prior to death. An analysis comparing pre- and post-death cognitive factor scores will be performed using values generated from an imputation model in which death is included as an auxiliary variable. A similar procedure will be used to contrast pre- and post-dementia cognitive factor scores.

7.4 Sensitivity Analyses of the Primary Outcome

Additional analyses of the primary outcome may include, but are not limited to:

(a) An analysis of global cognitive function in which data from in-person cognitive evaluations will be stratified by condition before a factor score is computed for each participant. The factor scores generated will be standardized using the condition-specific baseline mean and SD.
(b) An analysis of a co-calibrated[23,24] global cognitive function factor score in which the ten tests administered in-person and the six tests administered over the phone will be included in the confirmatory factor analysis model. Modeling constraints will be applied to scale the co-calibrated factor score to the same metric as the in-person factor score utilized in the primary analysis[25]. Mode of data collection (in-person, phone) will be included in the analytic model as a covariate.
(c) Use of full-information maximum likelihood to account for missing data. Parameter estimates from these analyses will be compared to estimates from multiple imputation analyses to gauge the impact of including auxiliary variables in the imputation model.
(d) Control-based multiple imputation, in which missing values of the primary outcome for the hearing aid condition without a dementia diagnosis are imputed based on the successful aging control intervention condition. This analysis will investigate the strength of the primary analysis results to the missing at random assumption.

(e) Analysis of cognitive decline based on a categorized version of time (visits) rather than continuous time in years.
(f) A replication of the primary analyses stratified by subgroups of ARIC vs de novo participants. Interaction between intervention condition and recruitment group will be tested in additional models relative to $p < 0.10$.
(g) Exploration of the impact on the primary analysis by further adjustment for additional explanatory variables, as mentioned in section 7.1.

7.5 Secondary Outcomes

An important secondary outcome is time until a composite of (1) adjudicated dementia determined from in-person or phone-based evaluations, (2) adjudicated MCI[26] determined from in-person evaluations (3) a 3-point drop in the 30-item MMSE administered in-person, or (4) a 3-point drop in a factor score derived from the 10-item MMSE orientation subscale and 11-item Blessed scale administered over the phone and rescaled to be equivalent to the 30-item MMSE. Intervention conditions will be compared for the time until the composite outcome utilizing a discrete-time, cause-specific proportional-hazards model with a complimentary log-log link. The same baseline covariates specified for the mixed effects model will be integrated into the proportional-hazards model. Time on study will be the time scale. As a sensitivity analysis, we will examine variations of the composite outcome that include (1) adjudicated MCI from phone-based evaluations and (2) unadjudicated MCI diagnoses identified algorithmically. In exploratory analyses, we will analyze time until (1) adjudicated dementia or MCI diagnosis and (2) decline in MMSE and Blessed scores as separate events.

Three additional secondary outcomes that will be examined include decline in the cognitive domain factor scores for memory, executive function, and language[14] derived from in-person assessments. Intervention conditions will be compared using the same statistical methods described above for the primary outcome. Results from the analysis of the four secondary outcomes will be included in the primary outcome manuscript.

7.6 Exploratory Analyses of the Primary Outcome

a) **Differential practice effects by recruitment source:** The study recruited participants from ARIC and from a community sample. ARIC participants have had prior exposure to testing while non-ARIC participants have not. To address this potential limitation, we will use a mixed effects modeling framework to examine practice effects using an indicator for the first visit in each condition, allowing the practice effect parameter to vary by recruitment source and keeping the estimated intervention effect constant. We will test the fit of that model to one where the intervention effect differs by condition to evaluate whether differential practice effects by recruitment source affects the intervention condition difference. This is a test of effect modification.
b) **Does intervention alter the trajectory of cognitive decline:** In the subset of ARIC participants, we will model prior cognitive change (before the baseline ACHIEVE visit) as well as prospective change through study end (2021-22) using linear spline models, to determine if the rate of change of cognitive decline during ACHIEVE in each condition is different than the rate of change in the preceding years in ARIC visit 5 and 6. We will also test the interaction hypothesis that the hearing intervention deceleration in decline is larger than that in the successful aging intervention condition. The power for analyses in the ARIC subgroup will be lower than optimal since the proportion of ACHIEVE participants recruited from ARIC was smaller than originally anticipated.
c) **Subgroup analyses by demographics:** Given the lower prevalence of hearing loss in women compared to men and blacks compared to other races, an exploratory analysis will be conducted stratifying by sex, race, and education. We will also conduct analyses stratifying by level of hearing loss and level of baseline global cognitive function factor score. Interactions between intervention condition and subgroups will be tested in additional models relative to $p < 0.10$.

d) **Subgroup analyses by AD risk factors:** We will investigate a further refinement of the primary analysis with subgroup analysis for known AD risk factors, including but not limited to ≥ 1 APOE ε4 allele as well as diabetes or hypertension. A subset of ARIC participants will be defined as more likely to be at high risk of AD based on reduced temporal lobe volume meta ROI[27] by structural MRI and no small vessel disease (ARIC Visit 5, 2011-13).

e) **Exclusion of tests with only auditory stimuli:** Among the full study cohort, we will derive a revised global cognitive function factor score excluding tests with only auditory stimuli (logical memory test and digit span backwards). We will use model constraints in a latent variable modeling framework to scale these revised factor scores to be on the same metric as the factor scores in the primary analysis[27].

f) **Mediation by social function:** We will investigate the extent to which cognitive improvements are mediated by improvements *in social function* using causal mediation methods [28].

7.7 Adjustment for Multiple Comparisons

Statistical significance for the primary outcome will be defined as $p < 0.05$. Secondary outcomes will be evaluated for statistical significance with a Hochberg modification to the Bonferroni adjustment, in which the p-values of the five outcomes will be ordered. The largest p-value will be compared relative to $p < 0.05$, and if met, all parameters will be considered significant. If not, then the second largest p-value will be assessed relative to $p < 0.05/2 = 0.025$, and if met then it and all other parameters will be considered significant, and so on for the 3rd p-value compared at 0.05/3=0.017, the fourth compared to 0.05/4 = 0.012, and the fifth compared to 0.05/5 = 0.01.

7.8 Additional Outcomes

Additional outcomes that will be examined include measures of social and physical function, physical activity, and HRQL. Analyses of these outcomes are considered exploratory in nature and will not be viewed as providing confirmatory tests of hypotheses. There will be no adjustment for multiple comparisons and p-values will be provided for descriptive purposes only.

Outcomes will be modeled continuously (outcomes transformed to account for non-normality if necessary) or categorized according to clinically-relevant cut-points[29]. Intervention conditions will be compared using the same approach adopted for the primary analysis.

8. Global cognitive function factor score

The global cognitive function factor score uses all available cognitive test data, has interval-level properties[30], is internally consistent using ARIC data (Cronbach's alpha=0.87), has minimal floor or ceiling effects[31], and demonstrates reliable measurement precision over a broad range of cognitive ability. We have previously demonstrated criterion validity and established cut-points for clinically relevant impairment of the general cognitive performance factor score[31]. We have since extended the harmonization to 26 studies with over 60,000 people, most of which have longitudinal data. Using simulation, we verified the cognitive metric is the same across datasets[31]. We compared precision of our approach with other approaches to combining data using external data: underscoring enhanced precision in a sample of 10,875 persons in 9 datasets, our approach required the smallest sample size to detect cognitive decline with 80% power (N=232) compared to using only the MMSE (N=277) or summarizing available tests into a z-score (N=291)[27, 32]. We further validated the approach against change in hippocampal volume and overall cortical thickness[27]. The approach is consistent with other harmonization techniques and has been used in several other published studies [25, 31-36] including demonstrations that the factors have the same meaning across datasets with different cognitive tests [15, 25, 31-35, 37-40].

9. COVID-19 Considerations

ACHIEVE completed randomization on October 25, 2019 prior to the outbreak of COVID-19. ACHIEVE suspended in-person research visits and began conducting only telephone contacts in March 2020. In July 2020 ACHIEVE initiated a modified version of the in-clinic neurocognitive battery which was collected over the telephone. For ACHIEVE participants who will become due for their final Year 3 study visit beginning in January 2021 while field site clinics are still closed, their final study visit will be a split visit that will comprise both a phone-based component (conducted at the time of their anticipated 36-month visit) AND an in-person component that will be conducted once field site clinics reopen in the future. At these in-person visits, all the data that are expected based on the 36-month visit per the protocol will be collected. There will be a minimum 3-month interval between the occurrence of a 36-month phone visit and when an in-person visit would be scheduled. For this reason some of the participants will have more than 36-months of follow-up time. Participants will be able to receive the other study intervention at the final in-person visit.

During the COVID-19 quarantine, some ACHIEVE participants may have made less use of their hearing aids and social isolation may have impacted their cognitive status. This has the potential to reduce any effect of the intervention.

10. Software and Statistical Programming

Tabulations and statistical analyses will be performed using SAS® Version 9 (or later) or Mplus® version 8.6 (or later) software. All programming will be done by the ACHIEVE Data Coordinating Center (DCC) which is housed in the Collaborative Studies Coordinating Center (CSCC) at the University of North Carolina at Chapel Hill. Standard CSCC statistical computing procedures will be followed. That is, research staff will submit a written statistical computing request to the statistical programming staff. A computing request number will be assigned to the request and information about the request entered into a database. A statistical programmer will then be assigned to undertake the request. Statisticians will review the programs and output. If necessary, changes will be requested in writing. After the programming has been completed to the satisfaction of the staff, all materials will be archived under the request number. Archived materials will include the original computing request, any subsequent changes, the SAS® or Mplus® code as well as any output, including log and list files and datasets created in the request.

11. References

1. Deal JA, Betz J, Yaffe K, et al. Hearing impairment and incident dementia and cognitive decline in older adults: the Health ABC Study. J Gerontol A Biol Sci Med Sci. May 2017;72(5):703-709. doi:10.1093/gerona/glw069

2. Lin FR, Metter EJ, O'Brien RJ, Resnick SM, Zonderman AB, Ferrucci L. Hearing loss and incident dementia. Arch Neurol. Feb 2011;68(2):214-20. doi:68/2/214 [pii] 10.1001/archneurol.2010.362

3. Livingston G, Huntley J, Sommerlad A, Ames D, Ballard C, Banerjee S, Brayne C, Burns A, Cohen-Mansfield J, Cooper C, Costafreda SG, Dias A, Fox N, Gitlin LN, Howard R, Kales HC, Kivimäki M, Larson EB, Ogunniyi A, Orgeta V, Ritchie K, Rockwood K, Sampson EL, Samus Q, Schneider LS, Selbæk G, Teri L, Mukadam N. Dementia prevention, intervention, and care: 2020 report of the Lancet Commission. Lancet. 2020 Aug 8;396(10248):413-446. doi: 10.1016/S0140-6736(20)30367-6. Epub 2020 Jul 30. PMID: 32738937; PMCID: PMC7392084.

4. Loughrey DG, Kelly ME, Kelley GA, Brennan S, Lawlor BA. Association of age-related hearing loss With cognitive function, cognitive impairment, and dementia: a systematic review and meta-analysis. JAMA Otolaryngol Head Neck Surg. 02 2018;144(2):115-126. doi:10.1001/jamaoto.2017.2513

5. Little RJ, Rubin DB. Causal effects in clinical and epidemiological studies via potential outcomes: concepts and analytical approaches. Annu Rev Public Health. 2000;21:121-45. doi: 10.1146/annurev.publhealth.21.1.121. PMID: 10884949.

6. Senn S. Testing for baseline balance in clinical trials. Stat Med. 1994;13(17):1715-1726

7. Colantuoni E, Rosenblum M. Leveraging prognostic baseline variables to gain precision in randomized trials. Stat Med. 2015 Aug 15;34(18):2602-17. doi: 10.1002/sim.6507. Epub 2015 Apr 14. Erratum in: Stat Med. 2017 Nov 30;36(27):4419. PMID: 25872751; PMCID: PMC5018399.

8. Knopman, D., & Ryberg, S. (1989). A verbal memory test with high predictive accuracy for dementia of the Alzheimer type. Archives of Neurology, 46(2), 141-145. doi:10.1001/archneur.1989.00520380041011

9. Wechsler, D. (1987). Wechsler Memory Scale-Revised. San Antonio, Texas: Psychological Corporation.

10. Ryan, J., & Lopez, S. (2001). Wechsler adult intelligence scale-III. In W. Dorfman, & M. Hersen, Understanding psychological assessment. Perspectives on individual differences. New York, NY: Kluwer Academic/Plenum Publishers.

11. Benton, A., & Hamsher, K. (1976). Multilingual Aphasia Examination. Iowa City, IA: University of Iowa.

12. Williams, B., Mack, W., & Henderson, V. (1989). Boston naming test in Alzheimer's disease. Neuropsychologia, 27(8), 1073-1079. doi:10.1016/0028-3932(89)90186-3.

13. Reitan, R. (1958). Validity of the trail making test as an indicator of organic brain damage. Perceptual and Motor Skills, 8, 271-276. doi:10.2466/PMS.8.7.271-276.

14. Gross AL, Power MC, Albert MS, et al. Application of Latent Variable Methods to the Study of Cognitive Decline When Tests Change over Time. *Epidemiol Camb Mass*. 2015;26(6):878-887. doi:10.1097/EDE.0000000000000379.

15. Morris, J. C., Mohs, R. C., Rogers, H., Fillenbaum, G., & Heyman, A. (1988). Consortium to establish a registry for Alzheimer's disease (CERAD) clinical and neuropsychological assessment of Alzheimer's disease. Psychopharmacology bulletin, 24(4), 641–652.

16. Armstrong N, Gitlin L, Parisi J, Carlson M, Rebok G, Gross A. E pluribus unum: Harmonization of physical functioning across intervention studies of middle-aged and older adults. PLoS One. 2017;12(7):e0181746.

17. Chan K, Gross A, Pezzin L, Brandt J, Kasper J. Harmonizing measures of cognitive performance across international surveys of aging using item response theory. Journal of Aging and Health. 2015;27(8):1392-1414.

18. Hayden K, Jones R, Zimmer C, et al. Factor structure of the National Alzheimer's Coordinating Centers uniform dataset neuropsychological battery: An evaluation of invariance between and within groups over time. Alzheimer Disease and Associated Disorders. 2011;25(2):128-137.

19. Park L, Gross A, McLaren D, et al. Confirmatory factor analysis of the ADNI neuropsychological battery. Brain Imaging and Behavior. 2012;6(4):528-539.

20. Klahr S, Levey A, Beck G, et al. The Effects of Dietary Protein Restriction and Blood-Pressure Control on the Progression of Chronic Renal Disease. The New England Journal of Medicine.1994; 330:877-884. DOI: 10.1056/NEJM199403313301301

21. Van Buuren S. Multiple imputation of discrete and continuous data by fully conditional specification. Statistical Methods in Medical Research. 2007; 16(3): p. 219-242.

22. von Hippel P. How many imputations do you need? A two-stage calculation using a quadratic rule. Sociological Methods & Research. 2018: p. 1-20.

23. Crane, P. K., Narasimhalu, K., Gibbons, L. E., Mungas, D. M., Haneuse, S., Larson, E. B., Kuller, L., Hall, K., & van Belle, G. (2008). Item response theory facilitated cocalibrating cognitive tests and reduced bias in estimated rates of decline. Journal of Clinical Epidemiology, 61(10), 1018–27.e9. doi:10.1016/j.jclinepi.2007.11.011

24. Gibbons, L. E., Feldman, B. J., Crane, H. M., Mugavero, M., Willig, J. H., Patrick, D., Schumacher, J., Saag, M., Kitahata, M. M., & Crane, P. K. (2011). Migrating from a legacy fixed-format measure to CAT administration: calibrating the PHQ-9 to the PROMIS depression measures. Quality of Life Research, 20(9), 1349–1357. doi:10.1007/s11136-011-9882-y.

25. Gross AL, Sherva R,Mukherjee S, et al. Calibrating longitudinal cognition in Alzheimer's disease across diverse test batteries and datasets. Neuroepidemiology. 2014;43(3-4):194-205. doi:10.1159/000367970. doi:10.1161/STROKEAHA.114.007847.

26. Knopman DS, Gottesman RF, Sharrett AR, Wruck LM, Windham BG, Coker L, Schneider AL, Hengrui S, Alonso A, Coresh J, Albert MS, Mosley TH Jr. Mild Cognitive Impairment and Dementia Prevalence: The Atherosclerosis Risk in Communities Neurocognitive Study (ARIC-NCS). Alzheimers Dement (Amst). 2016;2:1-11. doi: 10.1016/j.dadm.2015.12.002. PMID: 26949733; PMCID: PMC4772876.

27. Knopman DS, Griswold ME, Lirette ST, et al. Vascular imaging abnormalities and cognition: mediation by cortical volume in nondemented individuals: atherosclerosis risk in communities-neurocognitive study. *Stroke J Cereb Circ*. 2015;46(2):433-440. doi:10.1161/STROKEAHA.114.007847.

28. MacKinnon, D. P. (2008). Multivariate applications series. Introduction to statistical mediation analysis. New Jersey: Taylor & Francis Group/Lawrence Erlbaum Associates.

29. Davis SM. Mixed models for repeated measures with categorical time effects (MMRM). In: Clinical Trials with Missing Data: A Guide for Practitioners. John Wiley & Sons; 2014.

30. Lord FM. The Relation of Test Score to the Trait Underlying the Test. ETS Res Bull Ser. 1952;1952(2):517-549. doi:10.1002/j.2333-8504.1952.tb00926.x.

31. Gross AL, Jones RN, Fong TG, Tommet D, Inouye SK. Calibration and validation of an innovative approach for estimating general cognitive performance. Neuroepidemiology 2014;42(3):144-153. doi:10.1159/000357647.

32. Gross AL, Mungas DM, Crane PK, et al. Effects of education and race on cognitive decline: An integrative study of generalizability versus study-specific results. Psychol Aging. 2015;30(4):863-880. doi:10.1037/pag0000032. ACHIEVE Protocol, v.1.8 November 16, 2017 64.

33. Curran PJ, McGinley JS, Bauer DJ, et al. A Moderated Nonlinear Factor Model for the Development of Commensurate Measures in Integrative Data Analysis. Multivar Behav Res. 2014;49(3):214-231. doi:10.1080/00273171.2014.889594.

34. Schneider BC, Gross AL, Bangen KJ, et al. Association of vascular risk factors with cognition in a multiethnic sample. J Gerontol B Psychol Sci Soc Sci. 2015;70(4):532-544. doi:10.1093/geronb/gbu040.

35. Sisco S, Gross AL, Shih RA, et al. The role of early-life educational quality and literacy in explaining racial disparities in cognition in late life. J Gerontol B Psychol Sci Soc Sci 2015;70(4):557-567. doi:10.1093/geronb/gbt133.

36. Fieo R, Mukherjee S, Dmitrieva NO, et al. Differential item functioning due to cognitive status does not impact depressive symptom measures in four heterogeneous samples of older adults. Int J Geriatr Psychiatry. 2015;30(9):911-918. doi:10.1002/gps.4234.

37. Gross AL, Jones RN, Inouye SK. Development of an Expanded Measure of Physical Functioning for Older Persons in Epidemiologic Research. Res Aging. 2015;37(7):671-694. doi:10.1177/0164027514550834.

38. Purgato M, Gross AL, Jordans MJD, de Jong JTVM, Barbui C, Tol W. Psychosocial interventions for children exposed to traumatic events in low- and middle-income countries: study protocol of an individual patient data meta-analysis. Syst Rev. 2014;3:34. doi:10.1186/2046-4053-3-34.

39. Takane Y, Leeuw J de. On the relationship between item response theory and factor analysis of discretized variables. Psychometrika. 1987;52(3):393-408. doi:10.1007/BF02294363.

40. van der Leeuw G, Eggermont LHP, Shi L, et al. Pain and Cognitive Function Among Older Adults Living in the Community. J Gerontol A Biol Sci Med Sci. 2016;71(3):398-405. doi:10.1093/gerona/glv166.
