# Supplementary material for: A Hearing Intervention and Health-Related Quality of Life in Older Adults: A Secondary Analysis of the ACHIEVE Randomized Clinical Trial
Source: JAMA Netw Open. 2024 Nov 21;7(11):e2446591. doi: 10.1001/jamanetworkopen.2024.46591 (PMC11582982; doi:10.1001/jamanetworkopen.2024.46591)
Supplement: Supplement 2. — eFigure 1. Covariate-Adjusted Analyses Stratified by Recruitment Source (ARIC Cohort [n = 238], De Novo Cohort [n = 739]) of 3-Year Change in RAND-36 Health-Related Quality of Life Physical and Mental Health Component Summary Scores and Domain Scores by Intervention Assignment, ACHIEVE Study eFigure 2. Per-Protocol Analysis of 3-Year Change in RAND-36 Health-Related Quality of Life Physical and Mental Health Component Summary Scores and Domain Scores by Intervention Assignment, ACHIEVE study (n = 824) eFigure 3. Complier Average Causal Effect Analysis of 3-Year Change in RAND-36 Health-Related Quality of Life Physical and Mental Health Component Summary Scores and Domain Scores by Intervention Assignment, ACHIEVE study (n = 977) eFigure 4. Complete Case Analysis of 3-Year Change in RAND-36 Health-Related Quality of Life Physical and Mental Health Component Summary Scores and Domain Scores by Intervention Assignment, ACHIEVE study (n = 977) [file jamanetwopen-e2446591-s002.pdf]

Supplemental Online Content

Huang AR, Morales EG, Arnold ML, et al; for the ACHIEVE Collaborative Research Group. A hearing intervention and health-related quality of life in older adults. *JAMA Netw Open.* 2024;7(11):e2446591. doi:10.1001/jamanetworkopen.2024.46591

eFigure 1. Covariate-Adjusted Analyses Stratified by Recruitment Source (ARIC Cohort [n = 238], De Novo Cohort [n = 739]) of 3-Year Change in RAND-36 Health-Related Quality of Life Physical and Mental Health Component Summary Scores and Domain Scores by Intervention Assignment, ACHIEVE Study

eFigure 2. Per-Protocol Analysis of 3-Year Change in RAND-36 Health-Related Quality of Life Physical and Mental Health Component Summary Scores and Domain Scores by Intervention Assignment, ACHIEVE study (n = 824)

eFigure 3. Complier Average Causal Effect Analysis of 3-Year Change in RAND-36 Health-Related Quality of Life Physical and Mental Health Component Summary Scores and Domain Scores by Intervention Assignment, ACHIEVE study (n = 977)

eFigure 4. Complete Case Analysis of 3-Year Change in RAND-36 Health-Related Quality of Life Physical and Mental Health Component Summary Scores and Domain Scores by Intervention Assignment, ACHIEVE study (n = 977)

This supplemental material has been provided by the authors to give readers additional information about their work.

© 2024 Huang AR et al. *JAMA Network Open*.

eFigure 1. Covariate-Adjusted Analyses Stratified by Recruitment Source (ARIC Cohort [n=238], De Novo Cohort [n=739]) of 3-Year Change in RAND-36 Health-Related Quality of Life Physical and Mental Health Component Summary Scores and Domain Scores by Intervention Assignment, ACHIEVE Study

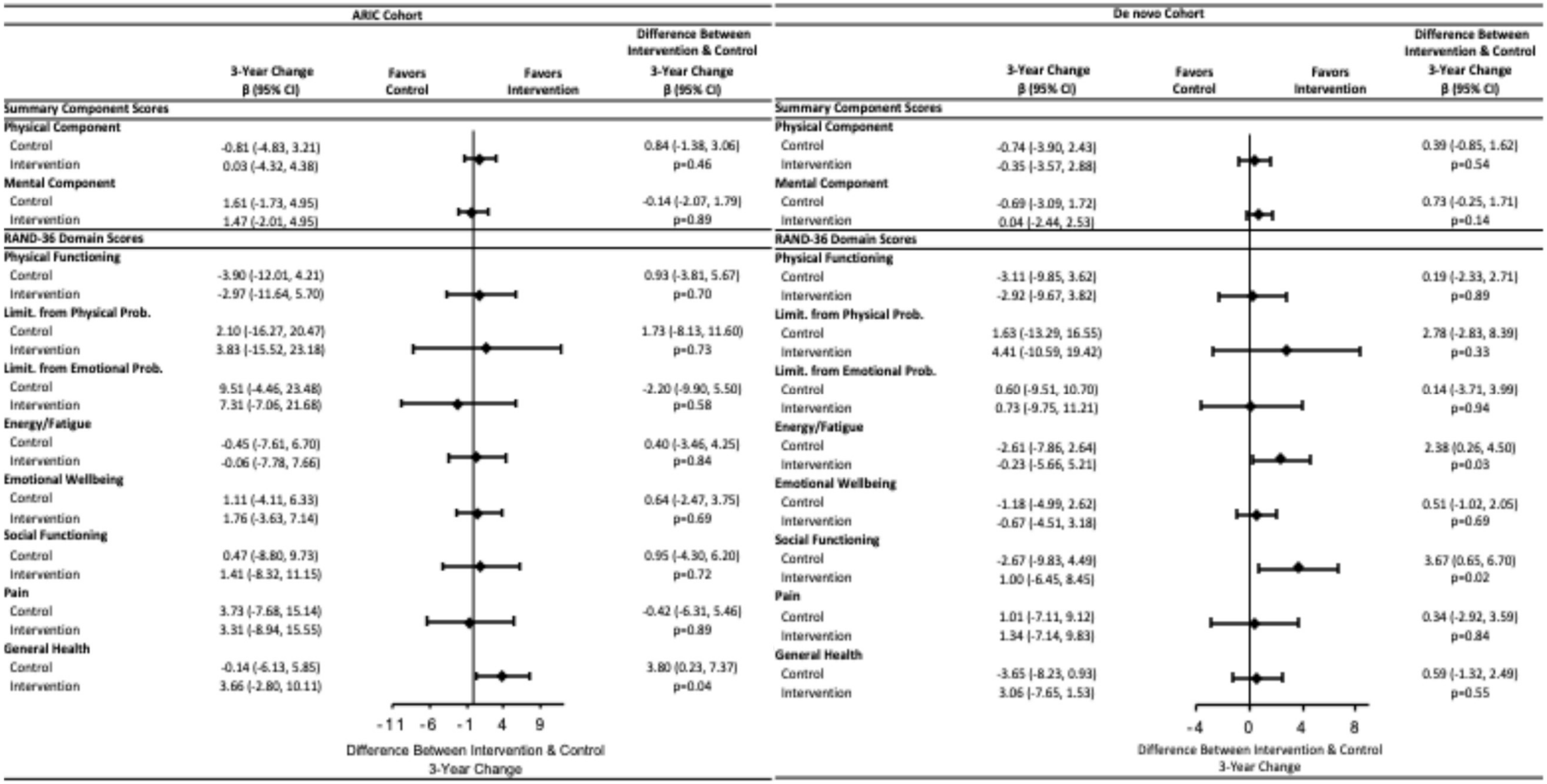

Note: Higher RAND-36 Health-Related Quality of Life domain scores and physical and mental health component scores represent better health-related quality of life. A positive value for the difference in 3-year domain scores between hearing intervention and control indicates a positive effect of hearing intervention; a negative value for the difference in 3-year domain scores between hearing intervention and control indicates a positive effect of the health education control. Models adjusted for covariates measured at baseline (age, sex, education, marital status, hearing loss severity, global cognition, field site, and whether the participant was part of a recruited spousal pair), and the interaction between time and all covariates. P-value for the 3-way interaction represents the p-value for the 3-way interaction between intervention assignment, recruitment source, and time. Abbreviations: ACHIEVE, Aging and Cognitive Health Evaluation in Elders; CI, confidence interval

© 2024 Huang AR et al. *JAMA Network Open*.

eFigure 2. Per-Protocol Analysis of 3-Year Change in RAND-36 Health-Related Quality of Life Physical and Mental Health Component Summary Scores and Domain Scores by Intervention Assignment, ACHIEVE study (n=824)

	3-Year Change β [95% CI]	Favors Control / Favors Intervention	Difference Between Intervention & Control 3-Year Change β [95% CI]
**Summary Component Scores**			
**Physical Component**			
Control	-1.08 [-3.61, 1.45]		0.51 [-0.62, 1.64]
Intervention	-0.57 [-3.13, 1.98]		p=0.38
**Mental Component**			
Control	0.82 [-1.31, 2.96]		0.26 [-0.70, 1.22]
Intervention	1.08 [-1.08, 3.24]		p=0.60
**RAND-36 Domain Scores**			
**Physical Functioning**			
Control	-4.12 [-9.46, 1.22]		0.38 [-1.94, 2.70]
Intervention	-3.74 [-9.10, 1.60]		p=0.75
**Limit. from Physical Prob.**			
Control	4.01 [-7.65, 15.68]		2.29 [-2.94, 7.52]
Intervention	6.30 [-5.56, 18.17)		p=0.39
**Limit. from Emotional Prob.**			
Control	3.25 [-5.02, 11.51]		-1.53 [-5.27, 2.20]
Intervention	1.71(-6.67, 10.09]		p=0.42
**Energy/Fatigue**			
Control	0.44 [-3.96, 4.84]		1.76 [-0.23, 3.74]
Intervention	2.19 [-2.30, 6.69]		p=0.08
**Emotional Well-being**			
Control	1.86 [-1.66, 5.39]		0.18 [-1.34, 1.69]
Intervention	2.04 [-1.48, 5.56]		p=0.82
**Social Functioning**			
Control	-1.59 [-7.84, 4.65]		2.68 [-0.11, 5.48]
Intervention	1.09 [-5.21, 7.40]		p=0.06
**Pain**			
Control	0.59 [-6.14, 7.32]		-0.44 [-3.46, 2.58]
Intervention	0.15 [-6.69, 6.98]		p=0.78
**General Health**			
Control	-2.82 [-6.79, 1.15]		1.53 [-0.29, 3.36]
Intervention	-1.29 [-5.27, 2.69]		p=0.10

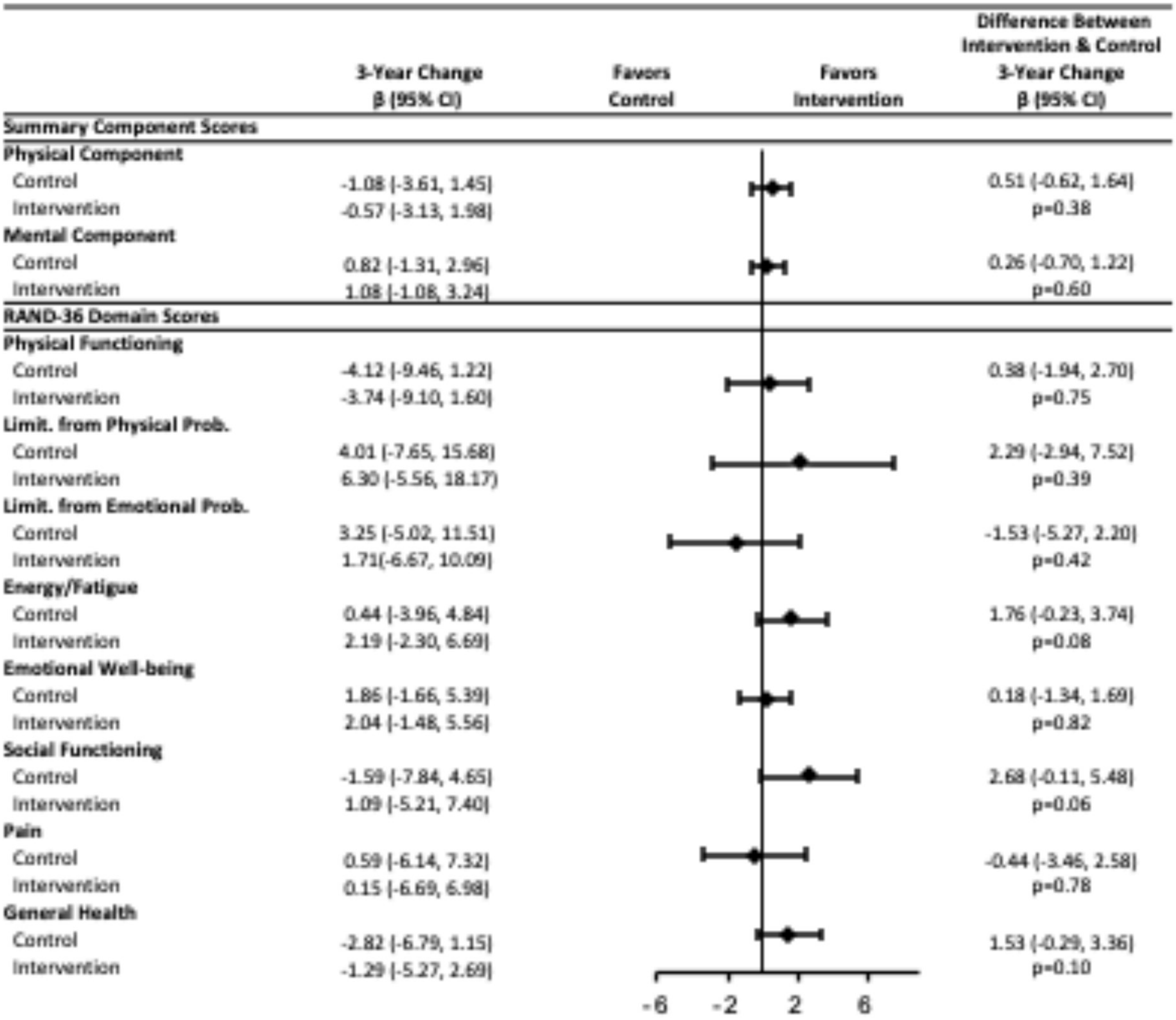

Note: Higher RAND-36 Health-Related Quality of Life domain scores and physical and mental health component scores represent better health-related quality of life. A positive value for the difference in 3-year domain scores between hearing intervention and control indicates a beneficial effect of hearing intervention; a negative value for the difference in 3-year domain scores between hearing intervention and control indicates a beneficial effect of the control. Models adjusted for covariates measured at baseline (age, sex, education, marital status, hearing loss severity, global cognition, field site, and whether the participant was part of a recruited spousal pair), and the interaction between time and all covariates. Per protocol analyses were limited to the subset of participants (Total N=824, Control N=391, Intervention N=433) who completed the intervention, had no hearing aid intervention drop-in or drop-out, and had no

© 2024 Huang AR et al. *JAMA Network Open*.

major protocol deviations. Abbreviations: ACHIEVE, Aging and Cognitive Health Evaluation in Elders; CI, confidence interval

© 2024 Huang AR et al. *JAMA Network Open*.

eFigure 3. Complier Average Causal Effect Analysis of 3-Year Change in RAND-36 Health-Related Quality of Life Physical and Mental Health Component Summary Scores and Domain Scores by Intervention Assignment, ACHIEVE study (n=977)

	3-Year Change β (95% CI)	Favors Control / Favors Intervention	Difference Between Intervention & Control 3-Year Change β (95% CI)
**Summary Component Scores**			
**Physical Component**			
Control	-0.81 (-3.27, 1.65)		0.51 (-0.71, 1.73)
Intervention	-0.30 (-2.98, 2.38)		p=0.41
**Mental Component**			
Control	0.11 (-1.77, 2.00)		0.54 (-0.46, 1.54)
Intervention	0.65 (-1.39, 2.70)		p=0.29
**RAND-36 Domain Scores**			
**Physical Functioning**			
Control	-3.50 (-8.71, 1.72)		0.37 (-2.15, 2.88)
Intervention	-3.13 (-8.72, 2.47)		p=0.78
**Limit. from Physical Prob.**			
Control	2.37 (-8.79, 13.53)		2.61 (-2.91, 8.13)
Intervention	4.98 (-6.87, 16.82)		p=0.35
**Limit. from Emotional Prob.**			
Control	2.30 (-5.46, 10.07)		-0.84 (-4.82, 3.13)
Intervention	1.46 (-6.97, 9.90)		p=0.68
**Energy/Fatigue**			
Control	-1.07 (-5.08, 2.95)		2.16 (0.05, 4.27)
Intervention	1.09 (-3.28, 5.46)		p=0.05
**Emotional Well-being**			
Control	0.19 (-2.78, 3.16)		0.60 (-0.99, 2.18)
Intervention	0.79 (-2.38, 3.95)		p=0.46
**Social Functioning**			
Control	-2.32 (-7.53, 2.88)		3.31 (0.32, 6.31)
Intervention	0.99 (-4.81, 6.79)		p=0.03
**Pain**			
Control	1.06 (-5.44, 7.56)		0.02 (-3.21, 3.25)
Intervention	1.08 (-5.94, 8.10)		p=0.99
**General Health**			
Control	-2.40 (-6.01, 1.21)		1.43 (-0.48, 3.3)
Intervention	-0.97 (-4.81, 2.86)		p=0.14

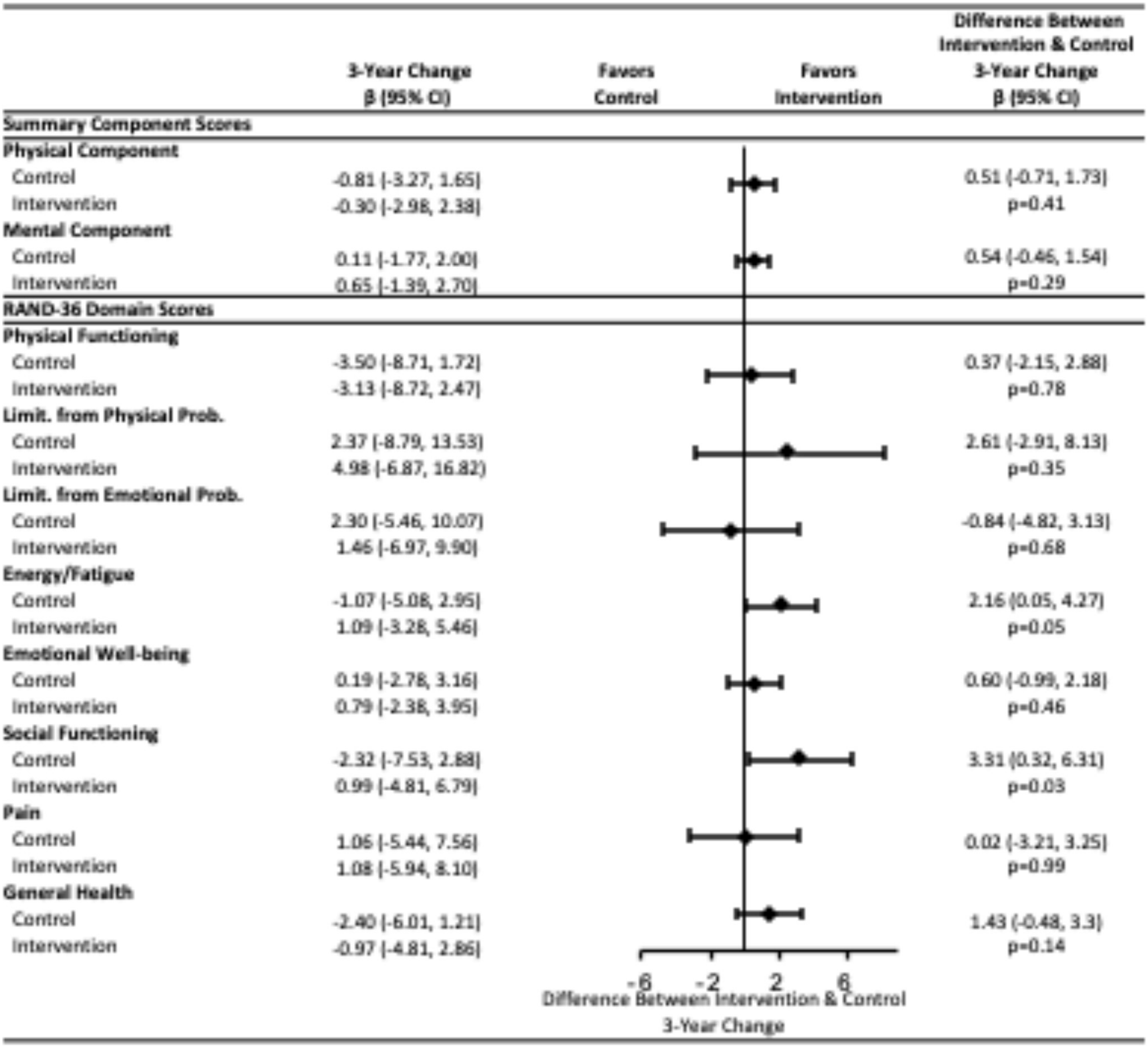

Note: Higher RAND-36 Health-Related Quality of Life domain scores and physical and mental health component scores represent better health-related quality of life. A positive value for the difference in 3-year domain scores between hearing intervention and control indicates a beneficial effect of hearing intervention; a negative value for the difference in 3-year domain scores between hearing intervention and control indicates a beneficial effect of the control. Models adjusted for covariates measured at baseline (age, sex, education, marital status, hearing loss severity, global cognition, field site, and whether the participant was part of a recruited spousal pair), and the interaction between time and all covariates. To reduce bias observed in per protocol analyses a complier average causal effect analysis was conducted in the full sample (N=977) using a two-stage least squares approach. Abbreviations: ACHIEVE, Aging and Cognitive Health Evaluation in Elders; CI, confidence interval

© 2024 Huang AR et al. *JAMA Network Open*.

eFigure 4. Complete Case Analysis of 3-Year Change in RAND-36 Health-Related Quality of Life Physical and Mental Health Component Summary Scores and Domain Scores by Intervention Assignment, ACHIEVE study (n=977)

	3-Year Change β (95% CI)	Difference Between Intervention & Control 3-Year Change β (95% CI)
**Summary Component Scores**		
**Physical Component**		
Control	-1.63 (-2.94, -0.32)	0.41 (-0.65, 1.47)
Intervention	-1.22 (-2.53, 0.09)	p=0.45
**Mental Component**		
Control	-0.85 (-2.02, 0.31)	0.51 (-0.39, 1.41)
Intervention	-0.34 (-1.51, 0.83)	p=0.26
**RAND-36 Domain Scores**		
**Physical Functioning**		
Control	-5.69 (-8.30, -3.09)	0.10 (-2.08, 2.28)
Intervention	-5.60 (-8.21, -2.98)	p=0.93
**Limit. from Physical Prob.**		
Control	-4.86 (-10.98, 1.27)	2.62 (-2.24, 7.48)
Intervention	-2.23 (-8.38, 3.91)	p=0.29
**Limit. from Emotional Prob.**		
Control	-0.16 (-4.66, 4.33)	-0.33 (-3.85, 3.19)
Intervention	-0.50 (-5.00, 4.00)	p=0.85
**Energy/Fatigue**		
Control	-5.63 (-8.01, -3.25)	1.84 (0.00, 3.69)
Intervention	-3.79 (-6.17, -1.40)	p=0.05
**Emotional Well-being**		
Control	-0.83 (-2.64, 0.98)	0.49 (-0.91, 1.88)
Intervention	-0.34 (-2.16, 1.47)	p=0.49
**Social Functioning**		
Control	-5.39 (-8.69, -2.09)	2.91 (0.30, 5.51)
Intervention	-2.48 (-5.79, 0.82)	p=0.03
**Pain**		
Control	-0.41 (-4.01, 3.19)	0.11 (-2.74, 2.96)
Intervention	-0.30 (-3.91, 3.30)	p=0.94
**General Health**		
Control	0.05 (-2.04, 2.15)	0.85 (-0.84, 2.54)
Intervention	0.91 (-1.19, 3.01)	p=0.32

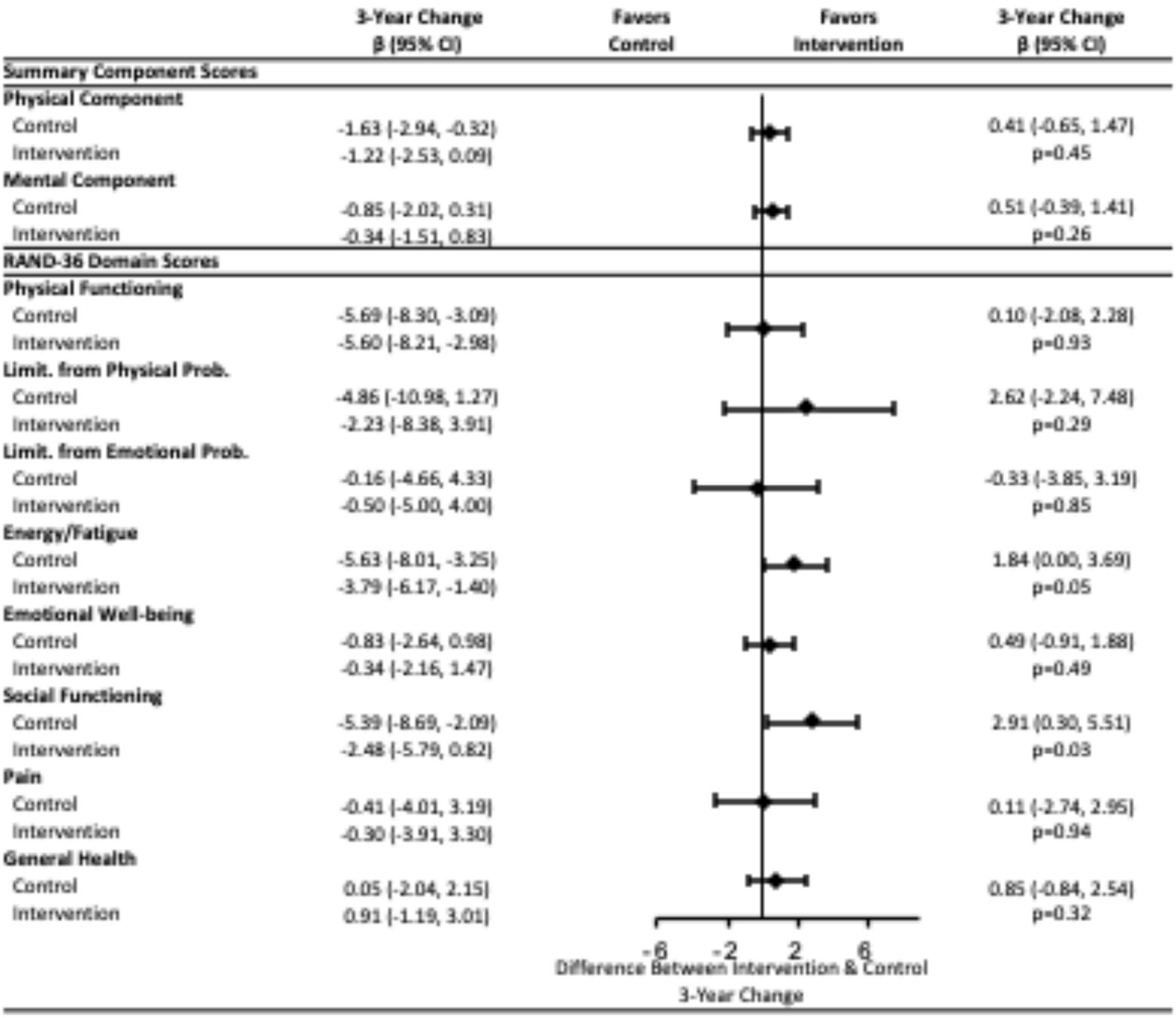

Note: Higher RAND-36 Health-Related Quality of Life domain scores and physical and mental health component scores represent better health-related quality of life. A positive value for the difference in 3-year domain scores between hearing intervention and control indicates a beneficial effect of hearing intervention; a negative value for the difference in 3-year domain scores between hearing intervention and control indicates a beneficial effect of the control. Models adjusted for covariates measured at baseline (age, sex, education, marital status, hearing loss severity, global cognition, field site, and whether the participant was part of a recruited spousal pair), and the interaction between time and all covariates. To reduce bias observed in per protocol analyses a complier average causal effect analysis was conducted in the full sample (N=977) using a two-stage least squares approach. Abbreviations: ACHIEVE, Aging and Cognitive Health Evaluation in Elders; CI, confidence interval

© 2024 Huang AR et al. *JAMA Network Open*.
