# Supplementary material for: A Hearing Intervention and Health-Related Quality of Life in Older Adults: A Secondary Analysis of the ACHIEVE Randomized Clinical Trial
Source: JAMA Netw Open. 2024 Nov 21;7(11):e2446591. doi: 10.1001/jamanetworkopen.2024.46591 (PMC11582982; doi:10.1001/jamanetworkopen.2024.46591)
Supplement: Supplement 3. — Nonauthor Collaborators. ACHIEVE Collaborative Research Group [file jamanetwopen-e2446591-s003.pdf]

Supplemental Online Content: Nonauthor Collaborators
*First name, last name, and suffix (if applicable) are required and will appear in PubMed.

*Group Name(s): ACHIEVE Collaborative Research Group							
***First Name and Middle Initial(s)**	***Last Name**	***Suffix (eg, Jr, III)**	Academic Degrees	Institution	Location (city, state/province, country)	Role or Contribution, eg, chair, principal investigator	Group (if more than 1 Group listed in the byline) and/or Subgroup (eg, Steering Committee)
Marilyn	Albert		PhD	Johns Hopkins University	Baltimore, MD, USA	Study Governance	ACHIEVE Collaborative Research Group
Joshua	Betz		MS	Johns Hopkins University	Baltimore, MD, USA	Study Governance	ACHIEVE Collaborative Research Group
Alden	Gross		PhD	Johns Hopkins University	Baltimore, MD, USA	Study Governance	ACHIEVE Collaborative Research Group
Clarice	Myers		AuD	Johns Hopkins University	Baltimore, MD, USA	Study Governance	ACHIEVE Collaborative Research Group
Richey	Sharrett		MD	Johns Hopkins University	Baltimore, MD, USA	Study Governance	ACHIEVE Collaborative Research Group
David	Li		MS	University of North Carolina	Chapel Hill, NC, USA	Data Coordinating Center	ACHIEVE Collaborative Research Group
Bria	Backman			Wake Forest University	Forsyth, NC, USA	Study Site Data Collection	ACHIEVE Collaborative Research Group
Debbie	Barr			Wake Forest University	Forsyth, NC, USA	Study Site Data Collection	ACHIEVE Collaborative Research Group
Joshua	Evans			Wake Forest University	Forsyth, NC, USA	Study Site Data Collection	ACHIEVE Collaborative Research Group
Jaime	Hampton		AuD	Wake Forest University	Forsyth, NC, USA	Study Site Data Collection	ACHIEVE Collaborative Research Group
Hailley	Humphrey-Rutledge		AuD	Wake Forest University	Forsyth, NC, USA	Study Site Data Collection	ACHIEVE Collaborative Research Group
Kaila H.	Liou		AuD	Wake Forest University	Forsyth, NC, USA	Study Site Data Collection	ACHIEVE Collaborative Research Group
Ashely	Mitchell			Wake Forest University	Forsyth, NC, USA	Study Site Data Collection	ACHIEVE Collaborative Research Group
Susan	Smith			Wake Forest University	Forsyth, NC, USA	Study Site Data Collection	ACHIEVE Collaborative Research Group

Supplemental Online Content: Nonauthor Collaborators
*First name, last name, and suffix (if applicable) are required and will appear in PubMed.

***First Name and Middle Initial(s)**	***Last Name**	***Suffix (eg, Jr, III)**	Academic Degrees	Institution	Location (city, state/province, country)	Role or Contribution, eg, chair, principal investigator	Group (if more than 1 Group listed in the byline) and/or Subgroup (eg, Steering Committee)
Nadine	Shelton			Wake Forest University	Forsyth, NC, USA	Study Site Data Collection	ACHIEVE Collaborative Research Group
Jillian	Burt		MBA	The MIND Center, University of Mississippi Medical Centerniversity	Jackson, MS, USA	Study Site Data Collection	ACHIEVE Collaborative Research Group
April	Carr			The MIND Center, University of Mississippi Medical Centerniversity	Jackson, MS, USA	Study Site Data Collection	ACHIEVE Collaborative Research Group
Arkenya	Carter		MS	The MIND Center, University of Mississippi Medical Centerniversity	Jackson, MS, USA	Study Site Data Collection	ACHIEVE Collaborative Research Group
Sarah	Faucette		AuD PhD	The MIND Center, University of Mississippi Medical Centerniversity	Jackson, MS, USA	Study Site Data Collection	ACHIEVE Collaborative Research Group
Rachel	Foster		MS	The MIND Center, University of Mississippi Medical Centerniversity	Jackson, MS, USA	Study Site Data Collection	ACHIEVE Collaborative Research Group
Ceola	Greenwood		MS	The MIND Center, University of Mississippi Medical Centerniversity	Jackson, MS, USA	Study Site Data Collection	ACHIEVE Collaborative Research Group
Temeka	Griffin		DPC	The MIND Center, University of Mississippi Medical Centerniversity	Jackson, MS, USA	Study Site Data Collection	ACHIEVE Collaborative Research Group
Candace	Jones		MSW EdS	The MIND Center, University of Mississippi Medical Centerniversity	Jackson, MS, USA	Study Site Data Collection	ACHIEVE Collaborative Research Group
Dawn	McLendon		LPN	The MIND Center, University of Mississippi Medical Centerniversity	Jackson, MS, USA	Study Site Data Collection	ACHIEVE Collaborative Research Group

Supplemental Online Content: Nonauthor Collaborators
*First name, last name, and suffix (if applicable) are required and will appear in PubMed.

***First Name and Middle Initial(s)**	***Last Name**	***Suffix (eg, Jr, III)**	Academic Degrees	Institution	Location (city, state/province, country)	Role or Contribution, eg, chair, principal investigator	Group (if more than 1 Group listed in the byline) and/or Subgroup (eg, Steering Committee)
Stacee	Naylor		MSN RN CCRP	The MIND Center, University of Mississippi Medical Centerniversity	Jackson, MS, USA	Study Site Data Collection	ACHIEVE Collaborative Research Group
Jenny	Newman		MS	The MIND Center, University of Mississippi Medical Centerniversity	Jackson, MS, USA	Study Site Data Collection	ACHIEVE Collaborative Research Group
Deidre	O'Connor		MS	The MIND Center, University of Mississippi Medical Centerniversity	Jackson, MS, USA	Study Site Data Collection	ACHIEVE Collaborative Research Group
Tiffany	Owens		MSCP	The MIND Center, University of Mississippi Medical Centerniversity	Jackson, MS, USA	Study Site Data Collection	ACHIEVE Collaborative Research Group
Jeraline	Sims		RN MSN	The MIND Center, University of Mississippi Medical Centerniversity	Jackson, MS, USA	Study Site Data Collection	ACHIEVE Collaborative Research Group
Allison	Thweatt		RN	The MIND Center, University of Mississippi Medical Centerniversity	Jackson, MS, USA	Study Site Data Collection	ACHIEVE Collaborative Research Group
Tamikia	Washington			The MIND Center, University of Mississippi Medical Centerniversity	Jackson, MS, USA	Study Site Data Collection	ACHIEVE Collaborative Research Group
Sarah	Aguilar		MS	University of Minnesota	Minneapolis, MN, USA	Study Site Data Collection	ACHIEVE Collaborative Research Group
Elizabeth	Anderson		PhD	University of Minnesota	Minneapolis, MN, USA	Study Site Data Collection	ACHIEVE Collaborative Research Group
Sydney	Boelter			University of Minnesota	Minneapolis, MN, USA	Study Site Data Collection	ACHIEVE Collaborative Research Group
Elizabeth	Penland Miller			University of Minnesota	Minneapolis, MN, USA	Study Site Data Collection	ACHIEVE Collaborative Research Group
Debbie	Ng		MPH	University of Minnesota	Minneapolis, MN, USA	Study Site Data Collection	ACHIEVE Collaborative Research Group

Supplemental Online Content: Nonauthor Collaborators
*First name, last name, and suffix (if applicable) are required and will appear in PubMed.

***First Name and Middle Initial(s)**	***Last Name**	***Suffix (eg, Jr, III)**	Academic Degrees	Institution	Location (city, state/province, country)	Role or Contribution, eg, chair, principal investigator	Group (if more than 1 Group listed in the byline) and/or Subgroup (eg, Steering Committee)
Kristi	Oeding		PhD AuD	University of Minnesota	Minneapolis, MN, USA	Study Site Data Collection	ACHIEVE Collaborative Research Group
Sandra	Potter			University of Minnesota	Minneapolis, MN, USA	Study Site Data Collection	ACHIEVE Collaborative Research Group
Katherine	Teece		AuD	University of Minnesota	Minneapolis, MN, USA	Study Site Data Collection	ACHIEVE Collaborative Research Group
Soni	Uccellini			University of Minnesota	Minneapolis, MN, USA	Study Site Data Collection	ACHIEVE Collaborative Research Group
Matthew	Waggenspack		AuD	University of Minnesota	Minneapolis, MN, USA	Study Site Data Collection	ACHIEVE Collaborative Research Group
Luanne	Welch		RN	University of Minnesota	Minneapolis, MN, USA	Study Site Data Collection	ACHIEVE Collaborative Research Group
Jacqueline	Weycker		AuD	University of Minnesota	Minneapolis, MN, USA	Study Site Data Collection	ACHIEVE Collaborative Research Group
Kerry	Witherell		AuD CCC-A	University of Minnesota	Minneapolis, MN, USA	Study Site Data Collection	ACHIEVE Collaborative Research Group
Melissa	Minotti		MPH	George W. Comstock Center for Public Health Research and Prevention, Johns Hopkins University	Washington County, MD USA	Study Site Data Collection	ACHIEVE Collaborative Research Group
Spencer	Bolton			George W. Comstock Center for Public Health Research and Prevention, Johns Hopkins University	Washington County, MD USA	Study Site Data Collection	ACHIEVE Collaborative Research Group
Laura	Sherry		AuD	George W. Comstock Center for Public Health Research and Prevention, Johns Hopkins University	Washington County, MD USA	Study Site Data Collection	ACHIEVE Collaborative Research Group
Ann C.	Eddins		PhD MBA	University of South Florida	Tampa, FL USA	Hearing Intervention Design	ACHIEVE Collaborative Research Group

Supplemental Online Content: Nonauthor Collaborators
*First name, last name, and suffix (if applicable) are required and will appear in PubMed.

***First Name and Middle Initial(s)**	***Last Name**	***Suffix (eg, Jr, III)**	Academic Degrees	Institution	Location (city, state/province, country)	Role or Contribution, eg, chair, principal investigator	Group (if more than 1 Group listed in the byline) and/or Subgroup (eg, Steering Committee)
Emily	Moore		AuD	University of South Florida	Tampa, FL USA	Hearing Intervention Design	ACHIEVE Collaborative Research Group
Haley	Neil		AuD	University of South Florida	Tampa, FL USA	Hearing Intervention Design	ACHIEVE Collaborative Research Group
Preyanca	Oree		AuD	University of South Florida	Tampa, FL USA	Hearing Intervention Design	ACHIEVE Collaborative Research Group
Laura	Westermann		MA	University of South Florida	Tampa, FL USA	Hearing Intervention Design	ACHIEVE Collaborative Research Group
Yurun	Cai		PhD	University of Pittsburgh	Pittsburgh, PA USA	Health Education Control Design	ACHIEVE Collaborative Research Group
Clifford R.	Jack		MD	Mayo Clinic	Rochester, Minnesota	MRI Reading Center	ACHIEVE Collaborative Research Group
David	Knopman		MD	Mayo Clinic	Rochester, Minnesota	MRI Reading Center	ACHIEVE Collaborative Research Group
Denise	Reyes			Mayo Clinic	Rochester, Minnesota	MRI Reading Center	ACHIEVE Collaborative Research Group
AJ	Spychalla			Mayo Clinic	Rochester, Minnesota	MRI Reading Center	ACHIEVE Collaborative Research Group
Kaely	Thostenson			Mayo Clinic	Rochester, Minnesota	MRI Reading Center	ACHIEVE Collaborative Research Group
