# Supplementary material for: A Hearing Intervention and Health-Related Quality of Life in Older Adults: A Secondary Analysis of the ACHIEVE Randomized Clinical Trial
Source: JAMA Netw Open. 2024 Nov 21;7(11):e2446591. doi: 10.1001/jamanetworkopen.2024.46591 (PMC11582982; doi:10.1001/jamanetworkopen.2024.46591)
Supplement: Supplement 4. — Data Sharing Statement [file jamanetwopen-e2446591-s004.pdf]

Data Sharing Statement

Huang. A Hearing Intervention and Health-Related Quality of Life in Older Adults. *JAMA Netw Open*. Published November 21, 2024. doi:10.1001/jamanetworkopen.2024.46591

Data
Additional Information: Trial Registration: This trial was registered at ClinicalTrials.gov, NCT03243422
Data available: Yes
Data types: Deidentified participant data
How to access data: A de-identified dataset and data dictionary will be made available in 2024 on a publicly available US data repository pending approval by the funding sponsor (National Institute on Aging). Additional details on data access policies will be made available at https://www.achievestudy.org at that time. The study protocol and statistical analysis plan are available at https://www.clinicaltrials.gov. Access to ACHIEVE study manuals and forms are available by contacting the corresponding author.
When available: beginning date: 12-31-2014

Supporting Documents
Document types: None

Additional Information
Who can access the data: researchers whose proposed use of the data has been approved
Types of analyses: for any purpose
Mechanisms of data availability: after approval of a proposal
